# Supplementary material for: Interethnic analyses of blood pressure loci in populations of East Asian and European descent
Source: Nat Commun. 2018 Nov 28;9:5052. doi: 10.1038/s41467-018-07345-0 (PMC6261994; doi:10.1038/s41467-018-07345-0)
Supplement: Supplementary file 1 — Supplementary Information [file 41467_2018_7345_MOESM1_ESM.pdf]

Supplementary Information

"Interethnic analyses of blood pressure loci in populations of East Asian and European descent"

Takeuchi *et al.*

Table of Contents

SUPPLEMENTARY FIGURES 3

SUPPLEMENTARY TABLES 37

SUPPLEMENTARY METHODS 45

1. Details of GWAS stage-1 cohorts 45

2. Details of GWAS stage-2 cohorts 47

3. Details of replication study cohort 53

4. SNP-based heritability analysis 55

SUPPLEMENTARY NOTE 1 68

Study-specific acknowledgments 68

SUPPLEMENTARY REFERENCES 72

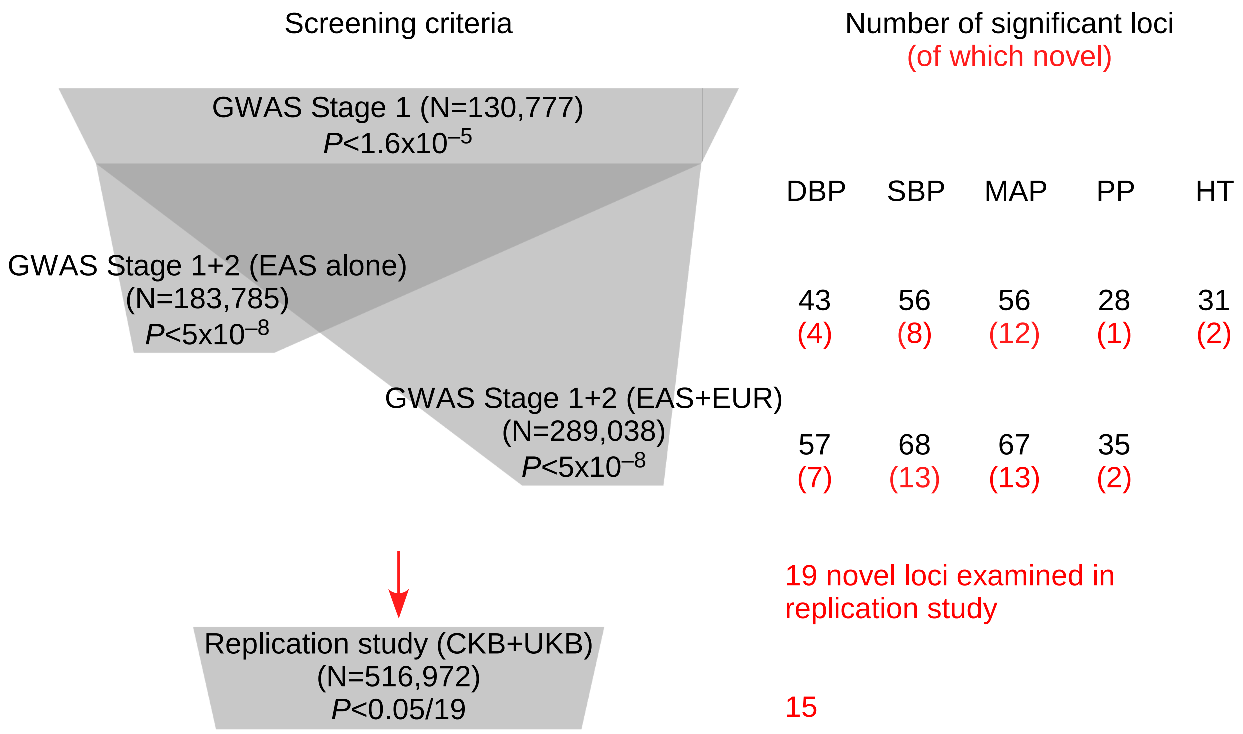

Supplementary Figure 1. Overview of study design.

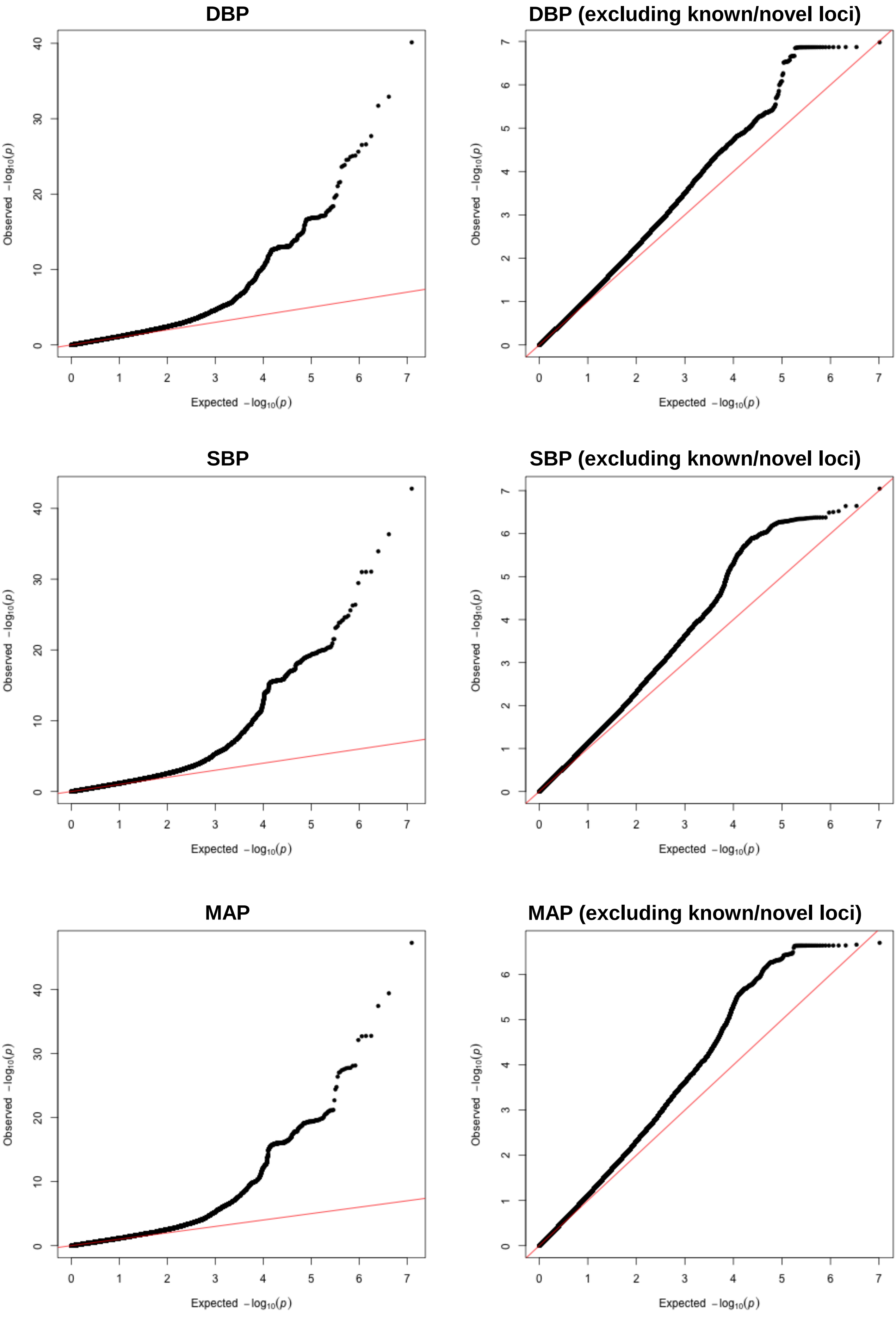

Supplementary Figure 2. Continues to next page.

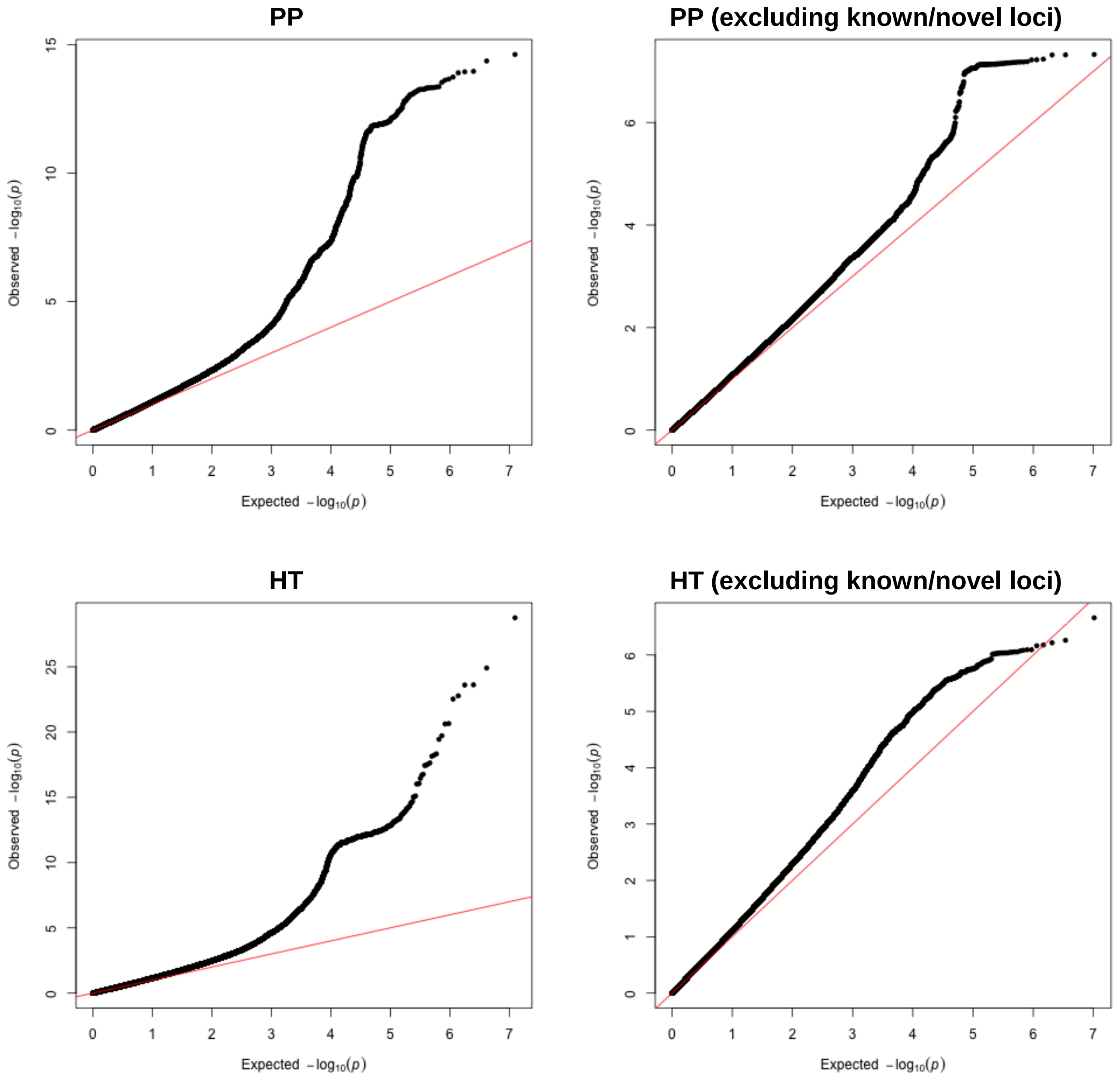

Supplementary Figure 2. Q-Q plots of five blood pressure traits for the GWAS stage-1. Q-Q plots are shown without and with subtraction of the findings from known and newly identified SNP loci.

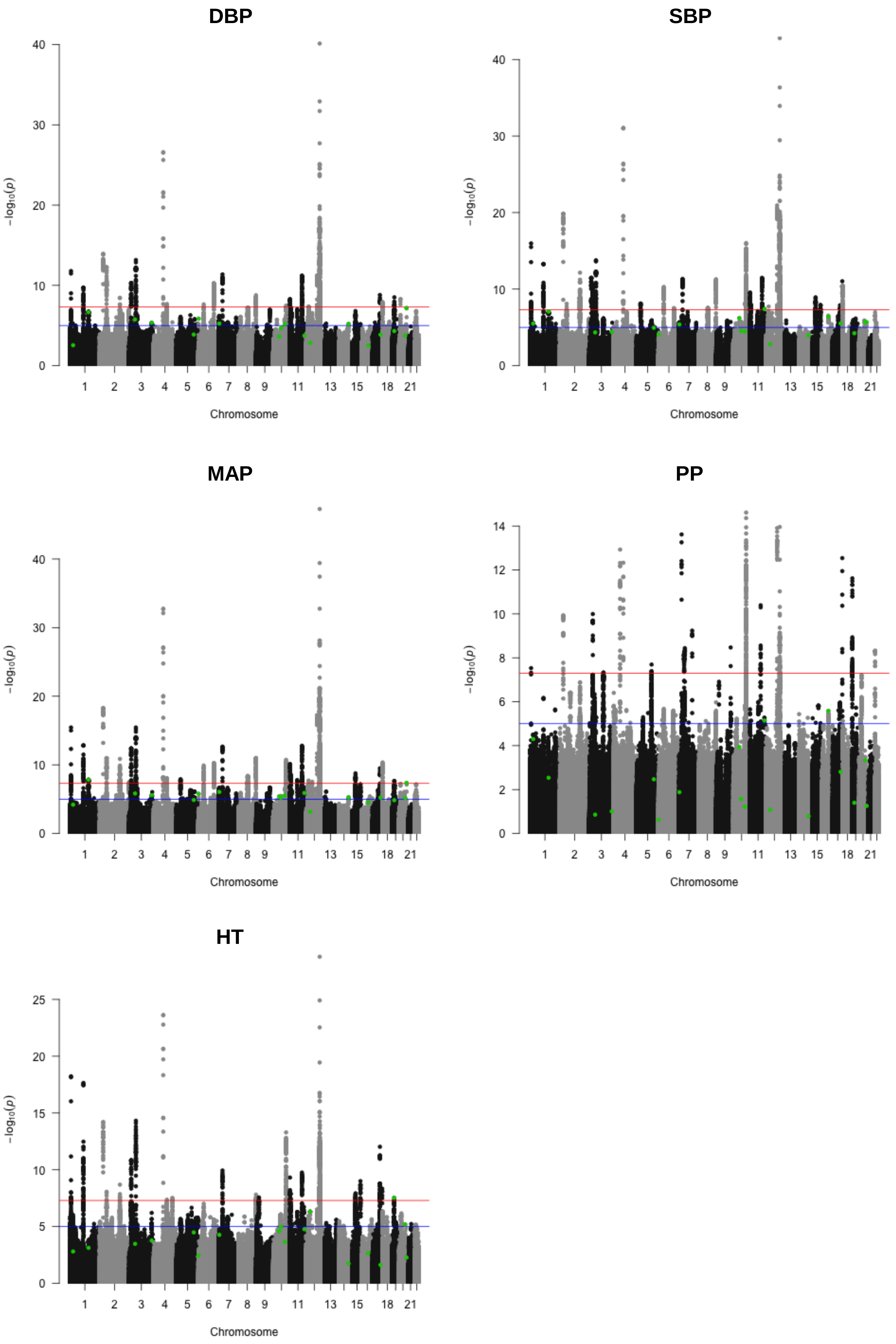

Supplementary Figure 3. Manhattan plot showing results for the GWAS. Genome-wide association results for genetic variants at the novel loci in the stage-1 are shown in green.

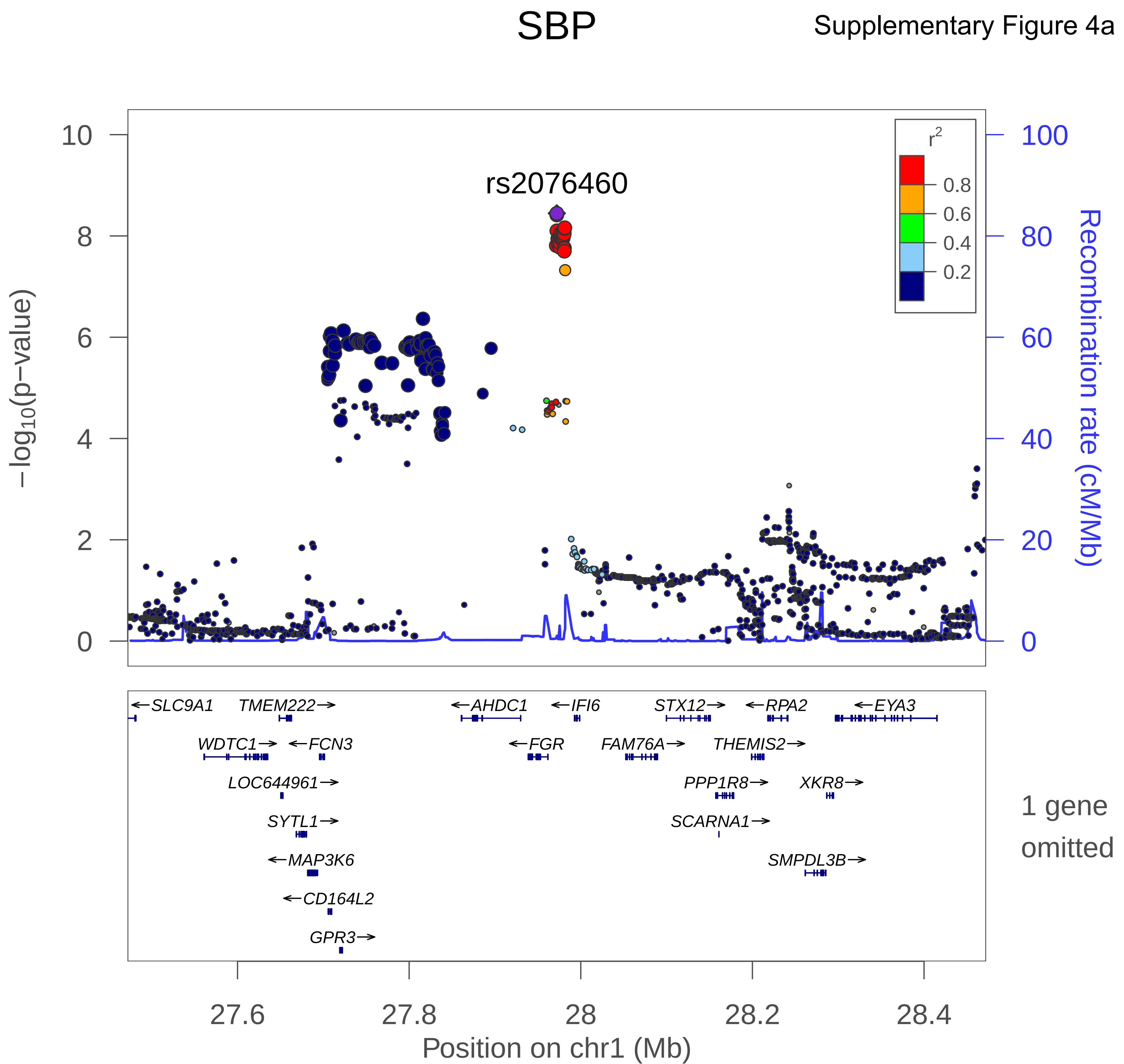

SBP
Supplementary Figure 4a
rs2076460
r2
0.8
0.6
0.4
0.2
10
8
6
4
2
0
−log10(p−value)
100
80
60
40
20
0
Recombination rate (cM/Mb)
SLC9A1
TMEM222
AHDC1
IFI6
STX12
RPA2
EYA3
WDTC1
FCN3
FGR
FAM76A
THEMIS2
LOC644961
PPP1R8
XKR8
SYTL1
SCARNA1
MAP3K6
SMPDL3B
CD164L2
GPR3
1 gene omitted
27.6
27.8
28
28.2
28.4
Position on chr1 (Mb)

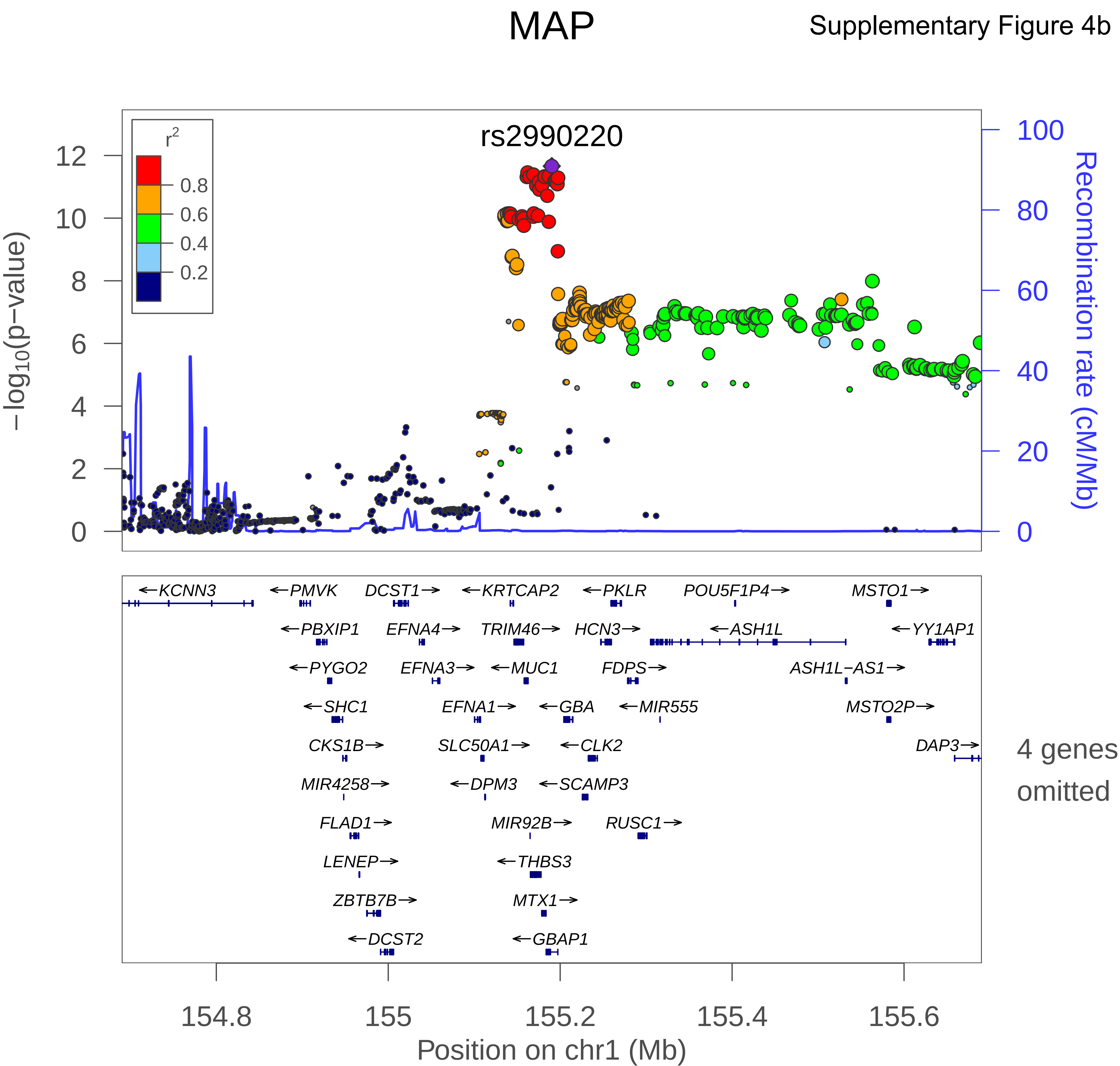
MAP
Supplementary Figure 4b
rs2990220
r2
0.8
0.6
0.4
0.2
12
10
8
6
4
2
0
−log10(p−value)
100
80
60
40
20
0
Recombination rate (cM/Mb)
KCNN3
PMVK
DCST1
KRTCAP2
PKLR
POU5F1P4
MSTO1
PBXIP1
EFNA4
TRIM46
HCN3
ASH1L
YY1AP1
PYGO2
EFNA3
MUC1
FDPS
ASH1L−AS1
SHC1
EFNA1
GBA
MIR555
MSTO2P
CKS1B
SLC50A1
CLK2
DAP3
MIR4258
DPM3
SCAMP3
FLAD1
MIR92B
RUSC1
LENEP
THBS3
ZBTB7B
MTX1
DCST2
GBAP1
4 genes omitted
154.8
155
155.2
155.4
155.6
Position on chr1 (Mb)

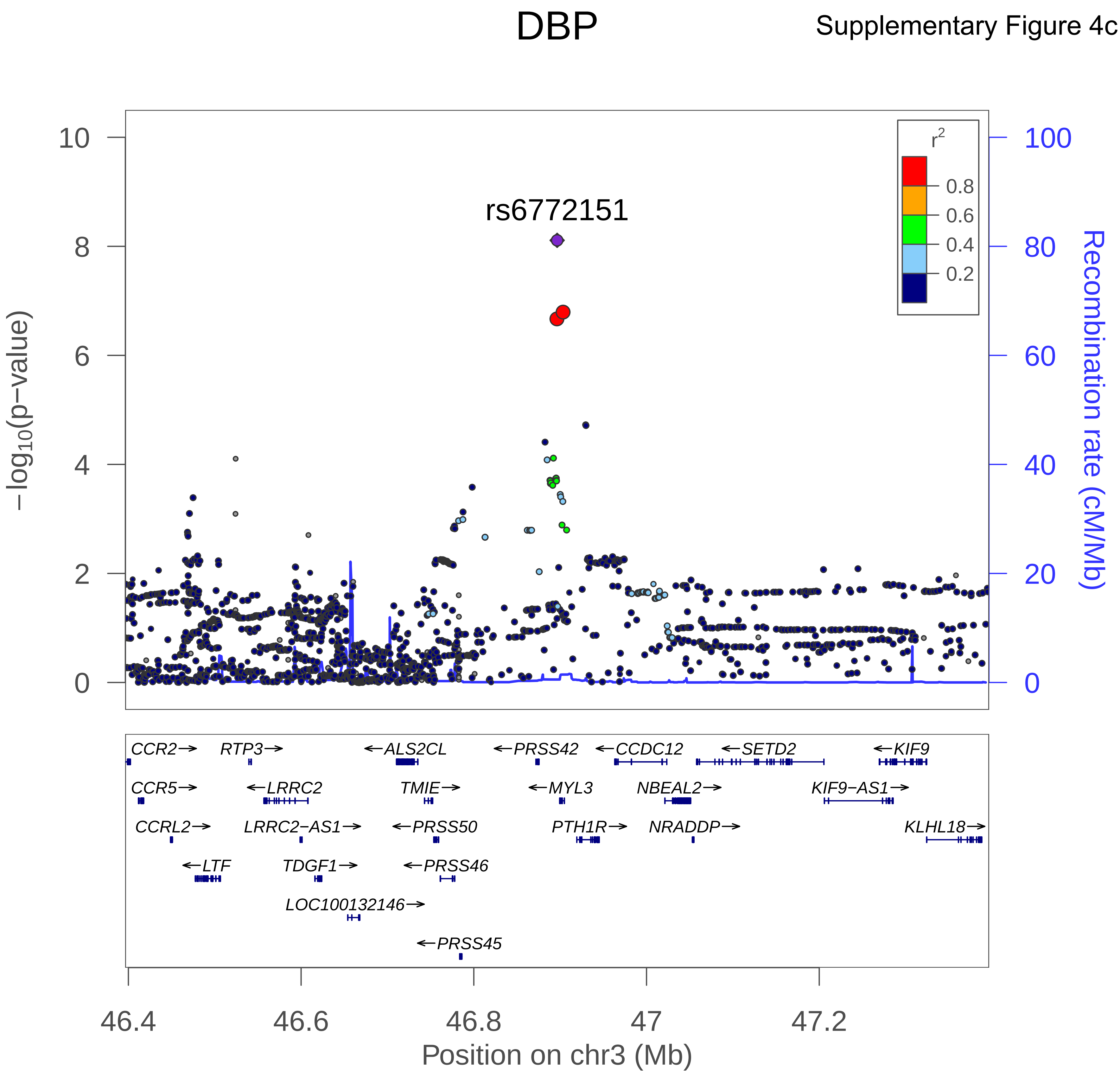
DBP
rs6772151
r^2
0.8
0.6
0.4
0.2
−log10(p−value)
Recombination rate (cM/Mb)
0
2
4
6
8
10
20
40
60
80
100
CCR2→
RTP3→
←ALS2CL
←PRSS42
←CCDC12
←SETD2
←KIF9
CCR5→
←LRRC2
TMIE→
←MYL3
NBEAL2→
KIF9−AS1→
CCRL2→
LRRC2−AS1→
←PRSS50
PTH1R→
NRADDP→
KLHL18→
←LTF
TDGF1→
←PRSS46
LOC100132146→
←PRSS45
46.4
46.6
46.8
47
47.2
Position on chr3 (Mb)

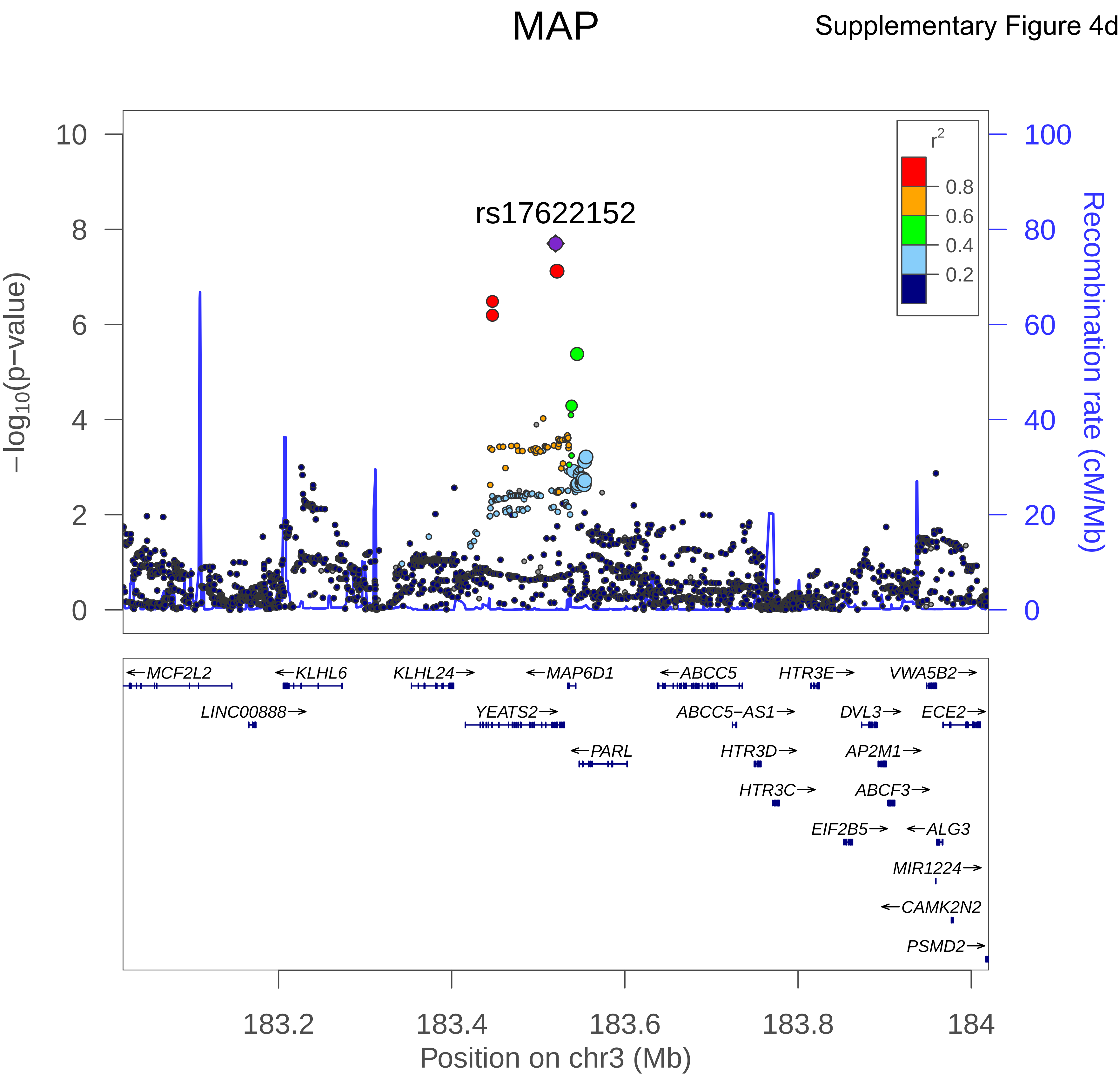
MAP
rs17622152
r^2
0.8
0.6
0.4
0.2
−log10(p−value)
Recombination rate (cM/Mb)
0
2
4
6
8
10
0
20
40
60
80
100
MCF2L2
KLHL6
KLHL24
MAP6D1
ABCC5
HTR3E
VWA5B2
LINC00888
YEATS2
ABCC5−AS1
DVL3
ECE2
PARL
HTR3D
AP2M1
HTR3C
ABCF3
EIF2B5
ALG3
MIR1224
CAMK2N2
PSMD2
183.2
183.4
183.6
183.8
184
Position on chr3 (Mb)

Supplementary Figure 4e

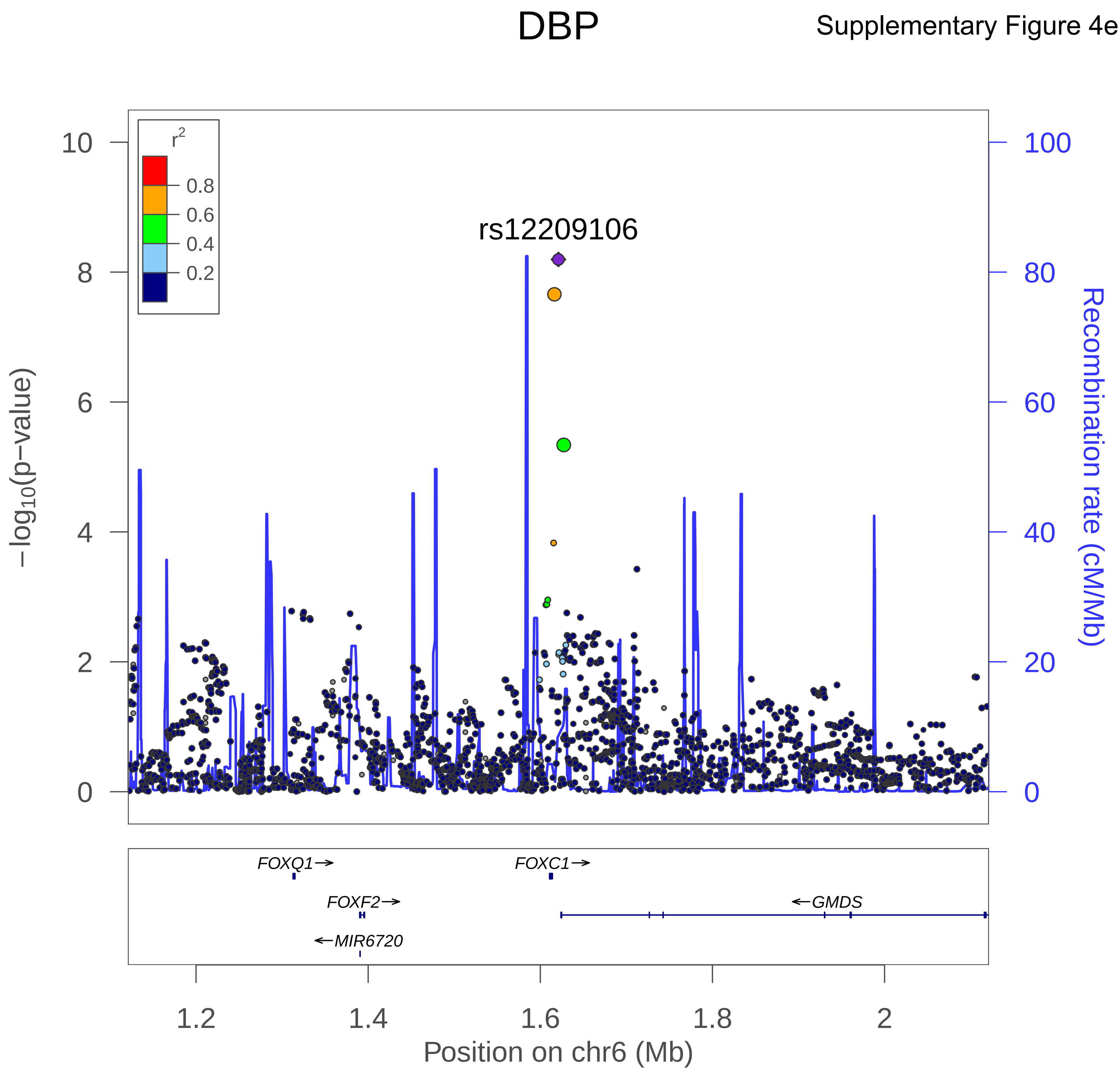

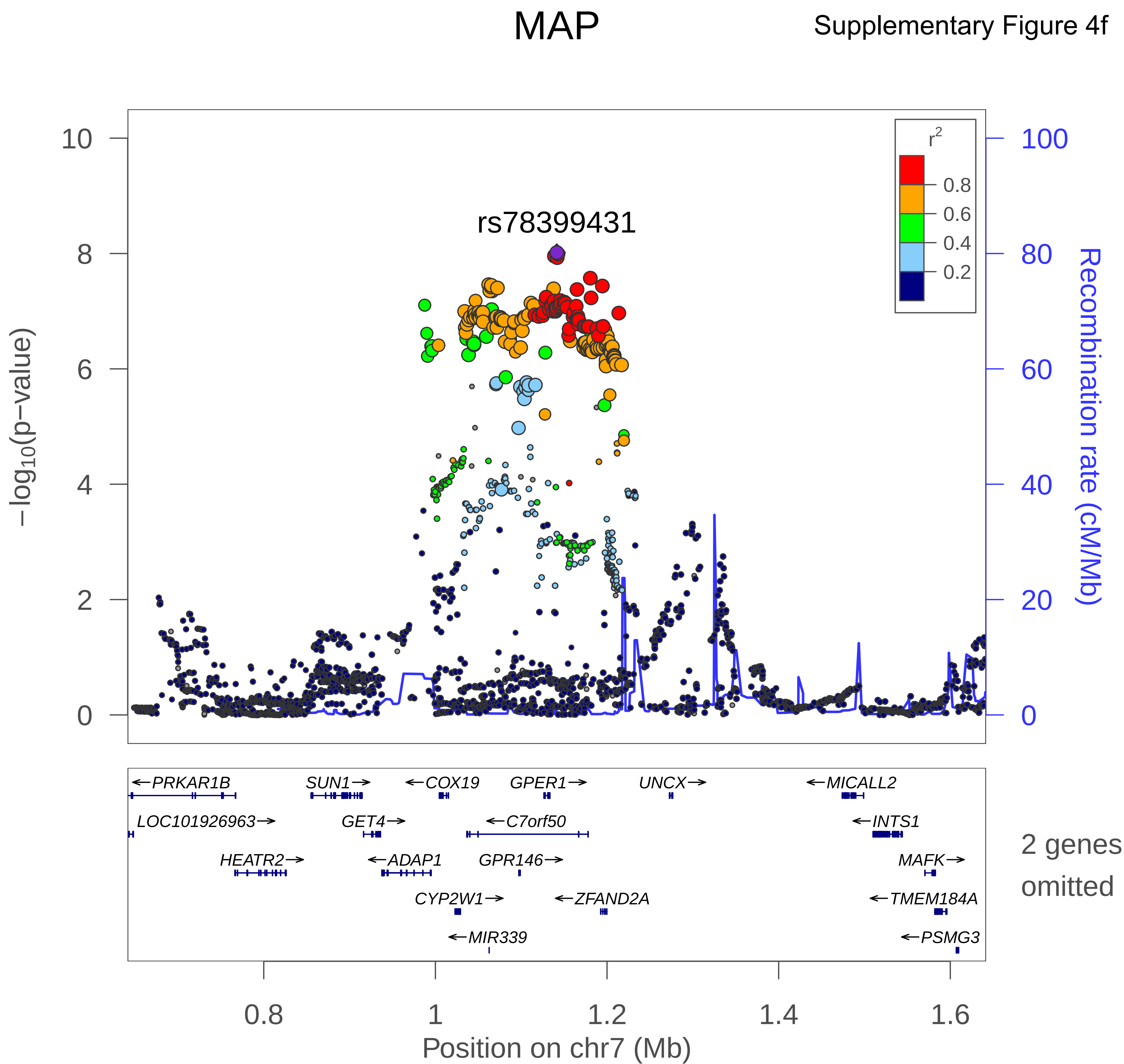

MAP
Supplementary Figure 4f
rs78399431
r2
0.8
0.6
0.4
0.2
−log10(p−value)
Recombination rate (cM/Mb)
0
2
4
6
8
10
20
40
60
80
100
PRKAR1B
SUN1
COX19
GPER1
UNCX
MICALL2
LOC101926963
GET4
C7orf50
INTS1
HEATR2
ADAP1
GPR146
MAFK
CYP2W1
ZFAND2A
TMEM184A
MIR339
PSMG3
2 genes omitted
0.8
1
1.2
1.4
1.6
Position on chr7 (Mb)

Supplementary Figure 4g

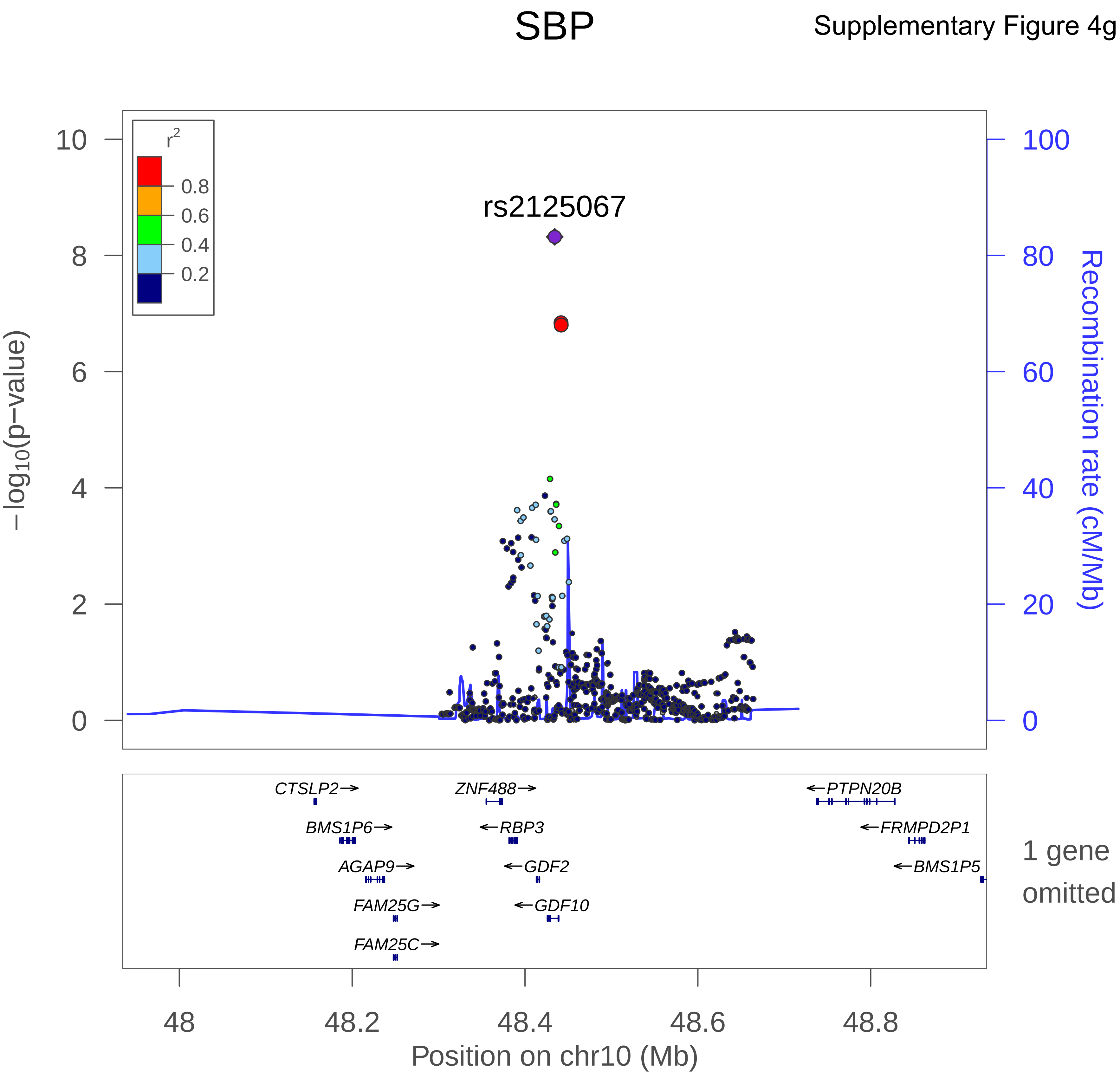

SBP

Supplementary Figure 4h

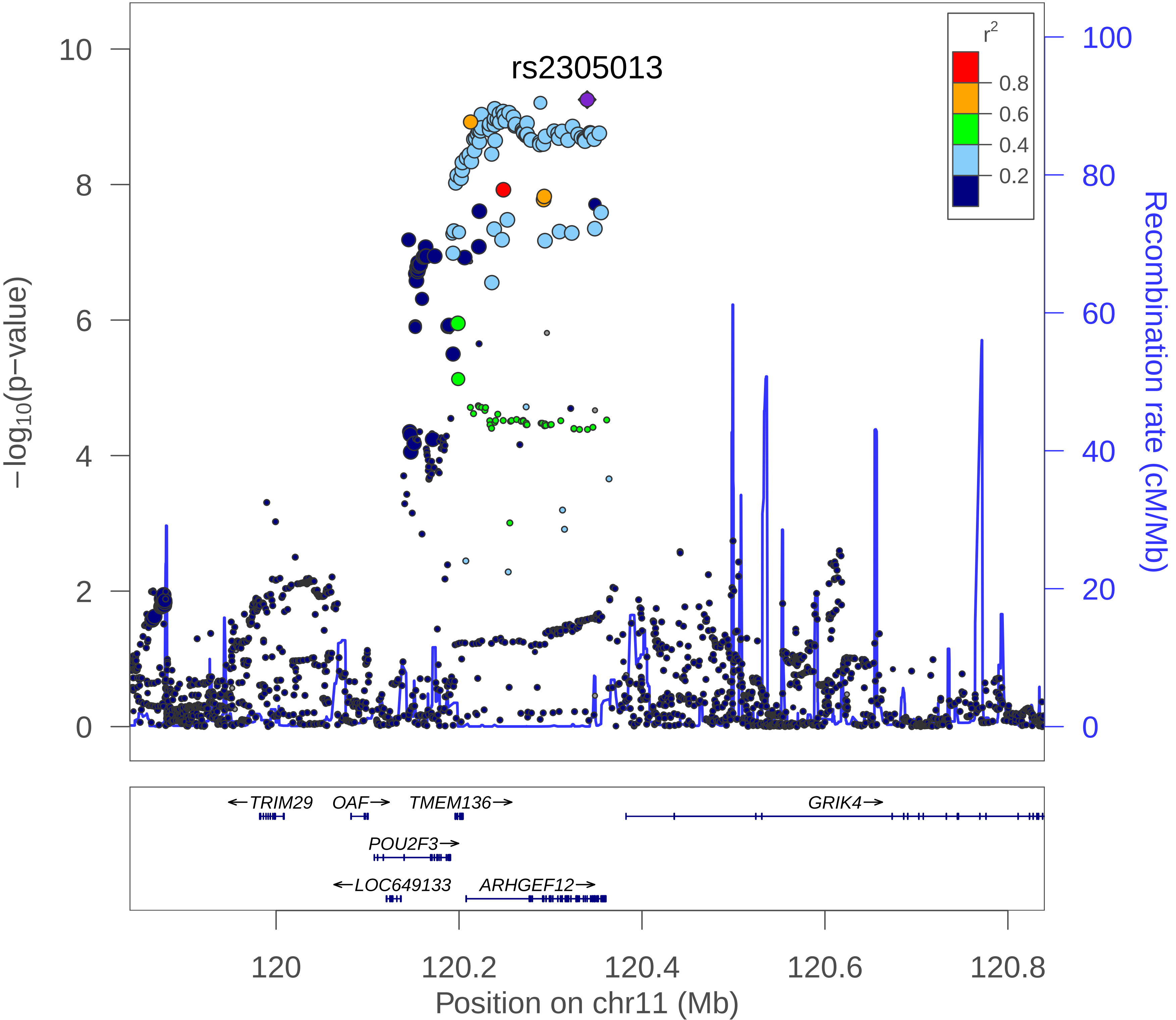

HT

Supplementary Figure 4i

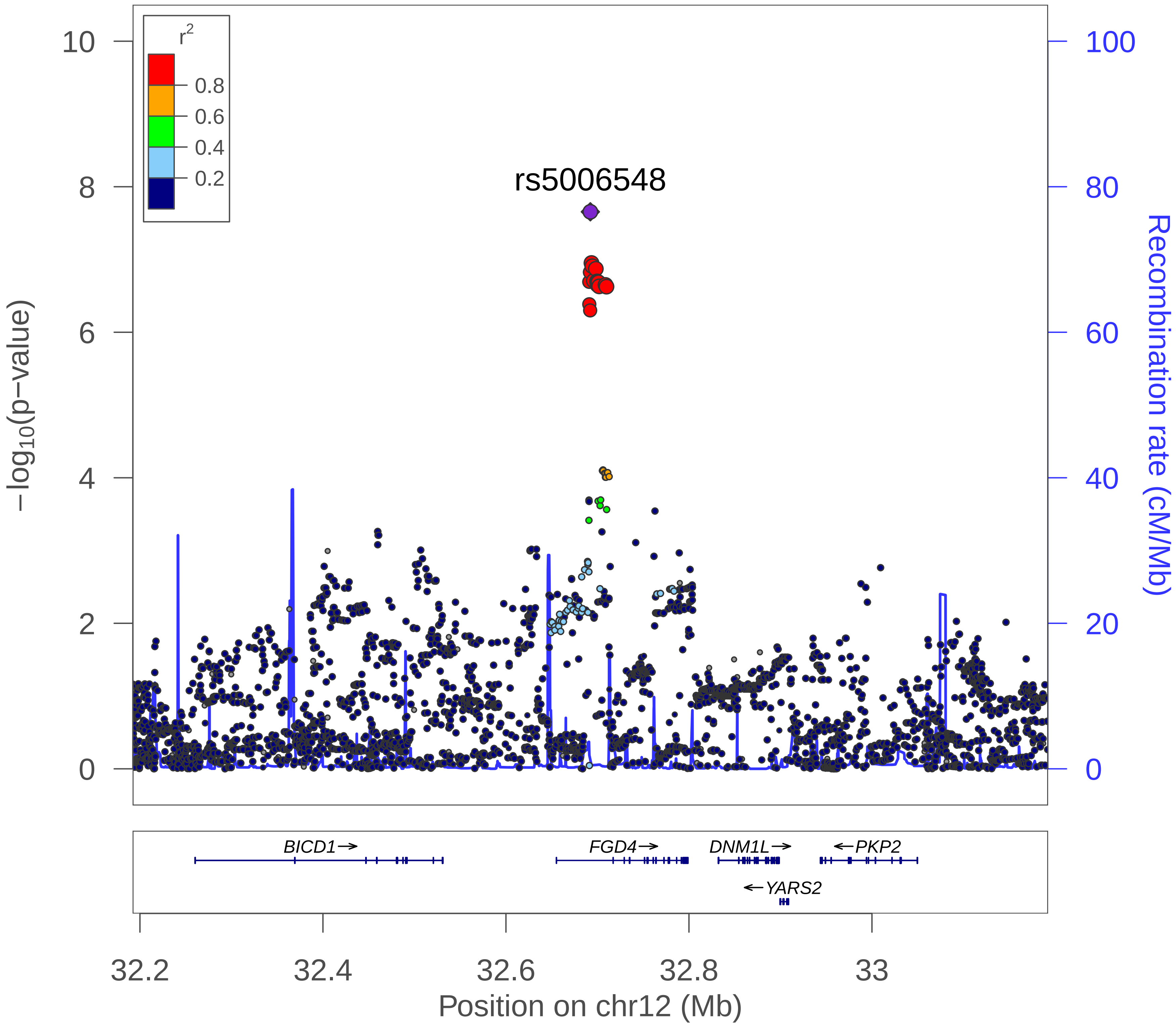

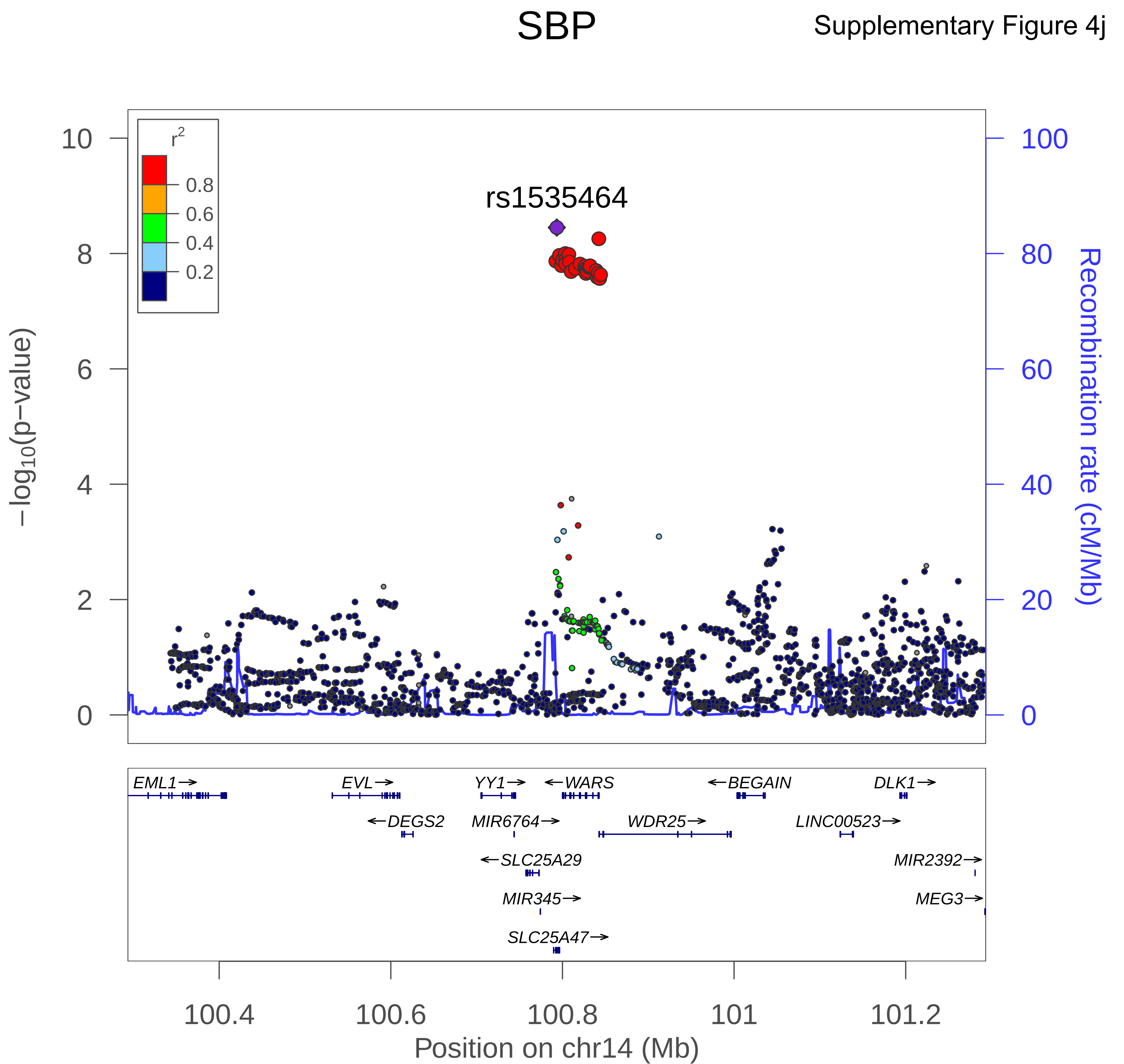
SBP
rs1535464
r2
0.8
0.6
0.4
0.2
10
8
6
4
2
0
−log10(p−value)
100
80
60
40
20
0
Recombination rate (cM/Mb)
EML1→
EVL→
YY1→
←WARS
←BEGAIN
DLK1→
←DEGS2
MIR6764→
WDR25→
LINC00523→
←SLC25A29
MIR2392→
MIR345→
MEG3→
SLC25A47→
100.4
100.6
100.8
101
101.2
Position on chr14 (Mb)

Supplementary Figure 4k

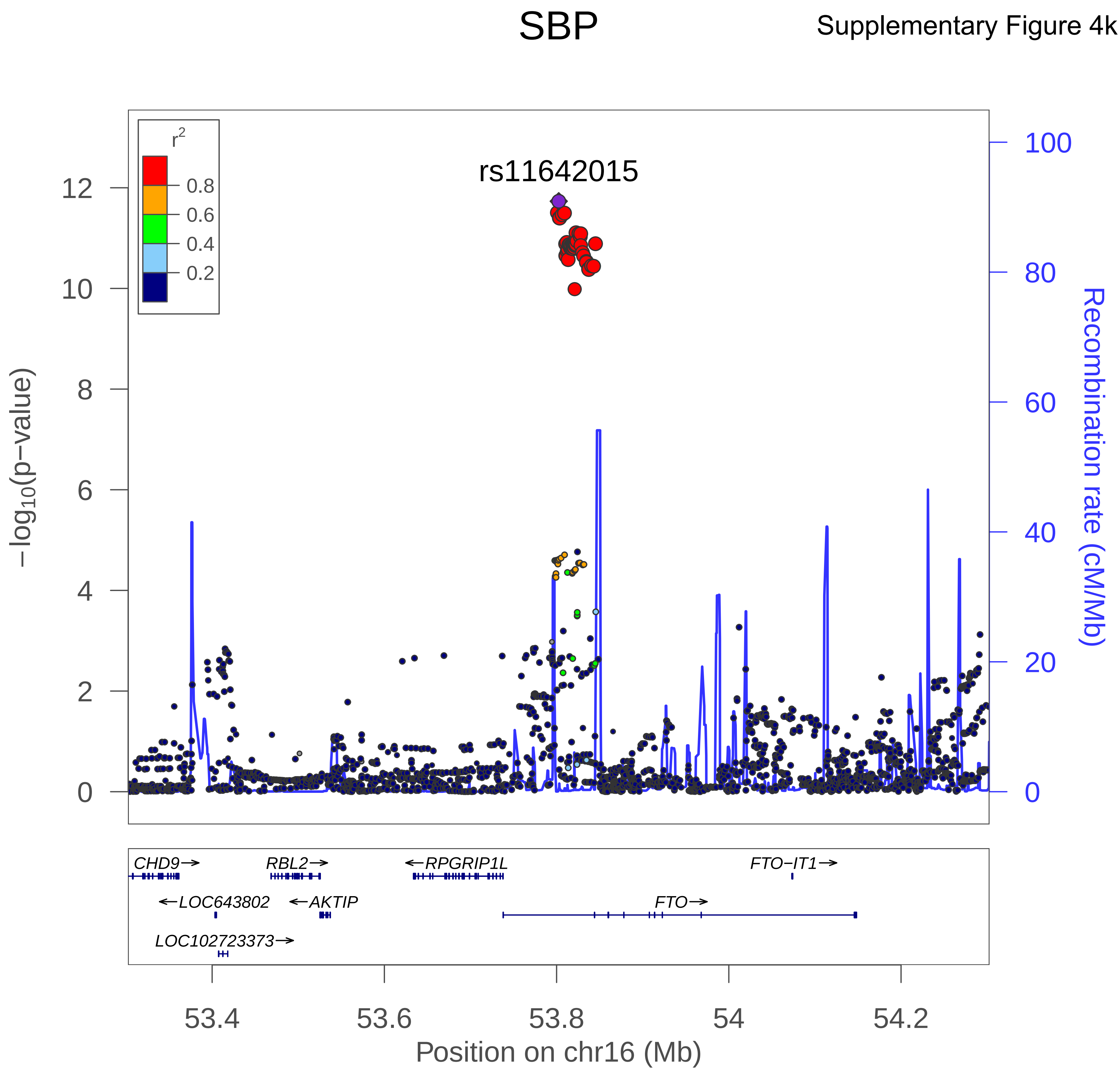

SBP

Supplementary Figure 4I

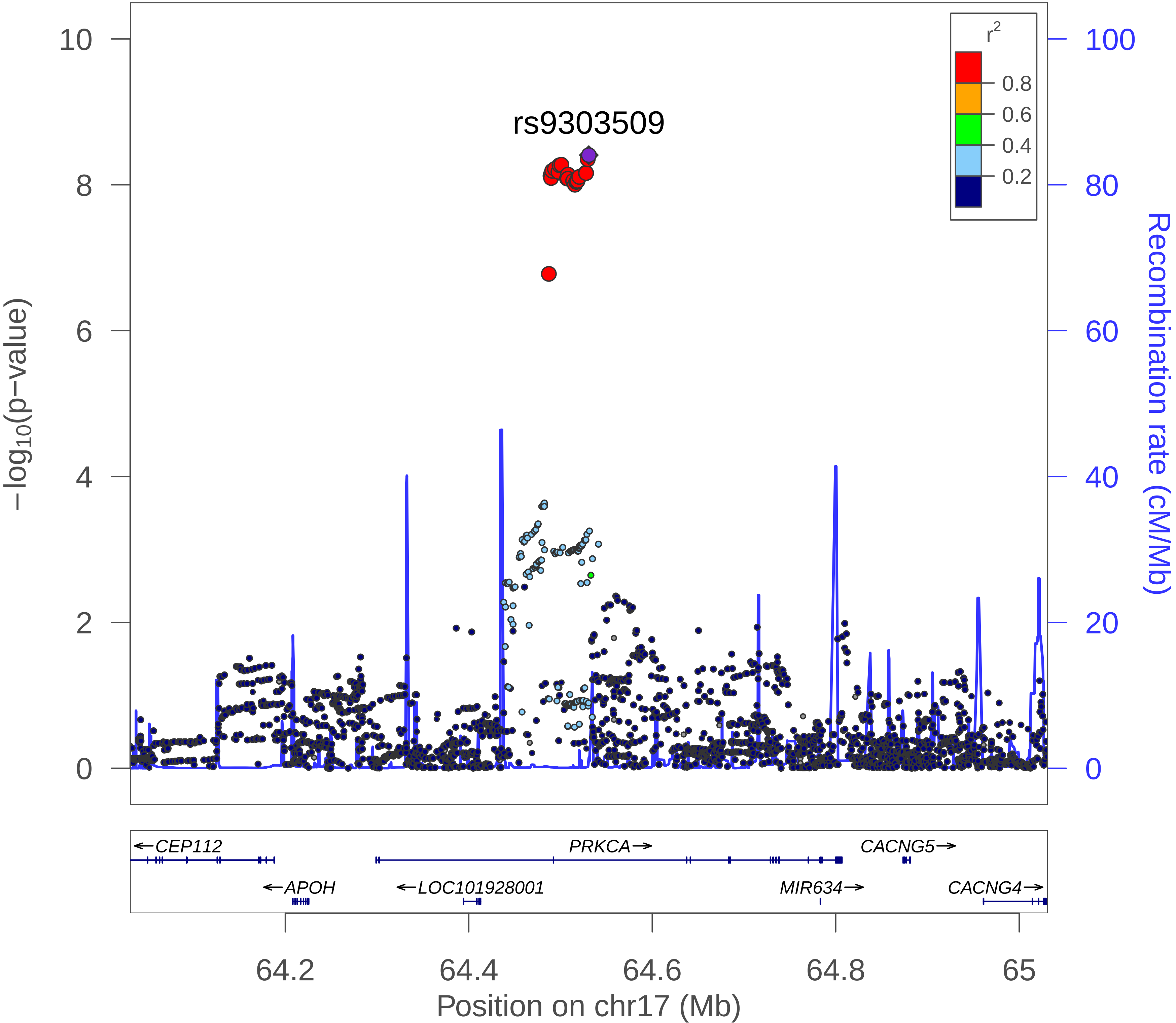

HT

Supplementary Figure 4m

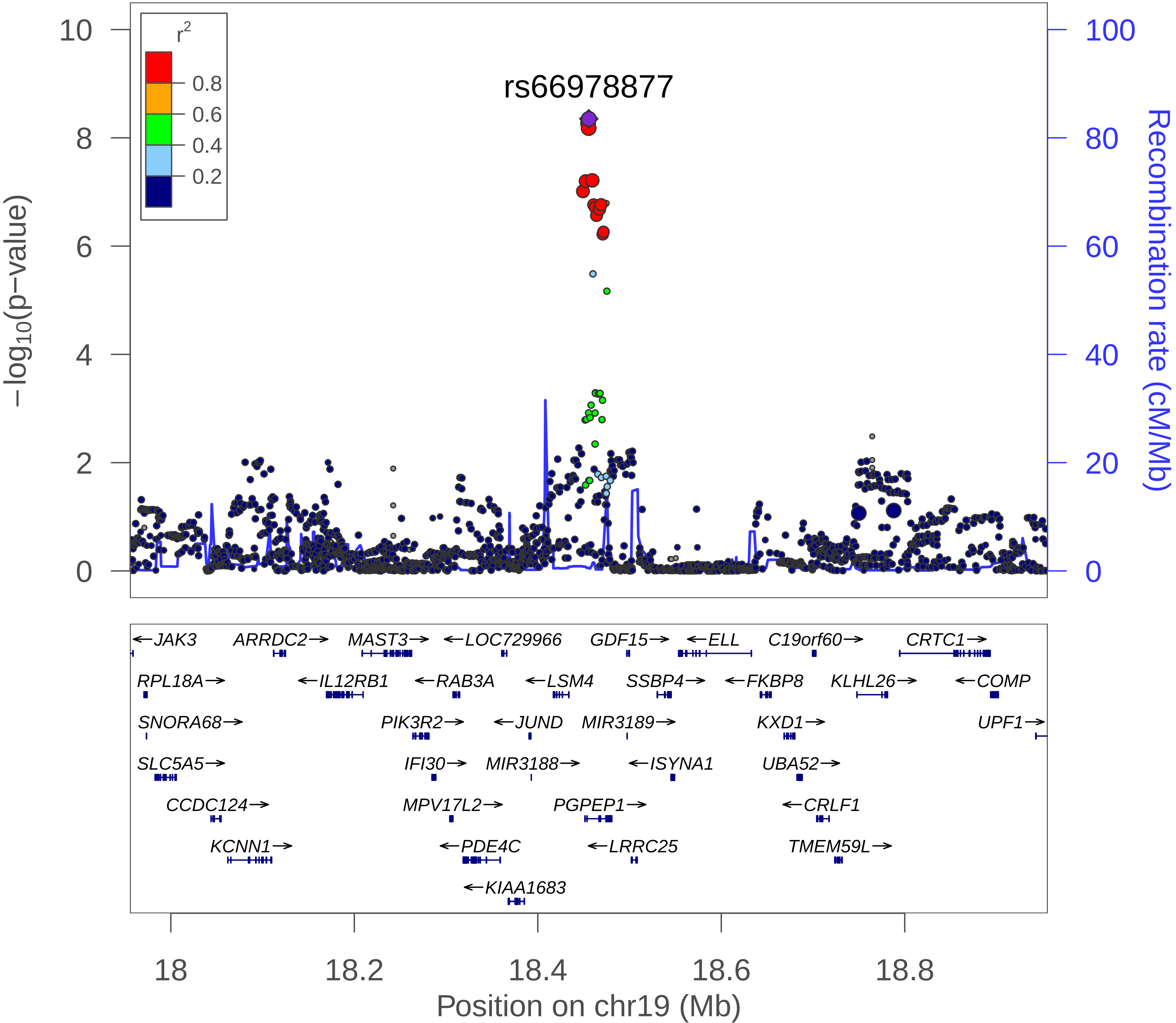

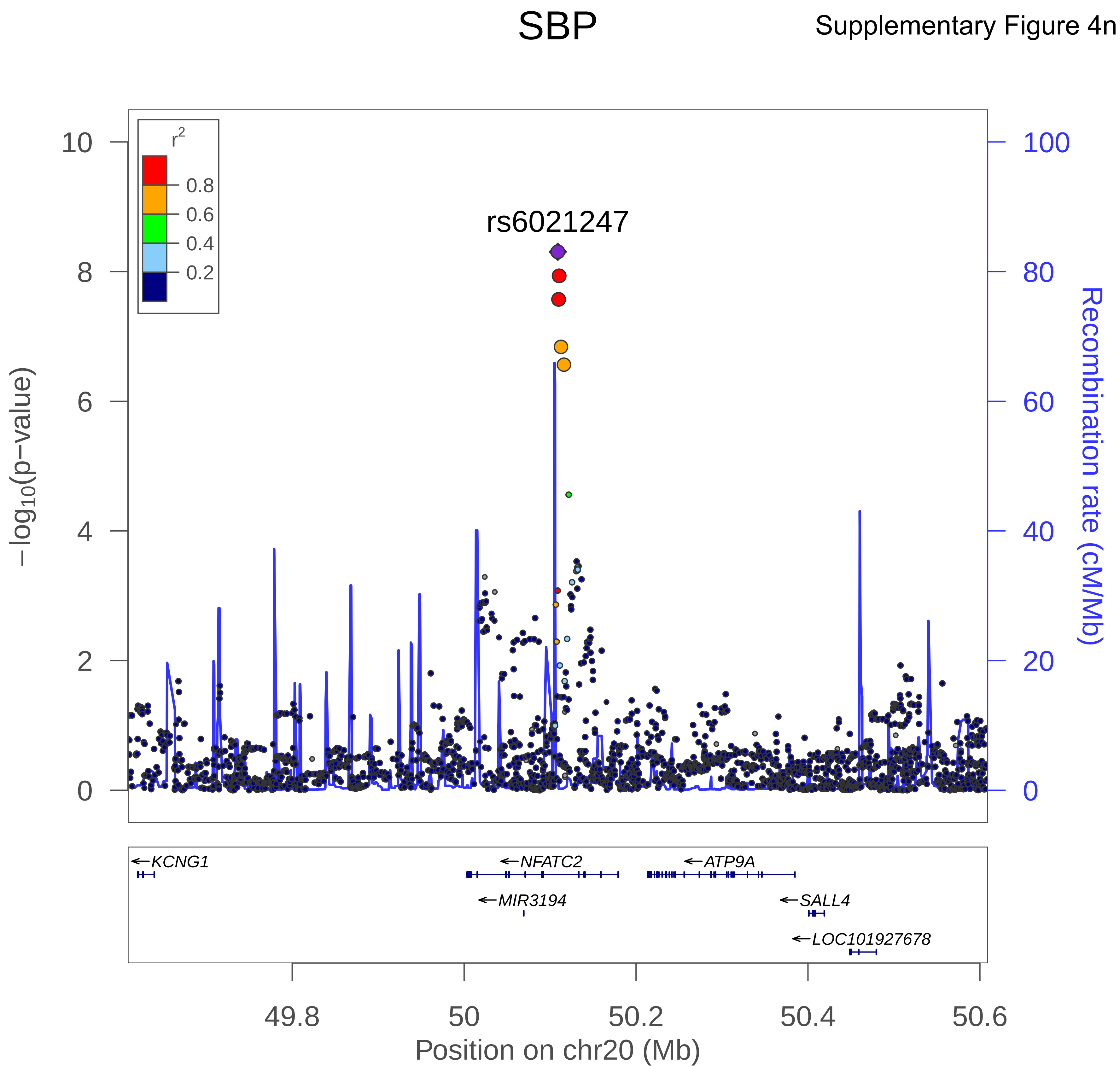
SBP
rs6021247
r2
0.8
0.6
0.4
0.2
−log10(p−value)
Recombination rate (cM/Mb)
10
8
6
4
2
0
100
80
60
40
20
0
KCNG1
NFATC2
ATP9A
MIR3194
SALL4
LOC101927678
49.8
50
50.2
50.4
50.6
Position on chr20 (Mb)

Supplementary Figure 4o

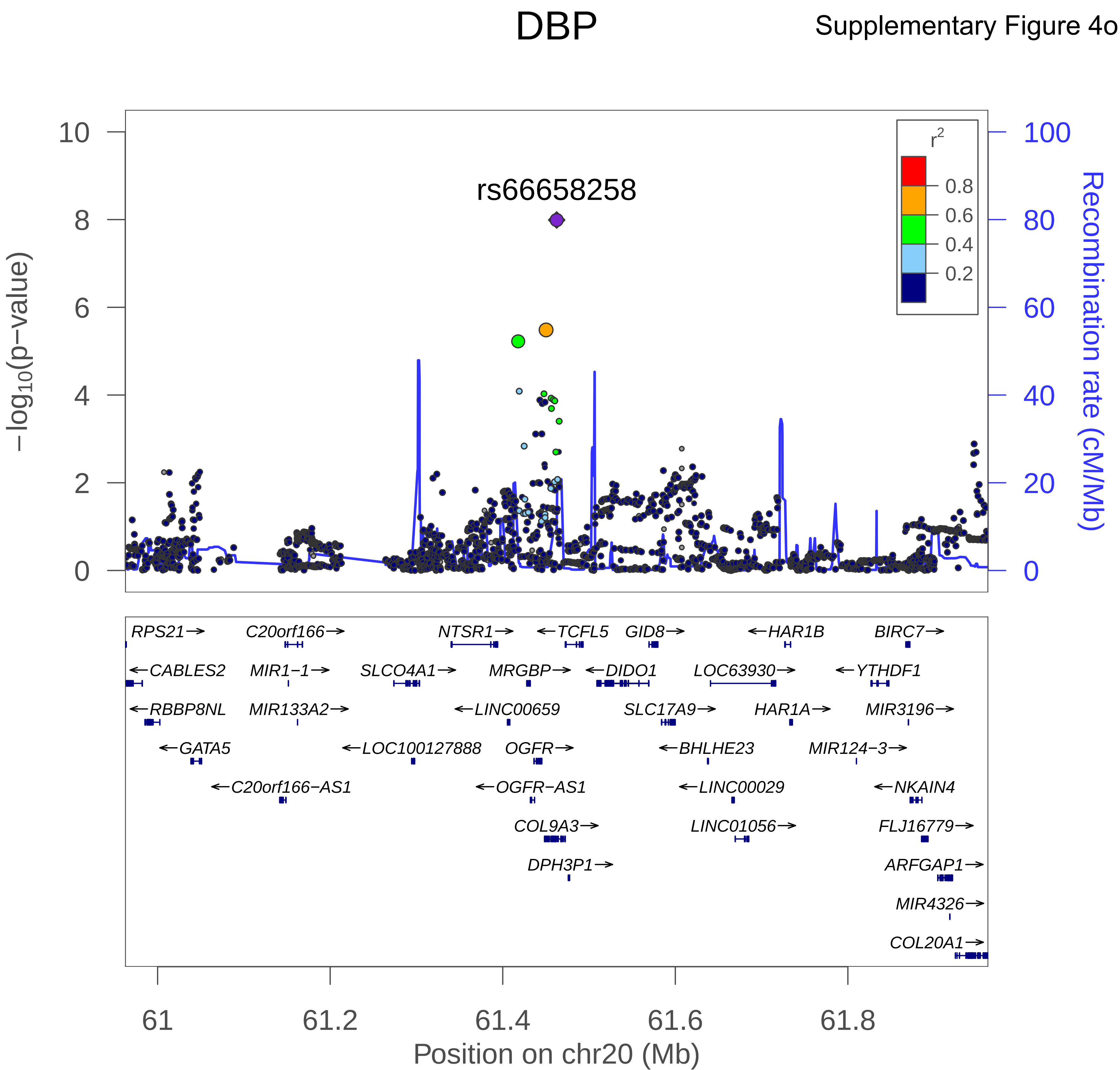

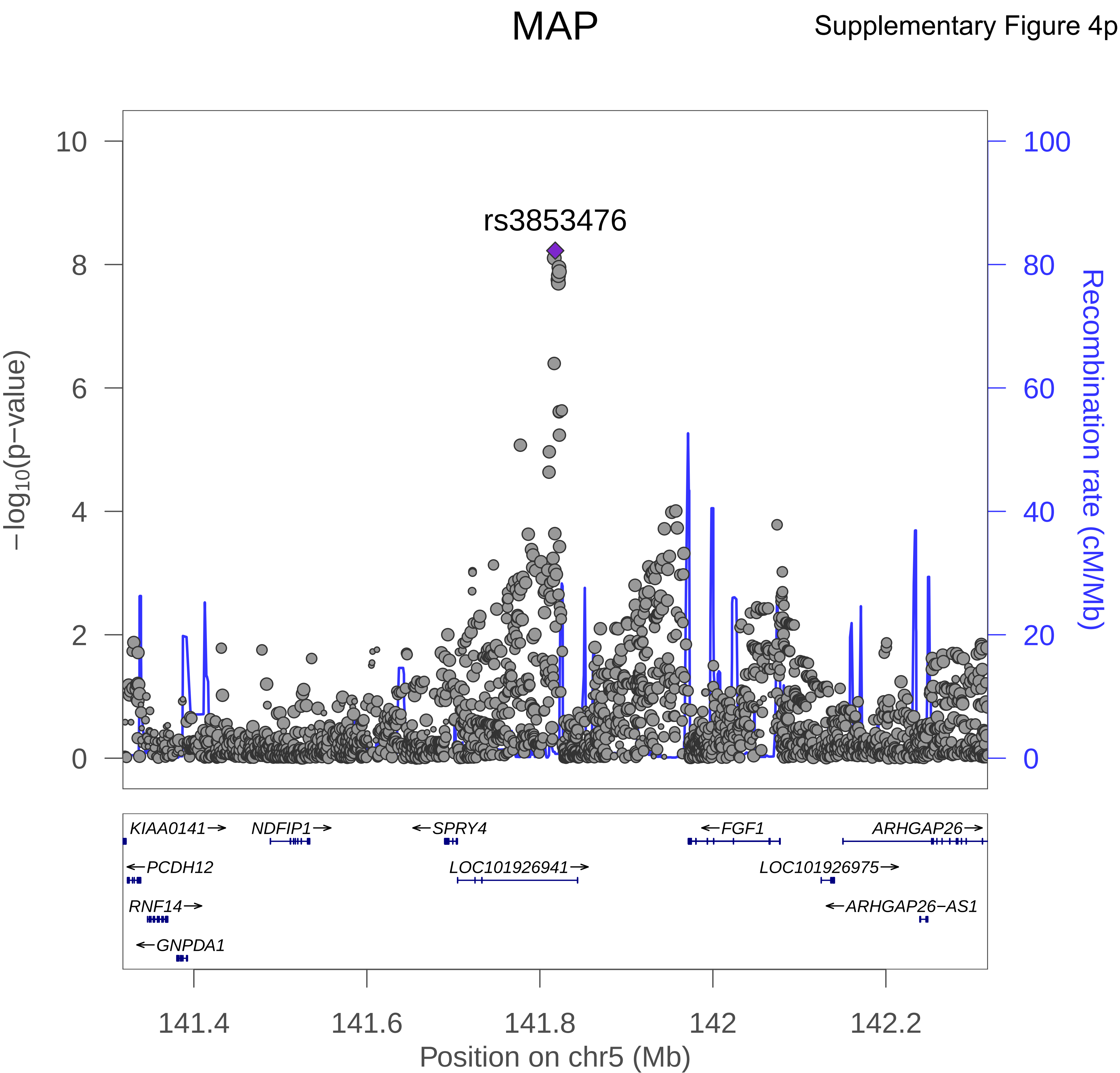
MAP
rs3853476
−log10(p−value)
Recombination rate (cM/Mb)
10
8
6
4
2
0
100
80
60
40
20
0
KIAA0141→
NDFIP1→
←SPRY4
←FGF1
ARHGAP26→
←PCDH12
LOC101926941→
LOC101926975→
RNF14→
←ARHGAP26−AS1
←GNPDA1
141.4
141.6
141.8
142
142.2
Position on chr5 (Mb)

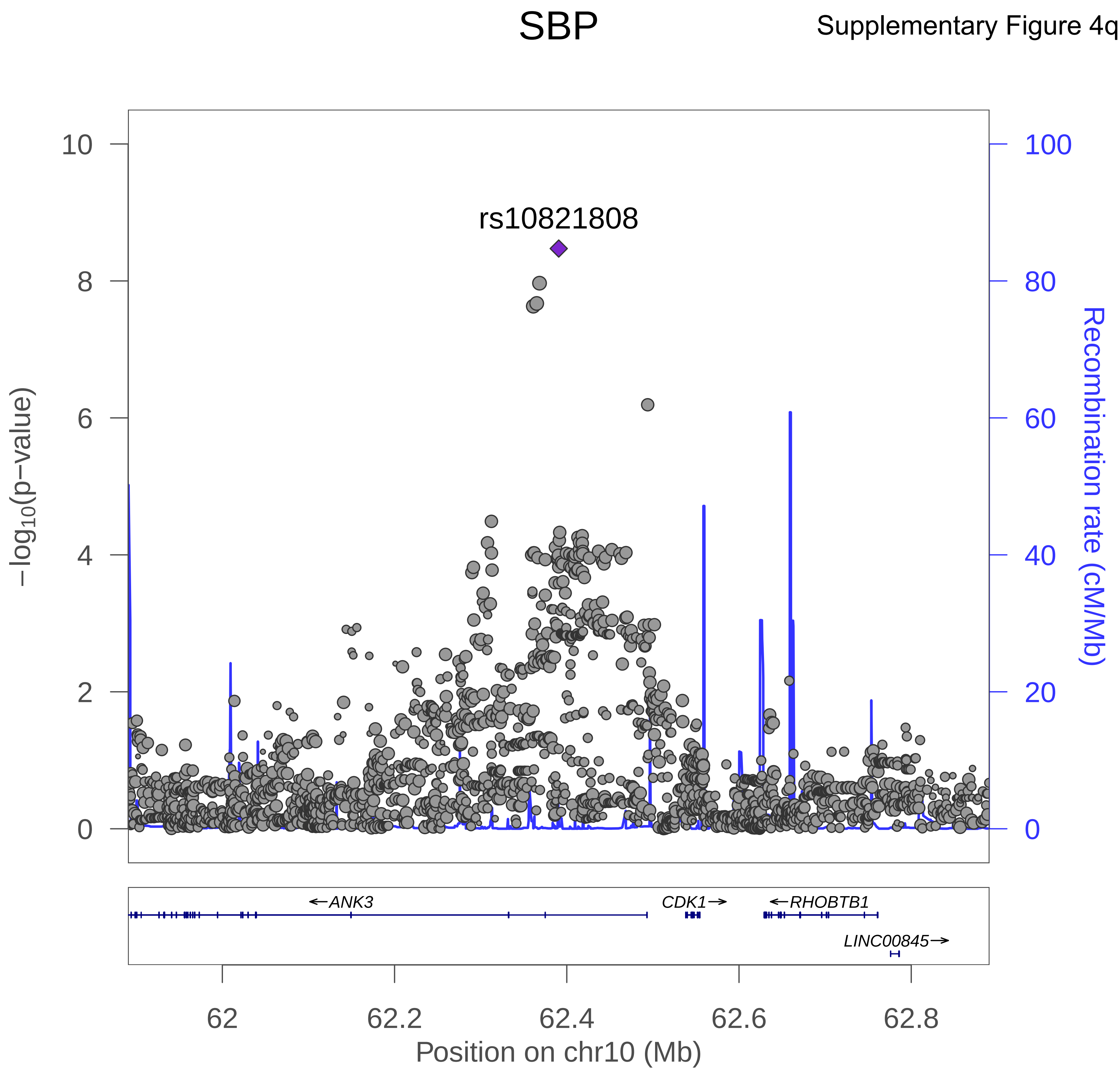
SBP
rs10821808
−log10(p−value)
Recombination rate (cM/Mb)
10
8
6
4
2
0
100
80
60
40
20
0
ANK3
CDK1
RHOBTB1
LINC00845
62
62.2
62.4
62.6
62.8
Position on chr10 (Mb)

Supplementary Figure 4r

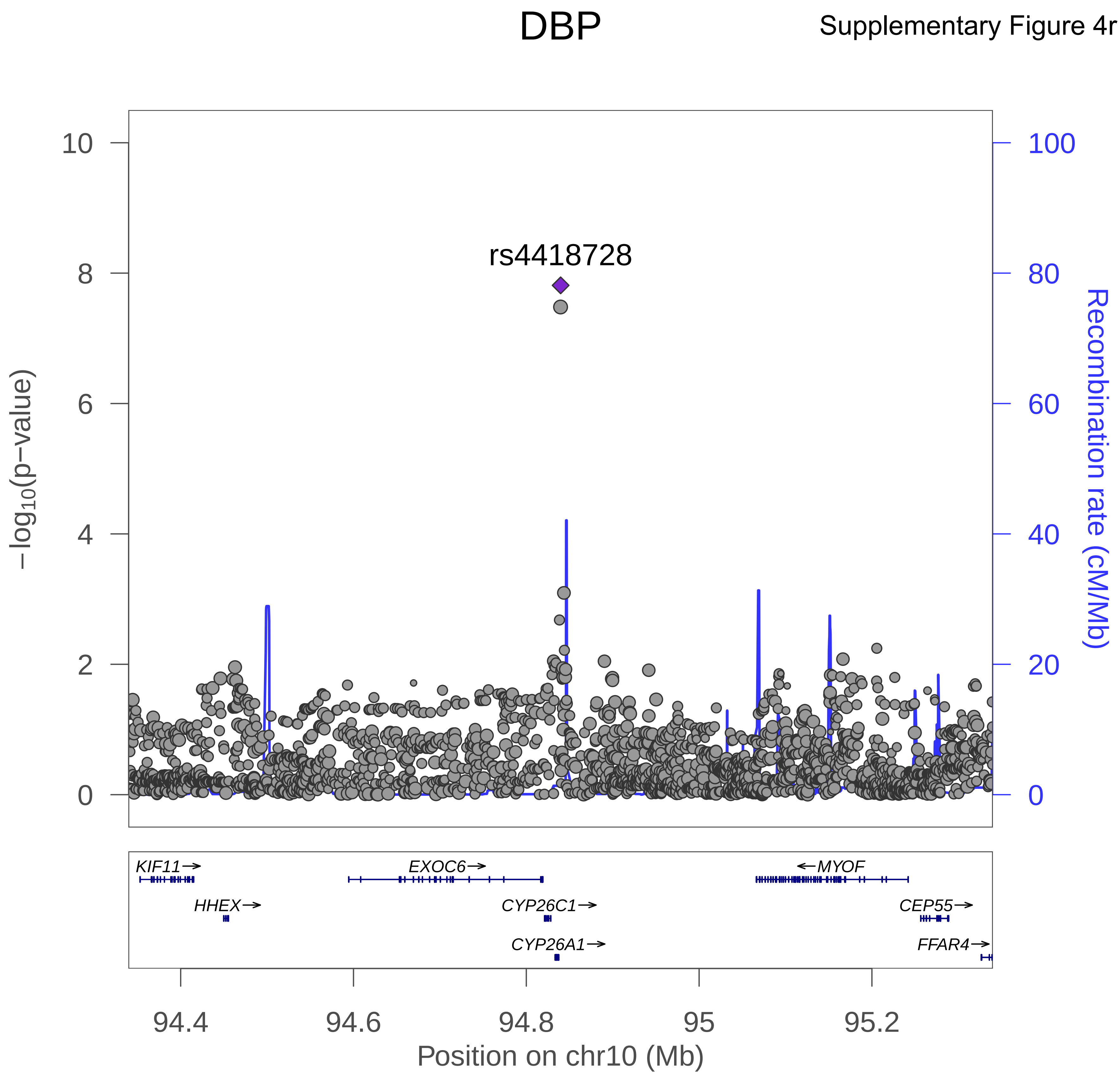

Supplementary Figure 4s

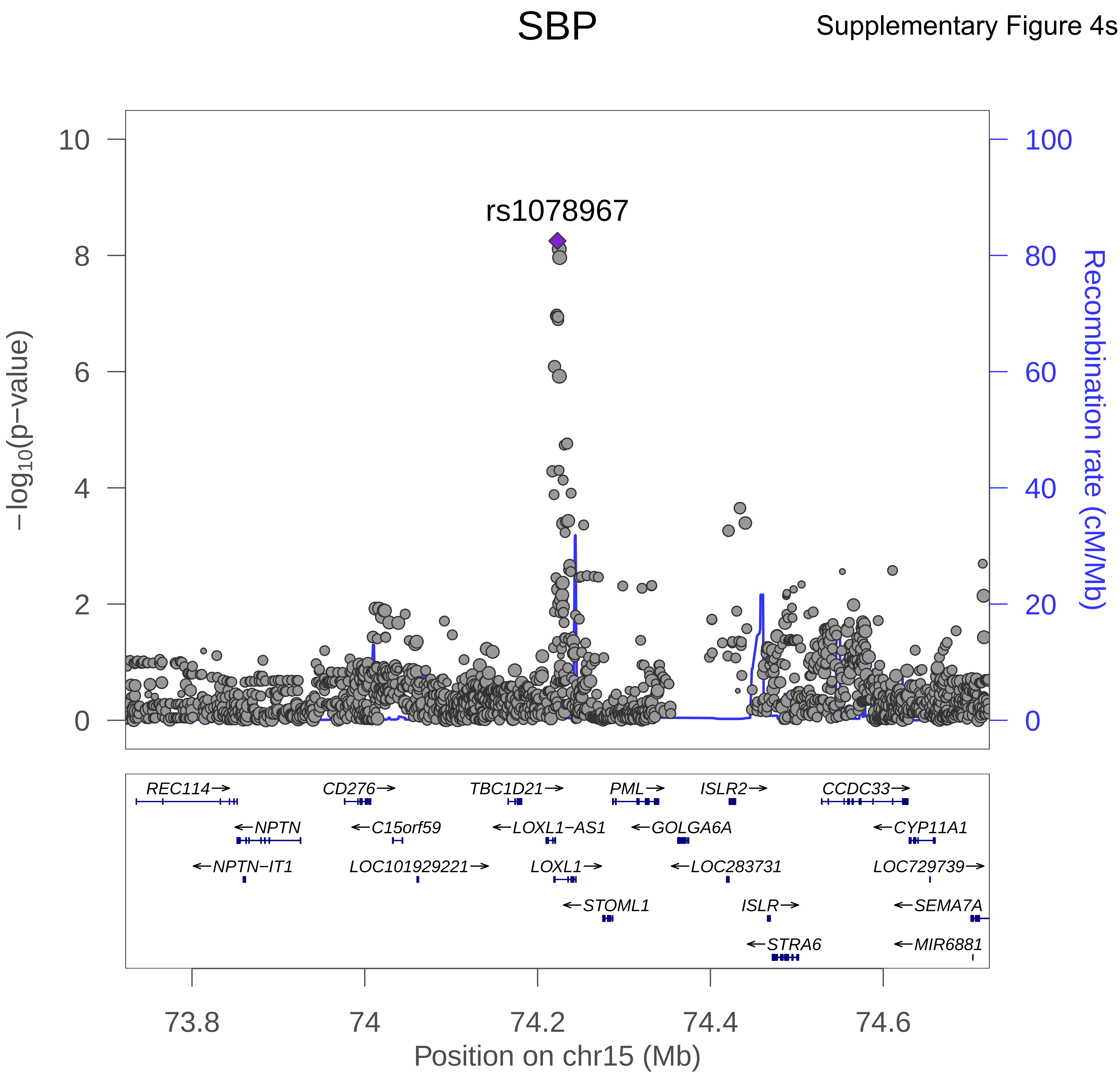

Supplementary Figure 4. Regional association plots of 19 newly identified loci. Point sizes are proportional to the tested sample size in two-stage East Asian meta-analyses except for four regions near *SPRY4-AS1* (rs3853476) on chromosome 5 (**p**), *ANK3* (rs10821808) (**q**), *CYP26A1* (rs4418728) (**r**) on chromosome 10 and *LOXL1* (rs1078967) (**s**), where transethnic meta-analysis was carried out in up to 289,038 individuals. In each panel, the sentinel SNP is represented by a purple diamond with the joint analysis (combined *P*). The correlation (r^2) of the sentinel SNP to other SNPs at the locus is shown on a scale from minimal (dark blue) to maximal (red) for the loci identified by East Asian meta-analyses, whereas no correlation is shown for the three loci identified by transethnic meta-analysis. Superimposed are gene locations (below the plots) and recombination rate (blue).

a

Haplotype class	SNP								Haplotype frequency		
	rs11065987	rs4646777	rs3742000	rs16941804	rs12422941	rs10850014	rs2301757	rs671	Japanese (n=2123)	CEU (n=93)	YRI (n=110)
H1	A	A	C	T	C	G	A	G	0.14	0.12	0.15
H2	A	G	C	T	C	G	A	G			0.02
H3 (Ancestral)	A	G	C	T	T	G	A	G			0.17
H4	A	G	C	T	T	G	C	G	0.38		0.04
H5 (East Asian)	A	G	C	T	T	G	C	A	0.29		
H6	A	G	T	T	T	A	A	G	0.19	0.44	0.18
H7 (European)	G	G	T	T	T	A	A	G		0.36	
H8	A	G	C	T	T	A	A	G			0.07
H9	A	G	C	C	T	A	A	G			0.29
H10	A	A	C	C	T	A	A	G		0.07	0.06

Ancestral allele is colored pink.

b

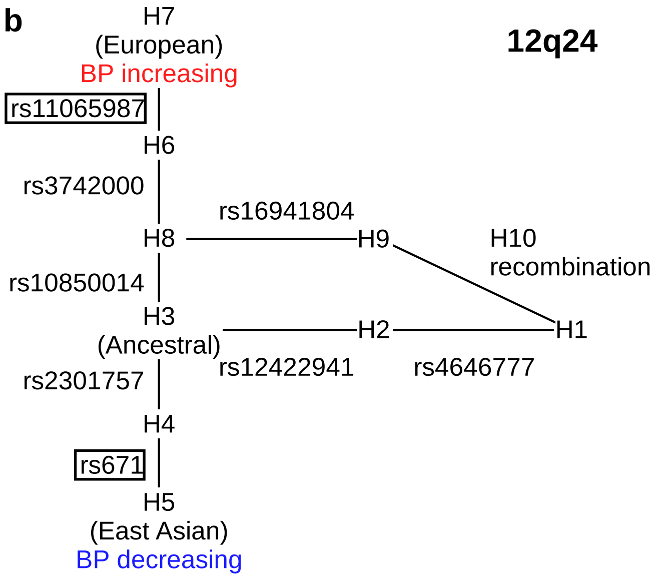

d

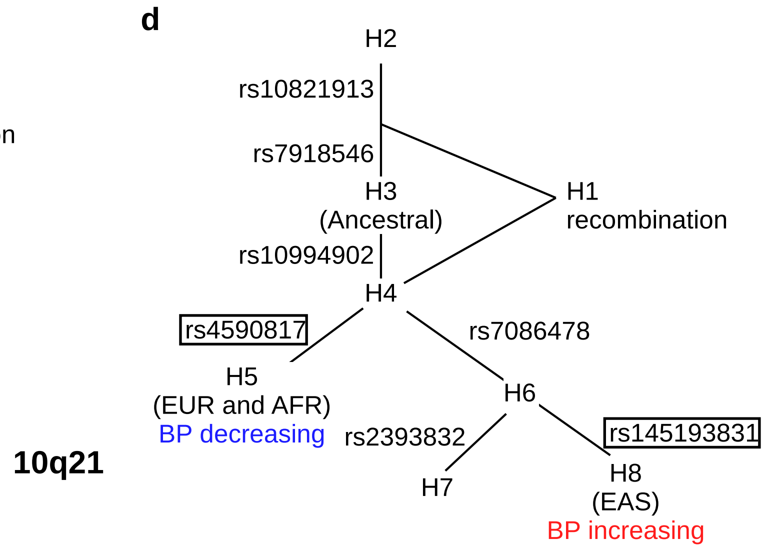

c

Haplotype class	SNP							Haplotype frequency		
	rs2393832	rs4590817	rs7918546	rs145193831	rs10821913	rs10994902	rs7086478	EAS (n=504)	EUR (n=503)	AFR (n=661)
H1	C	G	C	C	C	C	G			0.05
H2	C	G	C	C	T	A	G	0.17	0.55	0.10
H3 (Ancestral)	C	G	T	C	C	A	G	0.18	0.07	0.19
H4	C	G	T	C	C	C	G			0.02
H5 (EUR and AFR)	C	C	T	C	C	C	G		0.15	0.19
H6	C	G	T	C	C	C	A	0.03	0.07	0.33
H7	T	G	T	C	C	C	A	0.56	0.15	0.11
H8 (EAS)	C	G	T	T	C	C	A	0.07		

Ancestral allele is colored in pink.

Supplementary Figure 5. Phylogenetic relation of ancestry-specific SNPs on 12q24 and 10q21 with ancestral haplotypes. For the clusters of SNPs located in the interval showing limited recombination across the 3 populations (Japanese or JPT, CEU and YRI), we chose representative SNPs of each cluster and inferred haplotypes [(**a**) for 12q24 and (**c**) for 10q21] and their phylogeny [(**b**) for 12q24 and (**d**) for 10q21]. On 12q24, the haplotype classes specific to East Asians (H5) and Europeans (H7) are found to have occurred distantly in the phylogenetic tree. H3 (ancestral haplotype) is present in YRI alone. Similarly, on 10q21, the haplotype classes specific to East Asians (H8) and Europeans (H5) are found to have occurred distantly in the phylogenetic tree. The results for 12q24 are drawn from the previous study (Kato *et al.* Nat Genet 2011; ng.834).

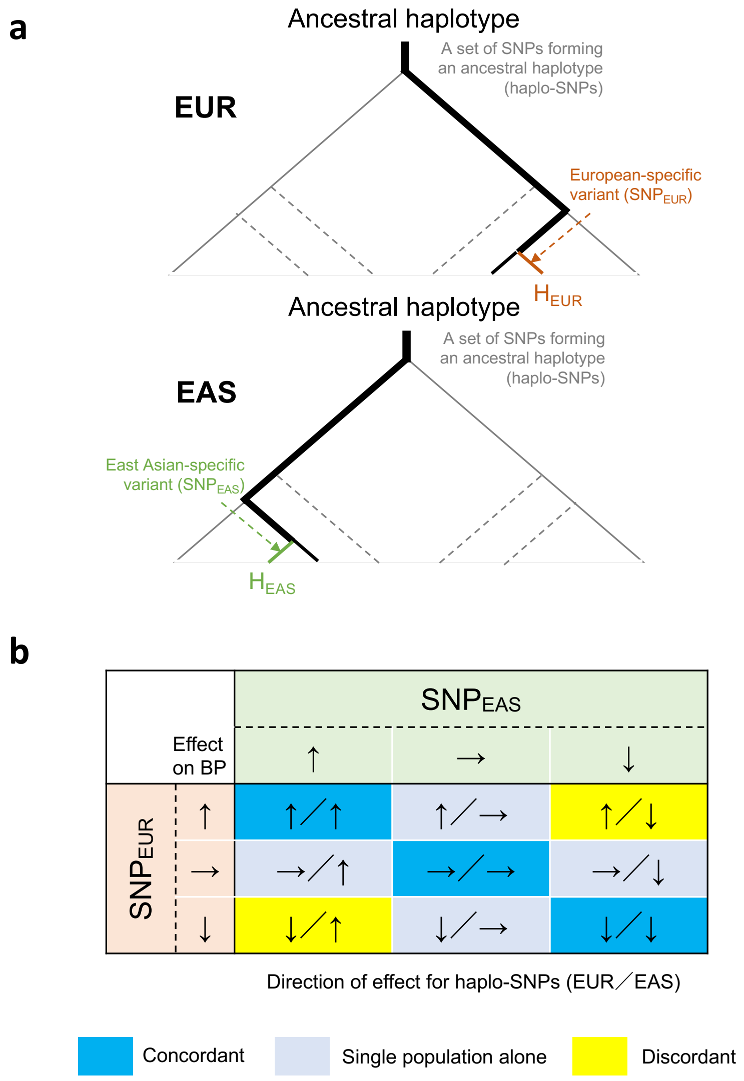

Supplementary Figure 6. Schematic explanation for interethnic comparability of genetic impact on blood pressure. (**a**) Simplified phylogenetic relationships of ancestry-specific sentinel SNPs with transethnic haplotypes detectable in Europeans (top) and East Asians (bottom). (**b**) Combination of ancestry-specific sentinel SNPs at individual loci and the resultant direction of effects for SNPs forming a shared haplotype at the locus. In this case, both SNP_{EUR} and SNP_{EAS} are assumed to have similarly (but independently) occurred on the common (shared) haplotype. However, the direction of effects needs to be inverted in the case that either of the ancestry-specific SNPs occurred on one shared haplotype consisting of derived alleles, whereas the remaining one occurred on another shared haplotype consisting of ancestral alleles; that is, the "side" of shared haplotype is flipped between SNP_{EUR} and SNP_{EAS}. Refer to **Supplementary Data 7** about the details of investigated loci.

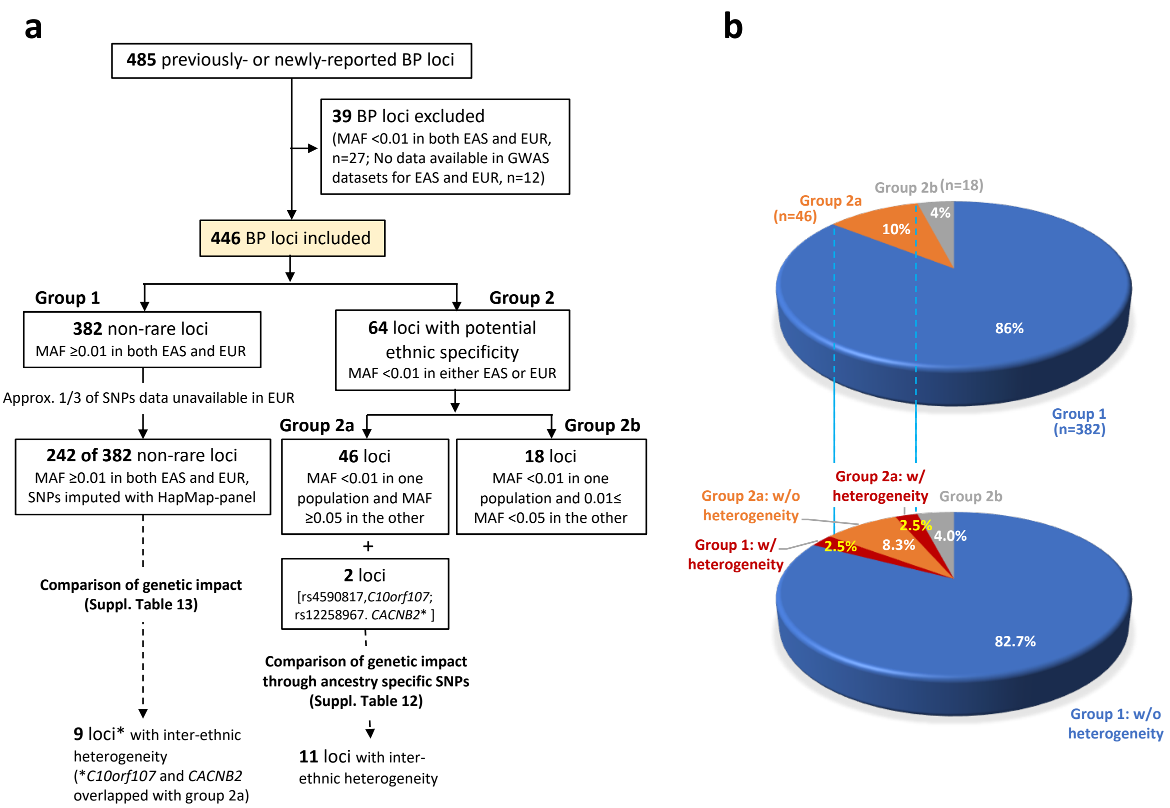

Supplementary Figure 7. Interethnic heterogeneity at blood pressure loci previously reported and newly identified. (**a**) Schematic explanation of analysis. A total of 446 loci were included in the present analysis and categorized into two groups—group 1 (382 non-rare loci; MAF ≥ 0.01 in both populations) and group 2 (64 loci with potential ethnic specificity). Group 2 was further classified into group 2a (46 loci with MAF < 0.01 in one population and MAF ≥ 0.05 in the other) and group 2b (18 loci with MAF < 0.01 in one population and 0.01 ≤ MAF < 0.05 in the other). See online methods about the details. (**b**) Proportion of the loci calculated in individual groups with and without interethnic heterogeneity.

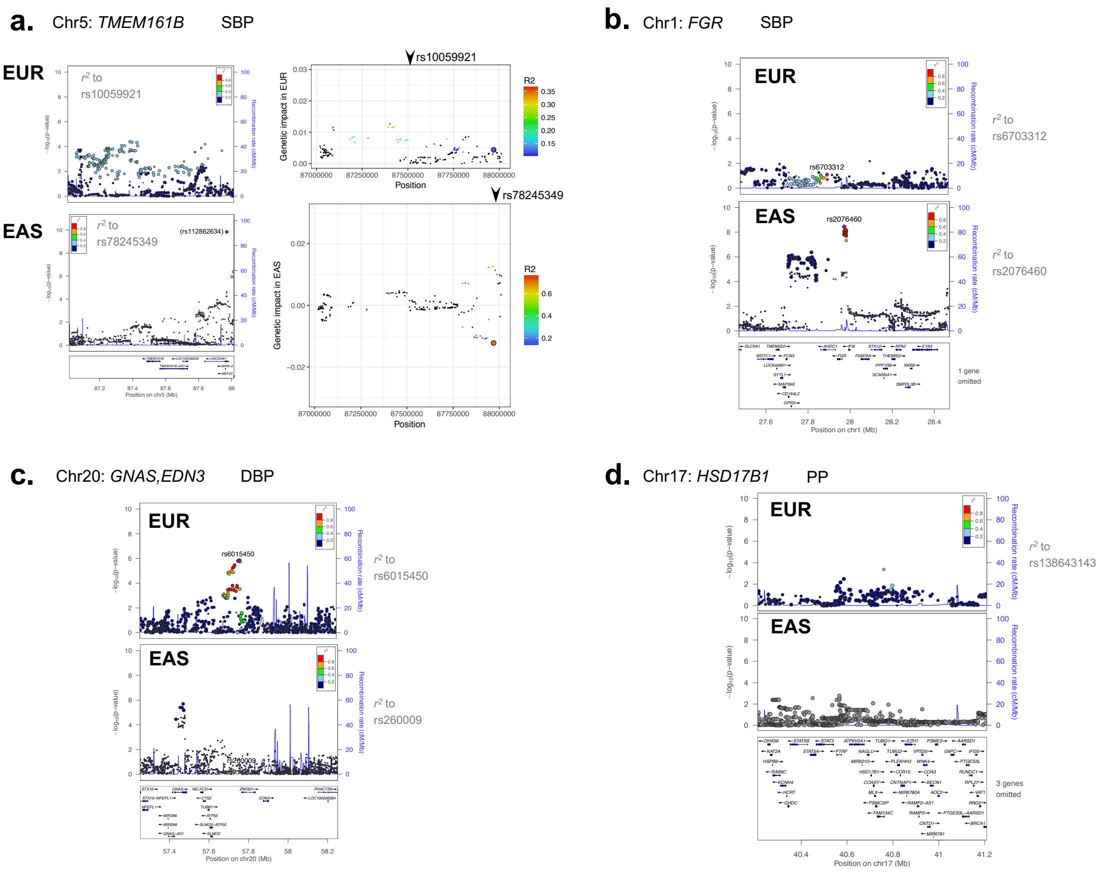

Supplementary Figure 8. Interethnic heterogeneity of genetic impact on and/or association with blood pressure at 4 selected loci involving ancestry-specific variants. (**a**) Regional plots for genetic association (left) and genetic impact (right), demonstrated separately for Europeans (EUR, top panel) and East Asians (EAS, bottom panel), on 5q14 near *TMEM161B*, where significant ($P < 1.5 \times 10^{-4}$) evidence for interethnic heterogeneity was observed (see **Supplementary Data 7**). Genetic impacts of individual SNPs are denoted such that those in Europeans are positive. Transethnic SNPs are plotted at the locus with bordered circles representing SNPs showing significant ($P < 1.5 \times 10^{-4}$) interethnic heterogeneity. Regional plots for genetic association are shown for 1p35 near *FGR* (**b**); 20q13 near *GNAS/EDN3* (**c**); and 17q21 near *HSD17B1* (**d**).

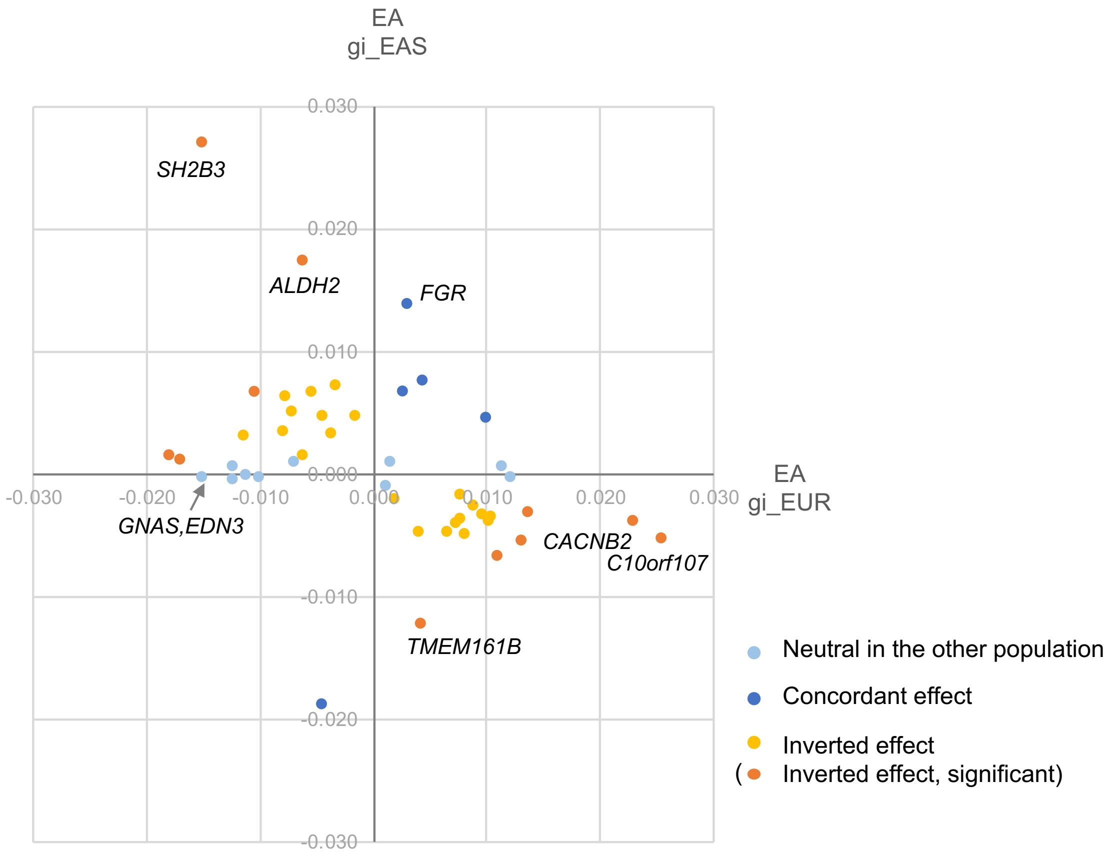

Supplementary Figure 9. Interethnic heterogeneity of genetic impact on blood pressure at ancestry-specific variant loci in group 2a (MAF < 0.01 in one population and MAF ≥ 0.05 in the other). In the plot, 47 of 48 SNP loci were included except for near *HSD17B1* on 17q21 (**Supplementary Fig. 8d**) and categorized into three classes; loci with mutually inverted genetic effects (colored in yellow or orange, n = 32), loci with distinct ancestry-specific variants showing the concordant directions of effect (colored in dark blue, n = 5) and loci with distinct ancestry-specific variants showing discordant genetic effects, one of which appeared to be almost "neutral" (i.e., genetic impact <|0.001| in either of the ethnic groups) (colored in light blue, n = 10). Interethnic differences in genetic impact at the haplo-SNP were considered to be significant at $P < 1.5 \times 10^{-4} \simeq 5 \times 10^{-8} \times$ [3 Gb/1 Mb]. gi: genetic impact.

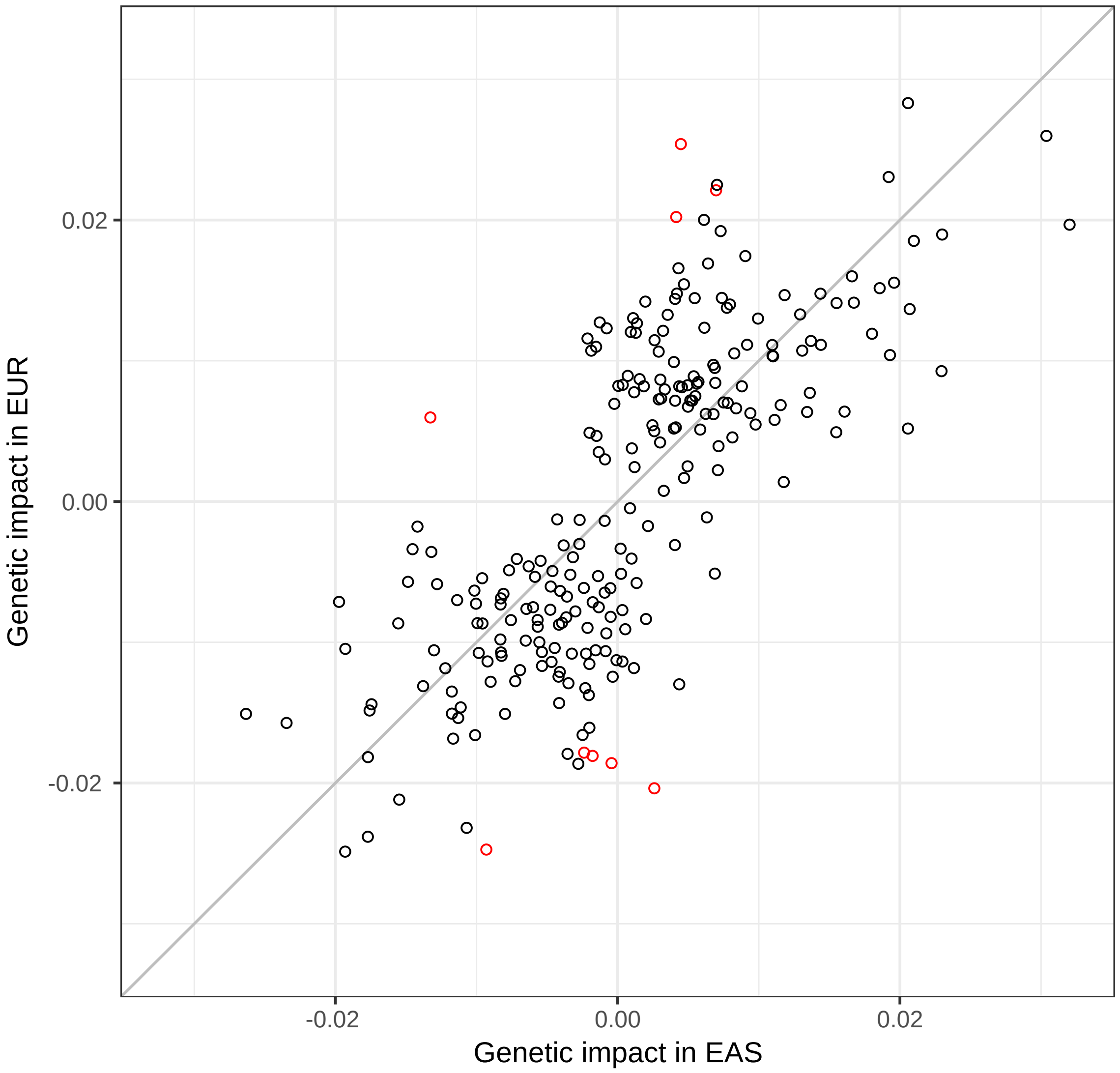

Supplementary Figure 10. Interethnic heterogeneity of genetic impact on blood pressure at 242 non-rare blood pressure loci. Refer to **Supplementary Data 8** about the details of investigated SNP loci. Nine SNPs colored in red show significant heterogeneity ($P_{hetero} < 2.1 \times 10^{-4}$).

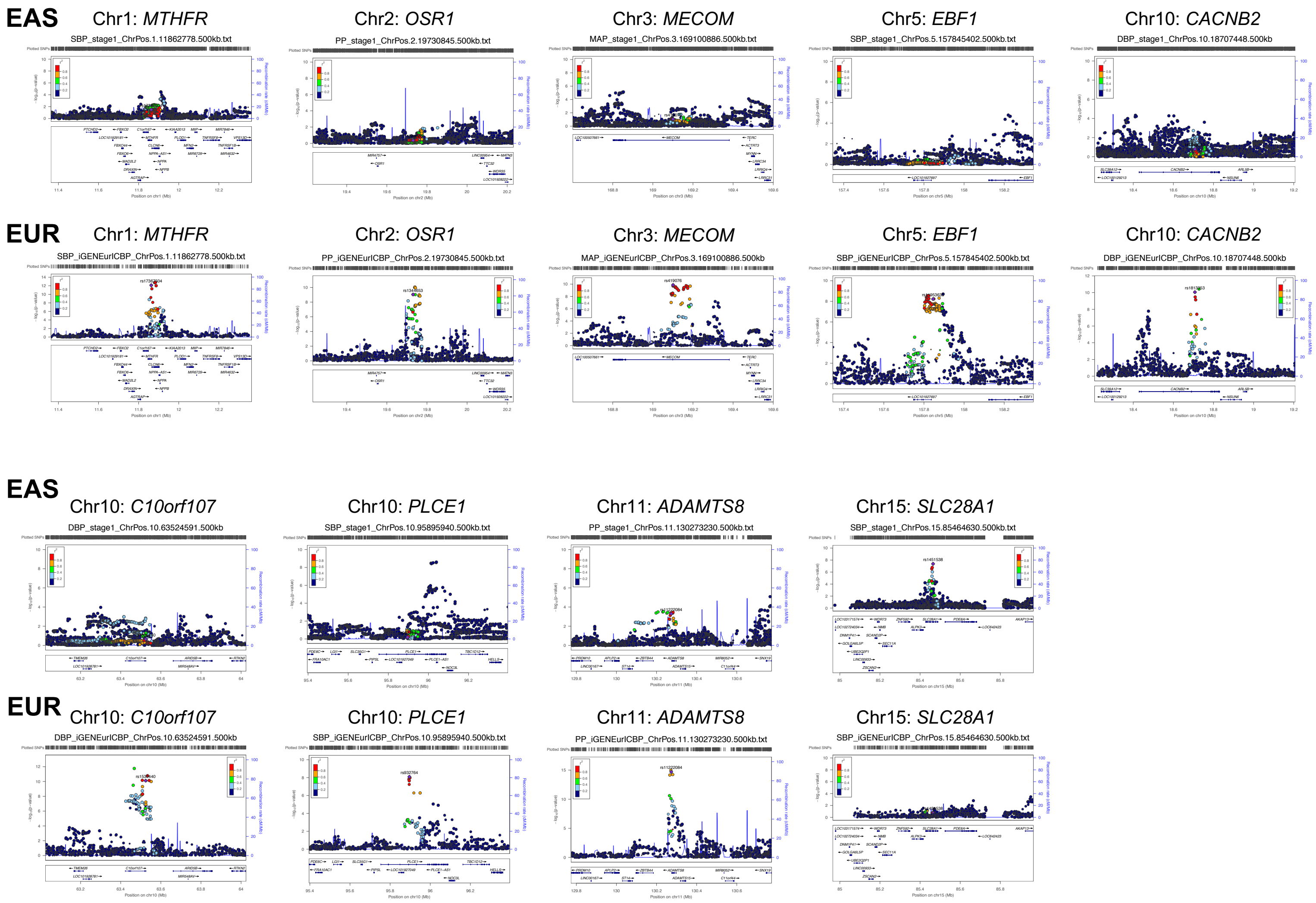

Supplementary Figure 11. Regional association plots of 9 loci showing significant interethnic heterogeneity in group 1 (MAF ≥ 0.01 in both populations). At each locus (designated by chromosome number and nearby gene name), regional association plots are demonstrated separately for East Asians (EAS, top) and Europeans (EUR, bottom). See the legends of **Supplementary Figure 4** with regard to the details about the plots.

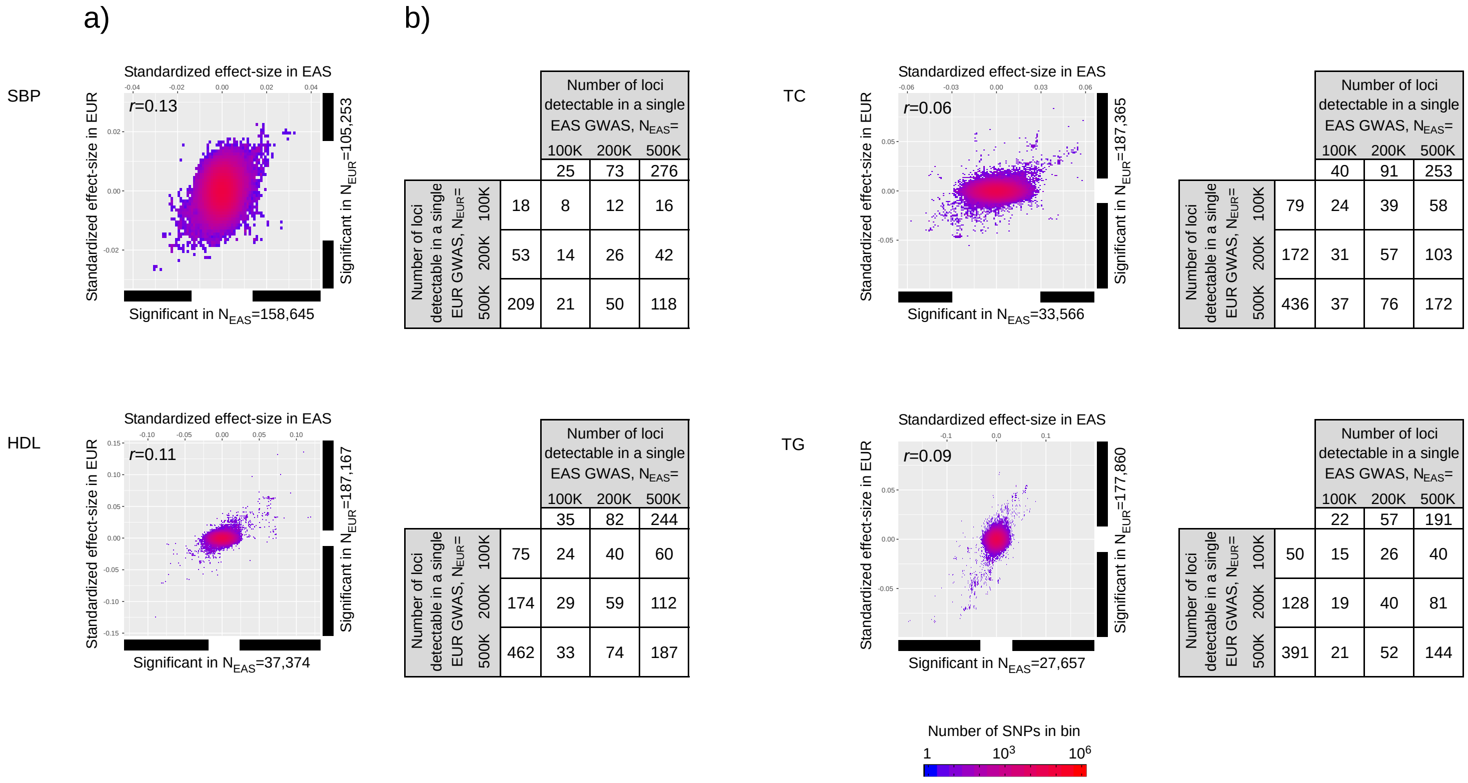

SBP

Number of loci detectable in a single EUR GWAS, N_{EUR}=		Number of loci detectable in a single EAS GWAS, N_{EAS}= 100K	200K	500K
		25	73	276
100K	18	8	12	16
200K	53	14	26	42
500K	209	21	50	118

TC

Number of loci detectable in a single EUR GWAS, N_{EUR}=		Number of loci detectable in a single EAS GWAS, N_{EAS}= 100K	200K	500K
		40	91	253
100K	79	24	39	58
200K	172	31	57	103
500K	436	37	76	172

HDL

Number of loci detectable in a single EUR GWAS, N_{EUR}=		Number of loci detectable in a single EAS GWAS, N_{EAS}= 100K	200K	500K
		35	82	244
100K	75	24	40	60
200K	174	29	59	112
500K	462	33	74	187

TG

Number of loci detectable in a single EUR GWAS, N_{EUR}=		Number of loci detectable in a single EAS GWAS, N_{EAS}= 100K	200K	500K
		22	57	191
100K	50	15	26	40
200K	128	19	40	81
500K	391	21	52	144

Supplementary Figure 12. Distribution of SNP effect-size in GWAS and power of GWAS. They are compared between East Asians and Europeans for SBP, high-density lipoprotein cholesterol (HDL-C), total cholesterol and triglycerides. (**a**) Distribution of SNP effect-size in actual GWAS conducted in East Asians (*x*-axis) and Europeans (*y*-axis). The effect-size of a SNP was standardized such that each of the trait and allele has a unit variance. A positive effect-size indicates a higher trait value for the ALT allele compared to the REF allele of the 1000 Genomes (1000G) data set. The horizontal and vertical bars to the bottom and right of the plots indicate the range of effect sizes, in which genome-wide significant SNPs are localized. (**b**) The expected numbers of genome-wide significant loci detectable in a single GWAS and their interethnic overlap. The number of SNPs was scaled to 1000G SNPs even for GWAS in which HapMap-derived SNPs were assayed. SNPs located ≤500 kb were regarded to be at the same susceptibility locus. The numbers of loci were inferred from the heritability model shown in **Supplementary Figure 11**, where 'true' observable effect sizes were computed based on 100 trials of random sampling under the assumed heritability parameters (see Methods).

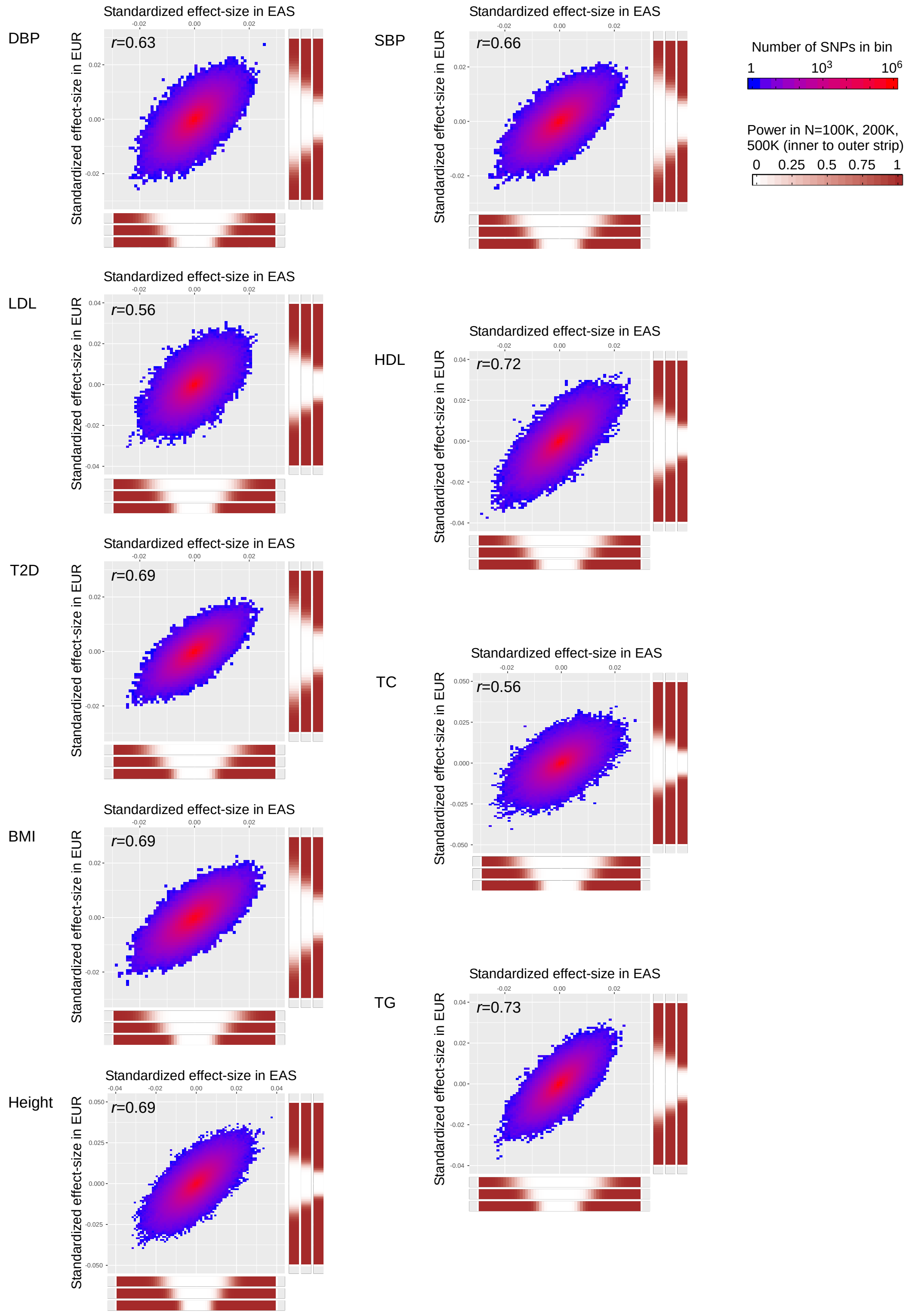

Supplementary Figure 13. SNP effect-size distributions for GWASs of complex traits modeled on the basis of the observed heritability parameters. The horizontal and vertical bars to the bottom and right of the plots indicate the range of power in different sizes, with 100K, 200K and 500K arranged from the inner to outer strips.

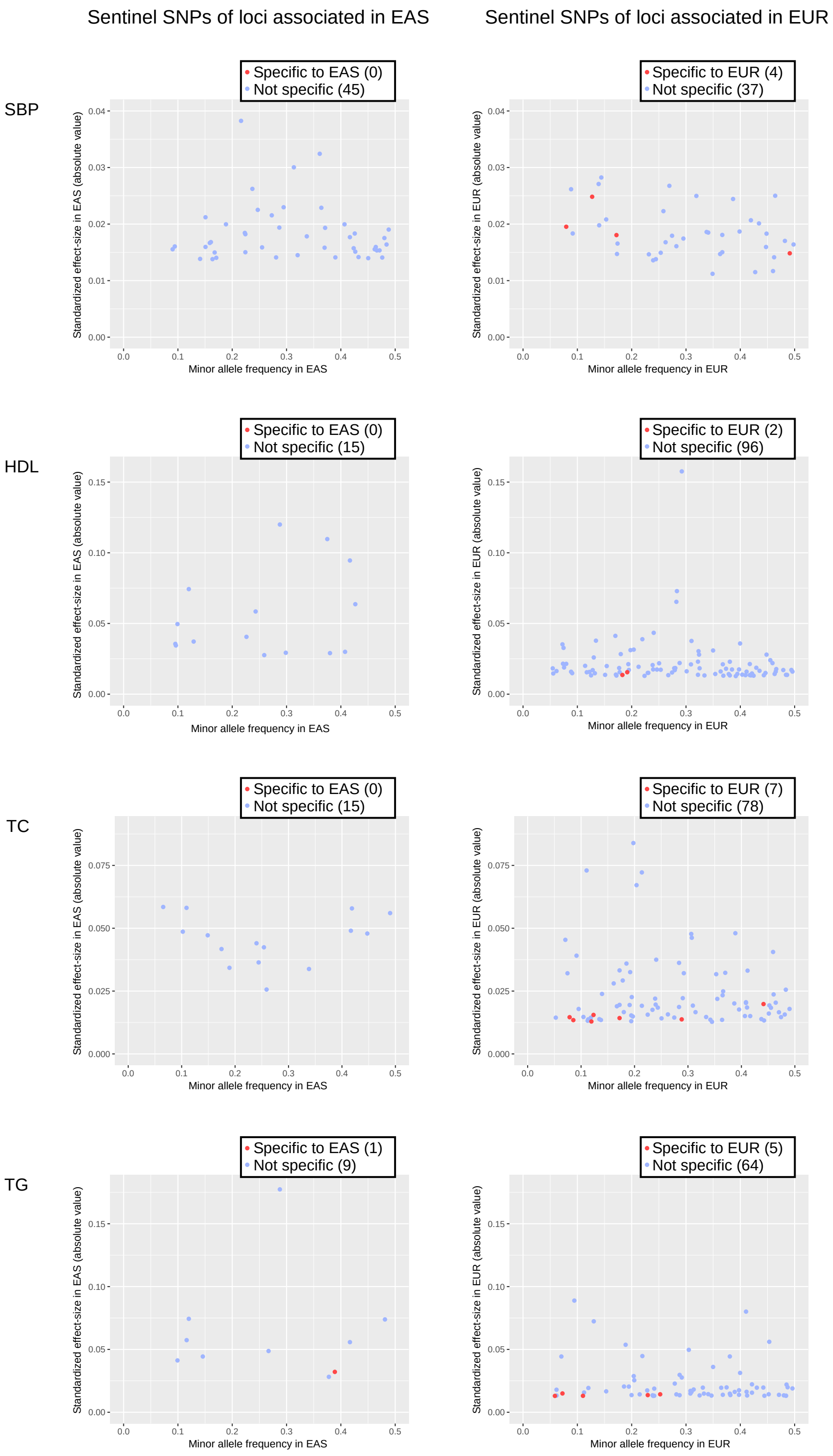

Supplementary Figure 14. Interethnic comparability of GWAS results for SBP, HDL-C, total cholesterol and triglycerides. Each point in the plots represents a sentinel SNP with genome-wide significance in the GWAS summary statistics (**Supplementary Table 6**), plotted with its standardized effect-size (in *y*-axis) against minor allele frequency (in *x*-axis) for East Asians (in the left column) and Europeans (in the right column). SNPs specific to either of the ethnic groups are colored in red; ancestry-specific association was defined such that the sentinel SNPs at the corresponding loci reached genome-wide significance ($P < 5 \times 10^{-8}$) in one ethnic group but were non-polymorphic or rare (MAF < 0.05) in another ethnic group.

Supplementary Table 1 - Characteristics of East Asian participants in the GWAS stage-1 and stage-2 cohorts

Study name	Ethnicity	Country	Design (either Population, Case-cohort or Family-based cohort)	Sample size for BP	%women	Age, Mean (SD)	BMI, Mean (SD)	SBP, Mean (SD) in mmHg	DBP, Mean (SD) in mmHg	%hyper-tension	%antihypertensive therapy	Sample size of HT cases	Sample size of HT controls
**Stage 1 cohorts**													
BBJ	Japanese	Japan	Case-cohort	125,778	46.82	62.51 (13.84)	23.33 (3.68)	129.33 (17.03)	75.87 (10.85)	40.97	38.02	21,311	26,536
CAGE-Amagasaki	Japanese	Japan	Population	559	31	46.4 (12.5)	22.8 (2.9)	124.4 (19.1)	76.3 (12.4)	31.8	6.5	57	121
CAGE-GWAS1	Japanese	Japan	Population	1547	42.8	66.1 (8.0)	23.5 (3.3)	134.1 (20.3)	76.8 (11.9)	56.1	37.9	842	678
CAGE-KING-Omni2	Japanese	Japan	Population	527	42.1	63.1 (5.3)	22.9 (2.9)	132.4 (20.4)	77.6 (11.7)		26.2	91	192
CAGE-KING-OmniE1	Japanese	Japan	Population	817	59.4	63.2 (6.3)	22.7 (3.0)	131.3 (20.7)	77.3 (11.7)		24.1	122	307
CAGE-KING-OmniE2	Japanese	Japan	Population	615	75.1	62.0 (6.3)	22.6 (3.0)	129.0 (18.0)	75.0 (10.4)		21.5	92	270
CAGE-KING-Quad	Japanese	Japan	Population	494	21.5	69.5 (3.8)	23.2 (2.7)	138.4 (19.9)	78.5 (10.4)		29.6	NA	NA
AASC	Japanese	Japan	Population	448	60	64.8 (10.6)	23.2 (3.0)	133.7 (19.3)	75.6 (10.8)		31.2	51	122
**Stage 2 cohorts**													
BES-610	Chinese	China	Population	819	64.45	58.46 (9.24)	25.7 (3.79)	136.36 (12.54)	80.97 (6.28)	10.01	10.13	82	192
BES-omni	Chinese	China	Population	806	58.84	61.7 (9.64)	25.39 (3.48)	137.3 (14.39)	80.87 (7.69)	21.71	27.92	175	206
CHNS	Chinese	China	Population	5,008	54.25	57.28 (6.50)	23.89 (4.75)	125.91 (18.71)	80.01 (11.09)	7.64	3.89	383	1103
CLHNS	Filipino	Phillipines	Population	1,786	100	48.44 (6.10)	24.31 (4.37)	119.60 (20.09)	79.62 (12.48)	11.25	3.86	201	366
TMM CommCohort Study	Japanese	Japan	Population	9892	65.14	60.43 (11.19)	23.55 (3.56)	127.74 (18.75)	75.12 (11.58)	31.14	25.51	1323	2925
GenSalt	Han Chinese	China	Family-based cohort	1881	47.21	38.73 (9.53)	23.35 (3.18)	116.92 (14.21) *	73.73 (10.30) *	1.33	0.37	25	133
TWSC	Han Chinese	Taiwan	Population	2085	50.22	50.42 (16.87)	24.47 (3.98)	128.19 (21.63)	81.38 (13.19)	21.63	17.12	247	350
HEXA	Korean	Korea	Population	3695	55.4	53.22 (8.32)	23.96 (2.90)	121.69 (14.37)	77.05 (9.90)	20.97	NA	775	1060
KARE	Korean	Korea	Population	8840	52.7	52.22 (8.92)	24.60 (3.12)	117.60 (18.28)	75.07 (11.56)	13.29	10.88	1175	2651
living-biobank-CHS	Chinese	Singapore	Population	1222	49.75	35.63 (10.6)	23.32 (4.13)	114.73 (16.41)	69.28 (10.93)	1.06	7.04	13	45
living-biobank-MAS	Malay	Singapore	Population	1135	49.78	37.05 (11.09)	26.01 (5.27)	119.13 (17.68)	70.40 (11.17)	0	6.26	0	53
NHAPC	Chinese	China	Population	2891	56.8	58.64 (6.01)	24.46 (3.63)	144.41 (25.90)	82.98 (12.85)	37.29	28.29	1078	900
SCES-610	Chinese	Singapore	Population	1882	48.88	58.47 (9.53)	23.74 (3.55)	140.66 (21.65)	81.33 (10.82)	22.79	33.48	429	314
SCES-omni	Chinese	Singapore	Population	613	48.78	60.29 (9.57)	23.78 (3.82)	141.83 (21.71)	80.56 (10.82)	23.33	37.68	143	111
SCHS-CHDCases	Singapore Chinese	Singapore	Case-cohort	673	35.4	66.64 (7.83)	23.25 (3.48)	152.45 (29.90)	85.95 (15.71)	35.21	44.13	237	90
SCHS-CHDControls	Singapore Chinese	Singapore	Case-cohort	1218	36.1	66.26 (7.82)	22.83 (3.27)	145.76 (27.98)	82.09 (14.64)	23.64	32.41	287	315
SiMES	Malay	Singapore	Population	2531	50.57	59.07 (11.04)	26.36 (5.09)	151.32 (25.48)	82.38 (11.91)	24.22	23.07	613	255
SMHS	Chinese	China	Population	872	0	62.83 (9.07)	23.82 (3.17)	132.16 (18.55)	82.88 (10.09)	38.42	32	335	537
SP2-1M	Chinese	Singapore	Population	951	36.28	46.95 (10.34)	22.89 (3.47)	130.86 (20.41)	79.49 (12.07)	11.99	12.2	114	83
SP2-550	Chinese	Singapore	Population	335	23.88	49.71 (12.76)	23.63 (3.53)	137.44 (23.54)	82.39 (12.86)	17.61	19.7	59	40
SP2-610	Chinese	Singapore	Population	1146	76.61	48.67 (11.37)	22.62 (3.88)	130.31 (23.24)	76.13 (11.57)	13	14.49	149	167
SWHS	Chinese	China	Population	2733	100	57.78 (8.81)	25.17 (3.60)	127.65 (19.35)	79.99 (10.47)	35.35	24.26	966	1767

Supplementary Table 2 - Genotyping, imputation and association testing in the GWAS stage-1 and stage-2 cohorts

Study name	Genotyping platform	Genotype calling algorithm	Pre-imputation QC: Sample call rate cut-off	Pre-imputation QC: SNP call rate cut-off	Pre-imputation QC: SNP MAF cut-off	Pre-imputation QC: SNP HWE P cut-off	#SNPs for imputation	Imputation reference	Imputation software	Association study software	Study specific covariates (eg. PC) for association study	Genomic control lambda (LD score regression intercept)				
												DBP	SBP	MAP	PP	HT
**Stage 1 cohorts**																
BBJ	Illumina HumanOmniExpressExome OR IlluminaHuman OmniExpress + IlluminaHuman Exome	GenCall	0.98	0.99	0.005	1.00E-06	508,642	1000G Phase 1 Integrated Release Version 3 Haplotypes (2010-11 data freeze, 2012-03-14 haplotypes) on 275 individuals of EAS backgrounds after excluding relatives using IBS Sites with MAF < 0.01 and pHWE<1.0e-06 or indels are excluded	MaCH/mimimac (v0.1.1)	mach2qtl/mach2dat	PC1-10, 46 Affection status at registry	1.18 (1.05)	1.24 (1.06)	1.22 (1.06)	1.15 (1.04)	1.22 (1.04)
CAGE-Amagasaki	Illumina Omni2.5	Beadstudio	0.95	0.95	0.01	1.00E-06	1,226,469	1000G phase3v5; 2504 individuals of all ancestry	SHAPEIT/minimac3	mach2qtl v113	None	1.00 (1.00)	0.99 (1.01)	0.99 (1.00)	0.97 (1.01)	1.12 (1.00)
CAGE-GWAS1	Illumina 550K/610K	Beadstudio	0.90	0.95	0.01	1.00E-06	399,413	1000G phase3v5; 2504 individuals of all ancestry	SHAPEIT/minimac3	mach2qtl v113	None	1.01 (0.99)	1.02 (1.00)	1.01 (0.99)	1.00 (1.01)	1.04 (1.00)
CAGE-KING-Omni2	Illumina Omni2.5-8	GenomeStudio	0.95	0.98	0.01	1.00E-06	1,189,396	1000G phase3v5; 2504 individuals of all ancestry	SHAPEIT2/minimac3	mach2qtl v113, mach2dat v124	first 10 PCs	1.01 (0.99)	1.01 (0.99)	1.01 (0.99)	1.01 (0.99)	0.98 (1.00)
CAGE-KING-OmniE1	Illumina OmniExpress-12	GenomeStudio	0.95	0.98	0.01	1.00E-06	565,352	1000G phase3v5; 2504 individuals of all ancestry	SHAPEIT2/minimac3	mach2qtl v113, mach2dat v124	first 10 PCs	0.99 (1.02)	0.99 (1.01)	0.99 (1.02)	0.98 (1.00)	0.97 (1.02)
CAGE-KING-OmniE2	Illumina OmniExpress-24	GenomeStudio	0.95	0.98	0.01	1.00E-06	533,819	1000G phase3v5; 2504 individuals of all ancestry	SHAPEIT2/minimac3	mach2qtl v113, mach2dat v124	first 10 PCs	1.00 (1.01)	1.00 (1.00)	1.00 (1.00)	1.00 (0.99)	0.98 (1.00)
CAGE-KING-Quad	Illumina 660W-Quad	BeadStudio	0.95	0.98	0.01	1.00E-06	461,200	1000G phase3v5; 2504 individuals of all ancestry	SHAPEIT2/minimac3	mach2qtl v113	first 10 PCs	1.00 (0.99)	1.00 (0.98)	1.00 (0.98)	1.00 (0.99)	NA
AASC	Illumina Omni2.5-4	Beadstudio	0.95	0.99	0.01	1.00E-06	1,236,886	1000G phase3v5; 2504 individuals of all ancestry	SHAPEIT2/minimac3	PLINK2 version 1.09	None	1.02 (1.01)	1.01 (1.00)	1.02 (1.01)	1.01 (1.00)	0.89 (0.94)
**Stage 2 cohorts**																
BES-610	Illumina 610Quad	Genomestudio GenTrain and GenCall	0.95	0.95	NA	5.00E-05	538,970	1000 Genomes Phase 3 version 5 Mixed	Michigan Imputation Server	EPACTS	NA					
BES-omni	Illumina Omniexpress	Genomestudio GenTrain and GenCall	0.95	0.95	NA	5.00E-05	575,352	1000 Genomes Phase 3 version 5 Mixed	Michigan Imputation Server	EPACTS	NA					
CHNS	Illumina HumanCoreExome	Birdseed	0.98	0.95	0.01	1.00E-05	534,143	1000 Genomes Phase 3	Minimac3 (MI Imputation Server)	EPACTS	PC1					
CLHNS	AffymetrixSNP 5	Birdseed	0.98	0.95	0.01	1.00E-05	352,264	1000 Genomes Phase 3	Minimac3 (MI Imputation Server)	EPACTS	PC1					
TMM CommCohort Study	HumanOmniExpressExome-8 v1.2	Genomestudio GenTrain and GenCall	0.98	0	0.95	5.00E-02	490,981	ToMMo 2KJPN panel 1000 Genomes Phase 3	SHAPEIT2/IMPUTE2	GCTA	NA (population stratification and cryptic relatedness corrected as random effects)					
GenSalt	Affymetrix 6.0	Birdseed	None	≥0.75 if MAF<0.01	0	≥1E-6	820,015	1000 Genomes Phase 3	MACH v.1.0.16	SAS 9.3 Mixed Model	Field center					
TWSC	Illumina HumanHap550-Duo	BeadStudio	0.95	0.95	NA	1.00E-04	594,076	1000 Genomes Phase 3 version 5 EAS	IMPUTE2	SNPTEST	NA					
HEXA	Affymetrix 6.0	Birdseed	0.95	0.95	0.01	1.00E-06	643,114	1000 Genomes Phase 3	IMPUTE2	SNPTEST	NA					
KARE	Affymetrix 5.0	Birdseed	0.98	0.95	0.01	1.00E-06	352,228	1000 Genomes Phase 3	IMPUTE2	SNPTEST	Recruitment area					
living-biobank-CHS	Illumina Omniexpress	Genomestudio GenTrain and GenCall	0.95	0.95	NA	1.00E-06	700,826	1000 Genomes Phase 3 version 5 Mixed	Michigan Imputation Server	EPACTS	NA					
living-biobank-MAS	Illumina Omniexpress	Genomestudio GenTrain and GenCall	0.95	0.95	NA	1.00E-06	700,826	1000 Genomes Phase 3 version 5 Mixed	Michigan Imputation Server	EPACTS	PC1, PC2					
NHAPC	Illumina 660W-Quad	GenomeStudio	0.97	0.95	0.01	1.00E-06	482,969	1000 Genomes Phase 3	IMPUTE2	SNPTEST	Region(Beijing/Shanghai)					
SCES-610	Illumina 610Quad	Genomestudio GenTrain and GenCall	0.95	0.95	NA	1.00E-06	532,116	1000 Genomes Phase 3 version 5 Mixed	Michigan Imputation Server	EPACTS	NA					
SCES-omni	Illumina Omniexpress	Genomestudio GenTrain and GenCall	0.95	0.95	NA	5.00E-05	629,062	1000 Genomes Phase 3 version 5 Mixed	Michigan Imputation Server	EPACTS	NA					
SCHS-CHDCases	Illumina OmniZhonghua	GenomeStudio	0.95	0.95	0.01	1.00E-05	846,664	1000 Genomes Phase 3 Version 5 All individuals	SHAPEIT2/IMPUTE2	SNPTEST2	PC1-3					
SCHS-CHDControls	Illumina OmniZhonghua	GenomeStudio	0.95	0.95	0.01	1.00E-05	846,664	1000 Genomes Phase 3 Version 5 All individuals	SHAPEIT2/IMPUTE2	SNPTEST2	PC1-3					
SiMES	Illumina 610Quad	Genomestudio GenTrain and GenCall	0.95	0.95	NA	1.00E-06	549,947	1000 Genomes Phase 3 version 5 Mixed	Michigan Imputation Server	EPACTS	PC1, PC2					
SMHS	Illumina Omniexpress	GenomeStudio	0.95	0.95	0.01	1.00E-05	533,842	1000 Genomes Phase 3	Minimac3 (MI Imputation Server)	maq2qtl	PC1-3					
SP2-1M	Illumina 1M duov3	Genomestudio GenTrain and GenCall	0.95	0.95	NA	1.00E-06	929,398	1000 Genomes Phase 3 version 5 Mixed	Michigan Imputation Server	EPACTS	NA					
SP2-550	Illumina 550 duo	Genomestudio GenTrain and GenCall	0.95	0.95	NA	1.00E-06	495,823	1000 Genomes Phase 3 version 5 Mixed	Michigan Imputation Server	EPACTS	NA					
SP2-610	Illumina 610Quad	Genomestudio GenTrain and GenCall	0.95	0.95	NA	1.00E-06	535,198	1000 Genomes Phase 3 version 5 Mixed	Michigan Imputation Server	EPACTS	NA					
SWHS	Affymetrix 6.0	Birdseed	0.95	0.98	0.01	1.00E-05	615,547	1000 Genomes Phase 3	Minimac3 (MI Imputation Server)	maq2qtl	PC1-3					

Supplementary Table 3 - Expression QTLs associated with the sentinel SNPs at the newly identified loci

Locus no.	eGene				eQTL association of sentinel blood pressure SNP				
	Gencode Id	Gene Symbol	Tissue	Top eVariant	Variant Id	SNP Id	P-value	Effect Size	LD (r^2) with top eVariant in EUR
2	ENSG00000169231.9	THBS3	Whole Blood	rs760077	1_155190254_T_A_b37	rs2990220	5.20E-27	0.33	0.71
2	ENSG00000231064.1	RP11-263K19.4	Whole Blood	rs4072037	1_155190254_T_A_b37	rs2990220	3.30E-09	0.28	0.88
2	ENSG00000177628.11	GBA	Artery - Tibial	rs1800442	1_155190254_T_A_b37	rs2990220	8.80E-09	-0.15	0.44
2	ENSG00000169231.9	THBS3	Adrenal Gland	rs760077	1_155190254_T_A_b37	rs2990220	2.90E-08	0.25	0.71
2	ENSG00000169231.9	THBS3	Brain - Cerebellar Hemisphere	rs760077	1_155190254_T_A_b37	rs2990220	4.20E-08	0.36	0.71
2	ENSG00000169231.9	THBS3	Brain - Cerebellum	rs760077	1_155190254_T_A_b37	rs2990220	1.20E-07	0.34	0.71
2	ENSG00000177628.11	GBA	Whole Blood	rs1800438	1_155190254_T_A_b37	rs2990220	4.70E-07	0.12	0.44
2	ENSG00000169242.7	EFNA1	Brain - Cerebellar Hemisphere	rs11264313	1_155190254_T_A_b37	rs2990220	5.40E-07	-0.31	0.36
2	ENSG00000236263.1	RP11-263K19.6	Whole Blood	rs2974935	1_155190254_T_A_b37	rs2990220	8.10E-07	0.14	0.99
2	ENSG00000177628.11	GBA	Adrenal Gland	rs2974929	1_155190254_T_A_b37	rs2990220	0.0000034	-0.16	0.99
2	ENSG00000173171.10	MTX1	Whole Blood	rs2974929	1_155190254_T_A_b37	rs2990220	0.0000072	0.085	0.99
2	ENSG00000225855.2	RUSC1-AS1	Pituitary	rs12239114	1_155190254_T_A_b37	rs2990220	0.00001	0.29	0.27
2	ENSG00000177628.11	GBA	Heart - Left Ventricle	rs3768566	1_155190254_T_A_b37	rs2990220	0.000018	-0.18	0.46
2	ENSG00000169231.9	THBS3	Pituitary	rs760077	1_155190254_T_A_b37	rs2990220	0.000021	0.3	0.71
2	ENSG00000169242.7	EFNA1	Heart - Left Ventricle	rs11264318	1_155190254_T_A_b37	rs2990220	0.000033	-0.16	0.37
3	ENSG00000188086.8	PRSS45	Pituitary	rs4075012	3_46896499_C_G_b37	rs6772151	0.000031	0.92	0.05
4	ENSG00000233885.3	YEATS2-AS1	Brain - Cerebellum	rs263034	3_183520112_G_A_b37	rs17622152	4.50E-16	0.61	0.65
4	ENSG00000233885.3	YEATS2-AS1	Brain - Spinal cord (cervical c-1)	rs2582078	3_183520112_G_A_b37	rs17622152	1.90E-14	0.74	0.65
4	ENSG00000233885.3	YEATS2-AS1	Brain - Cerebellar Hemisphere	rs5020418	3_183520112_G_A_b37	rs17622152	1.00E-11	0.63	0.64
4	ENSG00000233885.3	YEATS2-AS1	Brain - Hypothalamus	rs263020	3_183520112_G_A_b37	rs17622152	2.70E-09	0.54	0.65
4	ENSG00000233885.3	YEATS2-AS1	Brain - Cortex	rs2582078	3_183520112_G_A_b37	rs17622152	2.00E-08	0.43	0.65
4	ENSG00000114770.12	ABCC5	Whole Blood	rs6798870	3_183520112_G_A_b37	rs17622152	4.50E-08	-0.23	0.18
4	ENSG00000180834.3	MAP6D1	Brain - Cerebellum	rs2606230	3_183520112_G_A_b37	rs17622152	9.20E-08	-0.24	0.34
4	ENSG00000233885.3	YEATS2-AS1	Brain - Frontal Cortex (BA9)	rs262995	3_183520112_G_A_b37	rs17622152	0.0000015	0.4	0.62
4	ENSG00000180834.3	MAP6D1	Artery - Tibial	rs263017	3_183520112_G_A_b37	rs17622152	0.0000021	-0.25	0.50
4	ENSG00000233885.3	YEATS2-AS1	Heart - Left Ventricle	rs897604	3_183520112_G_A_b37	rs17622152	0.0000028	0.24	0.50
4	ENSG00000114770.12	ABCC5	Brain - Cerebellum	rs3792585	3_183520112_G_A_b37	rs17622152	0.0000043	0.28	0.17
4	ENSG00000233885.3	YEATS2-AS1	Artery - Tibial	rs263039	3_183520112_G_A_b37	rs17622152	0.0000085	0.19	0.64
4	ENSG00000233885.3	YEATS2-AS1	Pituitary	rs263036	3_183520112_G_A_b37	rs17622152	0.000015	0.34	0.64
4	ENSG00000180834.3	MAP6D1	Brain - Cerebellar Hemisphere	rs3792587	3_183520112_G_A_b37	rs17622152	0.000017	-0.27	0.09
6	ENSG00000229043.2	AC091729.9	Whole Blood	rs2949204	7_1141470_G_A_b37	rs78399431	1.10E-13	-0.51	0.16
6	ENSG00000229043.2	AC091729.9	Artery - Tibial	rs6463499	7_1141470_G_A_b37	rs78399431	7.40E-13	-0.48	0.16
6	ENSG00000229043.2	AC091729.9 (LOC1	Adrenal Gland	rs6962809	7_1141470_G_A_b37	rs78399431	5.60E-12	-0.68	0.16
6	ENSG00000178381.7	ZFAND2A	Heart - Atrial Appendage	rs6974054	7_1141470_G_A_b37	rs78399431	1.90E-10	-0.42	0.16
6	ENSG00000164849.7	GPR146	Whole Blood	rs76804143	7_1141470_G_A_b37	rs78399431	2.60E-10	0.23	0.50
6	ENSG00000224079.1	AC091729.7	Brain - Cerebellum	rs11766526	7_1141470_G_A_b37	rs78399431	2.80E-10	-0.73	0.51
6	ENSG00000229043.2	AC091729.9	Artery - Aorta	rs2960830	7_1141470_G_A_b37	rs78399431	3.70E-10	-0.55	0.16
6	ENSG00000178381.7	ZFAND2A	Heart - Left Ventricle	rs6956265	7_1141470_G_A_b37	rs78399431	5.30E-10	-0.36	0.15
6	ENSG00000178381.7	ZFAND2A	Whole Blood	rs2960848	7_1141470_G_A_b37	rs78399431	5.70E-10	-0.25	0.15
6	ENSG00000229043.2	AC091729.9	Heart - Atrial Appendage	rs2960830	7_1141470_G_A_b37	rs78399431	4.60E-09	-0.48	0.16
6	ENSG00000178381.7	ZFAND2A	Artery - Aorta	rs2949204	7_1141470_G_A_b37	rs78399431	3.70E-08	-0.33	0.16
6	ENSG00000229043.2	AC091729.9	Heart - Left Ventricle	rs2960830	7_1141470_G_A_b37	rs78399431	8.70E-08	-0.42	0.16
6	ENSG00000178381.7	ZFAND2A	Artery - Tibial	rs2140578	7_1141470_G_A_b37	rs78399431	1.50E-07	-0.2	0.16
6	ENSG00000229043.2	AC091729.9	Brain - Cerebellum	rs6969322	7_1141470_G_A_b37	rs78399431	4.60E-07	-0.67	0.16
6	ENSG00000146540.10	C7orf50	Brain - Frontal Cortex (BA9)	rs55729719	7_1141470_G_A_b37	rs78399431	0.000001	0.4	0.20
6	ENSG00000229043.2	AC091729.9	Brain - Cerebellar Hemisphere	rs2960830	7_1141470_G_A_b37	rs78399431	0.0000019	-0.65	0.16
6	ENSG00000229043.2	AC091729.9	Pituitary	rs2960831	7_1141470_G_A_b37	rs78399431	0.0000029	-0.56	0.16
6	ENSG00000229043.2	AC091729.9	Artery - Coronary	rs2949196	7_1141470_G_A_b37	rs78399431	0.0000033	-0.51	0.16
10	ENSG00000140105.13	WARS	Whole Blood	rs941931	14_100793431_G_A_b37	rs1535464	4.40E-25	0.54	0.67
10	ENSG00000258666.1	RP11-638I2.8	Whole Blood	rs2146106	14_100793431_G_A_b37	rs1535464	9.00E-12	0.34	0.84
10	ENSG00000197119.8	SLC25A29	Whole Blood	rs11629457	14_100793431_G_A_b37	rs1535464	9.60E-10	0.4	0.61
10	ENSG00000258666.1	RP11-638I2.8	Brain - Cerebellum	rs3736952	14_100793431_G_A_b37	rs1535464	3.20E-10	0.88	0.85
10	ENSG00000258666.1	RP11-638I2.8	Brain - Cerebellar Hemisphere	rs34040257	14_100793431_G_A_b37	rs1535464	1.70E-08	0.81	0.85
10	ENSG00000258581.2	RP11-638I2.10	Whole Blood	rs1998902	14_100793431_G_A_b37	rs1535464	3.00E-09	0.43	0.84
13	ENSG00000130517.9	PGPEP1	Adrenal Gland	rs12984770	19_18455657_T_C_b37	rs66978877	0.000013	-0.36	0.85
15	ENSG00000092758.11	COL9A3	Whole Blood	rs2294994	20_61462502_G_C_b37	rs66658258	4.3E-11	0.65	0.02
19	ENSG00000129038.11	LOXL1	Pituitary	rs4886778	15_74222987_C_T_b37	rs1078967	1.10E-10	0.48	0.21
19	ENSG00000129038.11	LOXL1	Artery - Tibial	rs74026308	15_74222987_C_T_b37	rs1078967	3.10E-09	0.16	0.95
19	ENSG00000129038.11	LOXL1	Artery - Aorta	rs12906558	15_74222987_C_T_b37	rs1078967	7.80E-07	0.2	0.07
19	ENSG00000261801.1	LOXL1-AS1	Pituitary	rs2165241	15_74222987_C_T_b37	rs1078967	0.000014	0.44	0.19

Locus number is in the order shown in Supplementary Data 2.

Associations between the expression levels of any expressed genes (eGene) and top genetic variants (eVariants) located within 1 Mb of the target gene's transcription site are demonstrated for the sentinel blood pressure SNPs that show a cis-eQTL association at a nominal P-value below the gene-level threshold in at least one tissue in the Genotype-Tissue Expression (GTEx) database.

Supplementary Table 4 - Potential coding variants at the newly identified loci

Locus no.	Sentinel SNP	R2	SNP	Allele	Symbol	Transcript	protein	aa_pos	wild_AA	mutant_ AA	Score	Prediction	Confidence	Score	Prediction
2	rs2990220	0.93	rs760077	A|T	MTX1	ENST00000368376	ENSP00000357360	63	T	S	1	TOLERATED	LOW	0	BENIGN
2	rs2990220	0.93	rs760077	A|T	MTX1	ENST00000316721	ENSP00000317106	63	T	S	1	TOLERATED	LOW	0	BENIGN
8	rs2305013	1	rs2305013	A|T	ARHGEF12	ENST00000397843	ENSP00000380942	973	Y	F	0.45	TOLERATED	HIGH	0.463	POSSIBLY DAMAGING
8	rs2305013	1	rs2305013	A|T	ARHGEF12	ENST00000532993	ENSP00000432984	870	Y	F	0.51	TOLERATED	HIGH	0.463	POSSIBLY DAMAGING
8	rs2305013	1	rs2305013	A|T	ARHGEF12	ENST00000356641	ENSP00000349056	954	Y	F	0.49	TOLERATED	HIGH	0.25	POSSIBLY DAMAGING
19	rs1078967	0.99	rs3825942	G|A	LOXL1	ENST00000261921	ENSP00000261921	153	G	D	0.2	TOLERATED	HIGH	0.435	POSSIBLY DAMAGING

Locus number is in the order shown in Supplementary Data 2.

Supplementary Table 5 - Summary of current knowledge for candidate genes at the newly identified loci

Locus no.	Sentinel SNP	Gene	Evidence linking SNP and gene*			Gene description	Gene type	GO term accession	GO term name
			eQTL	non-synonymous	colocalization				
1	rs2076460	FGR			l	FGR proto-oncogene, Src family tyrosine kinase [Source:HGNC Symbol;Acc:HGNC:3697]	protein_coding	GO:0005737	cytoplasm
2	rs2990220	EFNA1			l	ephrin A1 [Source:HGNC Symbol;Acc:HGNC:3221]	protein_coding	GO:0010719	negative regulation of epithelial to mesenchymal transition
2	rs2990220	MTX1	e	ns	l	metaxin 1 [Source:HGNC Symbol;Acc:HGNC:7504]	protein_coding	GO:0016020	membrane
2	rs2990220	GBA	e		l	glucosylceramidase beta [Source:HGNC Symbol;Acc:HGNC:4177]	protein_coding	GO:0050728	negative regulation of inflammatory response
2	rs2990220	RP11-263K19.4	e		l		antisense_RNA		
2	rs2990220	RP11-263K19.6	e		l		antisense_RNA		
2	rs2990220	RUSC1-AS1			l	RUSC1 antisense RNA 1 [Source:HGNC Symbol;Acc:HGNC:26680]	antisense_RNA		
2	rs2990220	THBS3			l	thrombospondin 3 [Source:HGNC Symbol;Acc:HGNC:11787]	protein_coding		
2	rs2990220	GON4L			l	gon-4 like [Source:HGNC Symbol;Acc:HGNC:25973]	protein_coding	GO:0005634	nucleus
2	rs2990220	MSTO1			l	misato 1, mitochondrial distribution and morphology regulator [Source:HGNC Symbol;Acc:HGNC:29678]	protein_coding		
2	rs2990220	TRIM46			l	tripartite motif containing 46 [Source:HGNC Symbol;Acc:HGNC:19019]	protein_coding	GO:0005737	cytoplasm
2	rs2990220	AL713999.1			l		protein_coding		
2	rs2990220	ASH1L			l	ASH1 like histone lysine methyltransferase [Source:HGNC Symbol;Acc:HGNC:19088]	protein_coding	GO:0005634	nucleus
2	rs2990220	ASH1L-AS1			l	ASH1L antisense RNA 1 [Source:HGNC Symbol;Acc:HGNC:44146]	antisense_RNA		
2	rs2990220	ASH1L-IT1			l	ASH1L intronic transcript 1 [Source:HGNC Symbol;Acc:HGNC:41449]	sense_intronic		
2	rs2990220	CLK2			l	CDC like kinase 2 [Source:HGNC Symbol;Acc:HGNC:2069]	protein_coding	GO:0005634	nucleus
2	rs2990220	DAP3			l	death associated protein 3 [Source:HGNC Symbol;Acc:HGNC:2673]	protein_coding		
2	rs2990220	DPM3			l	dolichyl-phosphate mannosyltransferase subunit 3 [Source:HGNC Symbol;Acc:HGNC:3007]	protein_coding	GO:0016020	membrane
2	rs2990220	FAM189B			l	family with sequence similarity 189 member B [Source:HGNC Symbol;Acc:HGNC:1233]	protein_coding	GO:0016020	membrane
2	rs2990220	FDPS			l	farnesyl diphosphate synthase [Source:HGNC Symbol;Acc:HGNC:3631]	protein_coding		
2	rs2990220	HCN3			l	hyperpolarization activated cyclic nucleotide gated potassium channel 3 [Source:HGNC Symbol;Acc:HGNC:19183]	protein_coding	GO:0006810	transport
2	rs2990220	KRTCAP2			l	keratinocyte associated protein 2 [Source:HGNC Symbol;Acc:HGNC:28942]	protein_coding		
2	rs2990220	MIR555			l	microRNA 555 [Source:HGNC Symbol;Acc:HGNC:32811]	miRNA		
2	rs2990220	MIR92B			l	microRNA 92b [Source:HGNC Symbol;Acc:HGNC:32920]	miRNA	GO:0005615	extracellular space
2	rs2990220	MUC1			l	mucin 1, cell surface associated [Source:HGNC Symbol;Acc:HGNC:7508]	protein_coding	GO:0016020	membrane
2	rs2990220	PKLR			l	pyruvate kinase L/R [Source:HGNC Symbol;Acc:HGNC:9020]	protein_coding	GO:0046872	metal ion binding
2	rs2990220	RNU6-106P			l	RNA, U6 small nuclear 106, pseudogene [Source:HGNC Symbol;Acc:HGNC:47069]	snRNA		
2	rs2990220	RNU6-1297P			l	RNA, U6 small nuclear 1297, pseudogene [Source:HGNC Symbol;Acc:HGNC:48260]	snRNA		
2	rs2990220	RNU7-150P			l		snRNA		
2	rs2990220	RP11-201K10.3			l		protein_coding		

2	rs2990220	RP11-243J18.2	I		antisense_RNA		
2	rs2990220	RP11-29H23.4	I		antisense_RNA		
2	rs2990220	RUSC1	I	RUN and SH3 domain containing 1 [Source:HGNC Symbol;Acc:HGNC:17153]	protein_coding	GO:0005634	nucleus
2	rs2990220	SCAMP3	I	secretory carrier membrane protein 3 [Source:HGNC Symbol;Acc:HGNC:10565]	protein_coding		
2	rs2990220	SLC50A1	I	solute carrier family 50 member 1 [Source:HGNC Symbol;Acc:HGNC:30657]	protein_coding	GO:0006810	transport
2	rs2990220	SYT11	I	synaptotagmin 11 [Source:HGNC Symbol;Acc:HGNC:19239]	protein_coding	GO:0016020	membrane
2	rs2990220	UBQLN4	I	ubiquilin 4 [Source:HGNC Symbol;Acc:HGNC:1237]	protein_coding	GO:0005634	nucleus
2	rs2990220	YY1AP1	I	YY1 associated protein 1 [Source:HGNC Symbol;Acc:HGNC:30935]	protein_coding		
2	rs2990220	snoU13	I	Small nucleolar RNA U13 [Source:RFAM;Acc:RF01210]	snoRNA		
3	rs6772151	PRSS45		protease, serine 45 [Source:HGNC Symbol;Acc:HGNC:30717]	protein_coding	GO:0016787	hydrolase activity
3	rs6772151	MYL3	I	myosin light chain 3 [Source:HGNC Symbol;Acc:HGNC:7584]	protein_coding	GO:0005509	calcium ion binding
4	rs17622152	MAP6D1	I	MAP6 domain containing 1 [Source:HGNC Symbol;Acc:HGNC:25753]	protein_coding	GO:0005737	cytoplasm
4	rs17622152	YEATS2-AS1	I	YEATS2 antisense RNA 1 [Source:HGNC Symbol;Acc:HGNC:41101]	antisense_RNA		
4	rs17622152	ABCC5		ATP binding cassette subfamily C member 5 [Source:HGNC Symbol;Acc:HGNC:56]	protein_coding	GO:0006810	transport
4	rs17622152	YEATS2	I	YEATS domain containing 2 [Source:HGNC Symbol;Acc:HGNC:25489]	protein_coding	GO:0005634	nucleus
4	rs17622152	AC131160.1	I		protein_coding	GO:0016020	membrane
4	rs17622152	KLHL24	I	kelch like family member 24 [Source:HGNC Symbol;Acc:HGNC:25947]	protein_coding	GO:0005737	cytoplasm
4	rs17622152	PARL	I	presenilin associated rhomboid like [Source:HGNC Symbol;Acc:HGNC:18253]	protein_coding	GO:0016020	membrane
5	rs12209106	FOXC1	I	forkhead box C1 [Source:HGNC Symbol;Acc:HGNC:3800]	protein_coding	GO:0045944	positive regulation of transcription from RNA polymerase II promoter
5	rs12209106	GMDS	I	GDP-mannose 4,6-dehydratase [Source:HGNC Symbol;Acc:HGNC:4369]	protein_coding		
6	rs78399431	AC091729.7	I		antisense_RNA		
6	rs78399431	AC091729.9	I		antisense_RNA		
6	rs78399431	C7orf50	I	chromosome 7 open reading frame 50 [Source:HGNC Symbol;Acc:HGNC:22421]	protein_coding	GO:0003723	RNA binding
6	rs78399431	GPR146	I	G protein-coupled receptor 146 [Source:HGNC Symbol;Acc:HGNC:21718]	protein_coding	GO:0016020	membrane
6	rs78399431	ZFAND2A	I	zinc finger AN1-type containing 2A [Source:HGNC Symbol;Acc:HGNC:28073]	protein_coding	GO:0008270	zinc ion binding
6	rs78399431	ADAP1	I	ArfGAP with dual PH domains 1 [Source:HGNC Symbol;Acc:HGNC:16486]	protein_coding	GO:0005634	nucleus
6	rs78399431	AC073957.15	I		antisense_RNA		
6	rs78399431	AC091729.8	I		antisense_RNA		
6	rs78399431	COX19	I	COX19, cytochrome c oxidase assembly factor [Source:HGNC Symbol;Acc:HGNC:28074]	protein_coding		
6	rs78399431	CYP2W1	I	cytochrome P450 family 2 subfamily W member 1 [Source:HGNC Symbol;Acc:HGNC:20243]	protein_coding	GO:0016020	membrane
6	rs78399431	GPER1	I	G protein-coupled estrogen receptor 1 [Source:HGNC Symbol;Acc:HGNC:4485]	protein_coding	GO:0005634	nucleus
6	rs78399431	MIR339	I	microRNA 339 [Source:HGNC Symbol;Acc:HGNC:31776]	miRNA	GO:0005615	extracellular space
6	rs78399431	RP11-449P15.1	I		antisense_RNA		
7	rs2125067	GDF10	I	growth differentiation factor 10 [Source:HGNC Symbol;Acc:HGNC:4215]	protein_coding	GO:0008083	growth factor activity
7	rs2125067	GDF2	I	growth differentiation factor 2 [Source:HGNC Symbol;Acc:HGNC:4217]	protein_coding	GO:0010596	negative regulation of endothelial cell migration

8	rs2305013	ARHGEF12		ns	l	Rho guanine nucleotide exchange factor 12 [Source:HGNC Symbol;Acc:HGNC:14193]	protein_coding	GO:0005737	cytoplasm
8	rs2305013	AP000758.1			l	Uncharacterized protein [Source:UniProtKB/TrEMBL;Acc:M0QZC6]	protein_coding		
8	rs2305013	GRIK4			l	glutamate ionotropic receptor kainate type subunit 4 [Source:HGNC Symbol;Acc:HGNC:4582]	protein_coding	GO:0006810	transport
8	rs2305013	POU2F3			l	POU class 2 homeobox 3 [Source:HGNC Symbol;Acc:HGNC:19864]	protein_coding	GO:0005634	nucleus
8	rs2305013	TMEM136			l	transmembrane protein 136 [Source:HGNC Symbol;Acc:HGNC:28280]	protein_coding	GO:0016020	membrane
9	rs5006548	FGD4			l	FYVE, RhoGEF and PH domain containing 4 [Source:HGNC Symbol;Acc:HGNC:19125]	protein_coding	GO:0046872	metal ion binding
10	rs1535464	RP11-638I2.8	e		l		antisense_RNA		
10	rs1535464	WARS			l	tryptophanyl-tRNA synthetase [Source:HGNC Symbol;Acc:HGNC:12729]	protein_coding	GO:0005634	nucleus
10	rs1535464	SLC25A29				solute carrier family 25 member 29 [Source:HGNC Symbol;Acc:HGNC:20116]	protein_coding	GO:0006810	transport
10	rs1535464	RP11-638I2.10	e		l		antisense_RNA		
10	rs1535464	SLC25A47			l	solute carrier family 25 member 47 [Source:HGNC Symbol;Acc:HGNC:20115]	protein_coding	GO:0006810	transport
10	rs1535464	WDR25			l	WD repeat domain 25 [Source:HGNC Symbol;Acc:HGNC:21064]	protein_coding		
11	rs11642015	FTO			l	FTO, alpha-ketoglutarate dependent dioxygenase [Source:HGNC Symbol;Acc:HGNC:24678]	protein_coding	GO:0035515	oxidative RNA demethylase activity
12	rs9303509	PRKCA			l	protein kinase C alpha [Source:HGNC Symbol;Acc:HGNC:9393]	protein_coding	GO:0005622	intracellular
13	rs66978877	PGPEP1	e		l	pyroglutamyl-peptidase I [Source:HGNC Symbol;Acc:HGNC:13568]	protein_coding	GO:0005737	cytoplasm
13	rs66978877	LSM4			l	LSM4 homolog, U6 small nuclear RNA and mRNA degradation associated [Source:HGNC Symbol;Acc:HGNC:17259]	protein_coding	GO:0003723	RNA binding
14	rs6021247	NFATC2			l	nuclear factor of activated T-cells 2 [Source:HGNC Symbol;Acc:HGNC:7776]	protein_coding	GO:0005634	nucleus
15	rs66658258	COL9A3			l	collagen type IX alpha 3 chain [Source:HGNC Symbol;Acc:HGNC:2219]	protein_coding		
15	rs66658258	OGFR			l	opioid growth factor receptor [Source:HGNC Symbol;Acc:HGNC:15768]	protein_coding	GO:0005634	nucleus
16	rs3853476	AC005592.2			l	SPRY4 antisense RNA 1 [Source:HGNC Symbol;Acc:HGNC:53465]	antisense_RNA		
17	rs10821808	ANK3			l	ankyrin 3 [Source:HGNC Symbol;Acc:HGNC:494]	protein_coding	GO:0010628	positive regulation of gene expression
17	rs10821808	RP11-131N11.4			l		lincRNA		
18	rs4418728	CYP26A1			l	cytochrome P450 family 26 subfamily A member 1 [Source:HGNC Symbol;Acc:HGNC:2603]	protein_coding	GO:0016020	membrane
18	rs4418728	CYP26C1			l	cytochrome P450 family 26 subfamily C member 1 [Source:HGNC Symbol;Acc:HGNC:20577]	protein_coding	GO:0055114	oxidation-reduction process
18	rs4418728	EXOC6			l	exocyst complex component 6 [Source:HGNC Symbol;Acc:HGNC:23196]	protein_coding	GO:0000145	exocyst
18	rs4418728	RP11-348J12.2			l		antisense_RNA		
19	rs1078967	LOXL1	e	ns	l	lysyl oxidase like 1 [Source:HGNC Symbol;Acc:HGNC:6665]	protein_coding		
19	rs1078967	LOXL1-AS1			l	LOXL1 antisense RNA 1 [Source:HGNC Symbol;Acc:HGNC:44169]	antisense_RNA		

Locus number is in the order shown in Supplementary Data 2. Candidate genes were selected for inclusion in the table, when any of three types of evidence (eQTL, non-synonymous and co-localization) is available.

*e: eQTL; ns: non-synonymous; l: co-localization (+/- 50kb of sentinel SNP).

Supplementary Table 6 - Data sources of GWAS summary statistics

Data usage*	Trait	Ancestry	No. of SNPs	Sample size	Study name	Reference	PMID	URL	Note
H, I	SBP, DBP, MAP, PP	EAS	6233864	130777	This study				Stage 1
H, I	SBP, DBP, MAP, PP	EAS	2485253	27868	iGEN-BP	Nat Genet 47:1282	26390057	https://www.ebi.ac.uk/ega/	Japanese studies (which overlap with stage 1 of this study) were omitted.
H, I	SBP, DBP, MAP, PP	EUR	2149719	35344	iGEN-BP	Nat Genet 47:1282	26390057	https://www.ebi.ac.uk/ega/	
H, I	SBP, DBP, MAP, PP	EUR	2398700	69909	ICBP	Nature 478:103	21909115	https://www.ncbi.nlm.nih.gov/gap	Stage 1
I	SBP, DBP	EUR	29	201745	ICBP	Nature 478:103; Table S5	21909115		
I	SBP, DBP	EUR	77, 108	140886	UK Biobank	Nat Genet 49:403; Tables S4, S5, S6	28135244		
H, I	LDL, HDL, TG, TC	EAS	2227836	34374	AGEN	Hum Mol Genet 26:1770	28334899	http://blog.nus.edu.sg/agen/summary-statistics/lipids/	Stage 1
I	LDL, HDL, TG, TC	EAS	2, 5, 4, 3	54236	AGEN	Hum Mol Genet 26:1770; Tables S5, S6	28334899		
H, I	LDL, HDL, TG, TC	EUR	2447441	187365	GLGC	Nat Genet 45:1274	24097068	http://csg.sph.umich.edu/abecasis/public/lipids2013/	
H, I	T2D	EAS	479088	25066	BBJ	Nat Comm 7:10531	26818947	https://humandbs.biosciencedbc.jp/hum0014-v5	Stage 1
I	T2D	EAS	212	55121	BBJ	Nat Comm 7:10531; Tables 1, SD1	26818947		
H, I	T2D	EUR	8075531	158186	DIAGRAM	Diabetes 66:2888	28566273	http://diagram-consortium.org/downloads.html	
H, I	BMI	EAS	5961600	158284	BBJ	Nat Genet 49:1458	28892062	https://humandbs.biosciencedbc.jp/hum0014-v5	
H, I	BMI	EUR	2554637	322154	GIANT	Nature 518:197	25673413	http://portals.broadinstitute.org/collaboration/giant/index.php/GIANT_consortium_data_files	
H, I	Height	EAS	2730895	36227	AGEN	Hum Mol Genet 24:1791	25429064		Stage 1
I	Height	EAS	211	57699	AGEN	Hum Mol Genet 24:1791; Tables 1, S1, S2, S3	25429064		Stage 2
H, I	Height	EUR	2550858	253280	GIANT	Nat Genet 11:1173	25282103	http://portals.broadinstitute.org/collaboration/giant/index.php/GIANT_consortium_data_files	

*Data were used for heritability estimation in Fig. 2 (H), or interethnic comparison of GWAS results in Fig. 4 (I).

Data in the table are used for the estimation of heritability in Fig. 2 and/or interethnic comparison of GWAS results in Fig. 4.

SUPPLEMENTARY METHODS

1. Details of GWAS stage-1 cohorts

AASC: The Anti-aging study cohort. The study subjects are middle-aged to elderly persons who were consecutive participants in the medical check-up program at Ehime University Hospital Anti-aging Center[6]. This medical check-up program is provided to general residents of Ehime Prefecture, and is specifically designed to evaluate aging-related disorders, including arteriosclerosis, cardiovascular diseases, physical function, and cognitive function. Clinical data used in this study were obtained from the personal medical check-up records of the subjects. All study procedures were approved by the ethics committees of Ehime University Graduate School of Medicine, and singed informed consent was obtained from all participants.

DNA was extracted from peripheral blood using a QIAamp DNA blood kit (Qiagen GmbH, Hilden, North Rhine-Westphalia, Germany). Genome-wide SNP genotyping was performed using a HumanOmni 2.5 BeadChip array.

BBJ: BioBank Japan. The BBJ Project (http://biobankjp.org) started at the Institute of Medical Science, the University of Tokyo in 2003[7]. So far, the BBJ has collected blood samples and clinical information from more than 200,000 individuals with 47 targeted diseases. These participants were recruited from 12 Japanese medical institutes: Osaka Medical Center for Cancer and Cardiovascular Diseases, the Cancer Institute Hospital of Japanese Foundation for Cancer Research, Juntendo University, Tokyo Metropolitan Geriatric Hospital, Nippon Medical School, Nihon University School of Medicine, Iwate Medical University, Tokushukai Hospitals, Shiga University of Medical Science, Fukujuji Hospital, National Hospital Organization Osaka National Hospital, and Iizuka Hospital.

Information on blood pressure used in this study was collected by interviews at the recruitment. The other clinical information about birth year, sex, drug for hypertension, and affected diseases was obtained from medical records. We analysed individuals aged ≥ 18 throughout the study.

For the quantitative trait analysis, we excluded individuals with cardiovascular diseases including myocardial infarction, angina, heart failure, ischemic stroke, and peripheral artery disease. After that, we calculated the *Z*-score for each trait by linear regression using age and sex, and excluded individuals out of ± 4 standard deviations.

In the case-control study for hypertension, we excluded individuals with diabetes whose serum creatinine level > 2mg/dL or with renal dialysis. As hypertension cases, we included individuals aged ≤ 65, and took drugs for hypertension and/or systolic blood pressure ≥ 160 mm Hg and diastolic blood pressure ≥ 100 mmHg. As controls, individuals aged ≥ 50 and their systolic blood pressure < 130 mmHg and diastolic blood pressure < 85 mm Hg were included, after excluding individuals with any cardiovascular diseases.

CAGE_GWAS1: Cardio-metabolic Genome Epidemiology Network. The Cardio-metabolic Genome Epidemiology (CAGE) Network is an ongoing collaborative effort to investigate genetic and environmental factors, and their interactions affecting cardiometabolic traits/disorders among Asian populations, including the Japanese, Vietnamese and Sri Lankan[8]. CAGE participants were recruited in a population-based or hospital-based setting, depending on the design of member studies. From this network sample, a total of 1,547 Japanese samples were initially used for GWAS of blood pressure (BP) and hypertension (**GWAS panel ver.1**). These subjects were enrolled at four separate sites in Japan including the Tokyo, Nagoya, Osaka and Shimane districts. For the case-control study, 842 hypertensive subjects and 678 normotensive controls of Japanese ancestry were selected from the GWASed samples in the CAGE Network (403 cases and 452 controls were included in 1,547 subjects above). Additional panels of 3,294 hypertensive subjects and 6,831 normotensive controls were selected from the CAGE Network (hospital-based and population-based samples from the Amagasaki Study, Kyushu University Fukuoka Cohort Study, and KING Study) to test the strength of the hypertension association. BP was measured using a standard mercury sphygmomanometer or a digital BP monitor (see description of the individual population-based studies below) in the seated position. The average of two or three readings was used for the analysis.

CAGE-Amagasaki: Amagasaki Study. The Amagasaki Study (CAGE-Amagasaki) is an ongoing population-based cohort study of 5,743 individuals (3,435 males and 2,310 females), aged >18 years and recruited for a baseline examination between September 2002 to August 2003[9]. Part of the Amagasaki Study samples (n=978) were included in the GWAS panel and not used for follow-up study. BP was measured using the COLIN BP-203RV-II machine (Omron Healthcare Co. Ltd., Japan) in the sitting position after at

least 5 minutes of rest. The mean of two or three measurements taken on different occasions was used in the analysis.

CAGE-KING: Kita-Nagoya Genomic Epidemiology Study. The Kita-Nagoya Genomic Epidemiology (KING) study (ClinicalTrials.gov identifier: NCT00262691) is an ongoing community-based prospective observational study of the genetic basis of cardiovascular disease and its risk factors[10]. The study recruited 3,975 Japanese subjects aged 50-80 years, who underwent community-based annual health check-ups between May 2005 and December 2007. Part of the KING Study samples (n=2453) were included in the GWAS analysis. BP was measured using the USM-700G machine (Elquest Co., Japan) in the seated position. The average of two readings was used for the analysis.

2. Details of GWAS stage-2 cohorts

BES: Beijing Eye Study. Beijing Eye Study (BES) is a population-based cross-sectional and longitudinal study of Chinese aged 40+ years residing in the village area of Yufa in Daxing District, south of Beijing, and in the Haidian urban district, north of Central Beijing. At the time of the survey in 2001, a total of 5,324 individuals were eligible to participate in the study, of which 4,439 individuals (83.4% response rate) were recruited. In 2006, all participants from the survey in 2001 were re-invited and 3,251 participants (73.2% response rate) were recruited, and blood was taken from 2,929 (90.1%). The study was re-performed in 2011, with 3468 participants (minimum age: 50 years), out of whom 2695 individuals had already participated in the baseline examination in 2001[11].

CHNS: China Health and Nutrition Survey. The China Health and Nutrition Survey (CHNS), an ongoing open cohort, international collaborative project designed to examine the effects of the health, nutrition, and family planning policies and programs implemented by national and local governments and to see how the social and economic transformation of Chinese society is affecting the health and nutritional status of its population[12]. For this study of 5,008 CHNS participants, we used data collected from participants who were age 18 years or older and not pregnant at the time of the 2009 survey, and we analyzed one member of each first-degree relative pair. Blood pressure was measured 3 consecutive times on the same day, with 10 minutes of seated

rest before the first measurement and 3–5 minutes intervals between each measurement. The average of the 3 measurements was used for analyses.

CLHNS: Cebu Longitudinal Health and Nutrition Survey. The Cebu Longitudinal Health and Nutrition Survey (CLHNS) is a community-based birth cohort study that originally enrolled 3,327 pregnant women from the Metropolitan Cebu, Philippines area in 1983-4 (3,080 singleton live births) and has since followed them and their offspring[13]. For this study of CLHNS mothers, systolic and diastolic blood pressures were measured in triplicate after a 10-minute seated rest using mercury sphygmomanometers in the 2005 survey. The average of the three measurements was used in analysis.

GenSalt: Genetic Epidemiology Network of Salt-Sensitivity. The Genetic Epidemiology Network of Salt-Sensitivity (GenSalt) study is a unique NHLBI-sponsored family feeding-study designed to examine the interaction between genes and dietary sodium and potassium intake on BP. A detailed description of the GenSalt study design and participants has been reported previously[14]. Briefly, 3,142 participants from 633 Han families from rural, north China were ascertained through a proband with untreated pre-hypertension or stage-1 hypertension identified from a population-based BP screening. A total of 1,906 GenSalt probands and their siblings, spouses, and offspring were eligible and a resulting 1,881 took part in the dietary intervention and GWAS genotyping. Three morning BP measurements were obtained according to a standard protocol during each of the 3-days of baseline observation. All BP readings were measure by trained and certified observers using a random-zero sphygmomanometer. BP was measure with the participant in the sitting position after 5 minutes of rest. In addition, participants were advised to avoid alcohol, cigarette smoking, coffee/tea, and exercise for at least 30 minutes prior to their BP measurements. The average of 9 systolic and diastolic BP measure from the 3-day baseline examinations is used in this analysis.

HEXA: Health Examinee shared control study. The HEXA cohort is one of the KoGES population-based cohorts which were initiated in 2001 aiming to identify risk factors of life-style related complex diseases such as type 2 diabetes, hypertension, and dyslipidemia. Approximately 3,700 of 1,200,000 subjects aged 40-69 from the HEXA cohort were randomly selected as a shared control group for the Korean cancer and

coronary artery disease (CAD) GWA studies. Genotyping was conducted with the Affymetrix Genome-Wide Human SNP array 6.0 in 2008[15].

KARE: Korea Association Resource study. The Korea Association Resource (KARE) study was initiated in 2007 to undertake a large-scale GWA analysis for type 2 Diabetes and numerous complex quantitative traits amongst the 10,038 participants (aged between 40 and 69) of the Ansung (n=5,018) and Ansan (n=5,020) population-based cohorts[16]. The two KARE study cohorts were established as part of the Korean Genome Epidemiology Study (KoGES) in 2001. Both cohorts were sampled from Kyung Gi-Do province, close to Seoul, the capital of the Republic of Korea and adopted the same investigational strategy. More than 260 traits have been extensively examined among KARE participants through epidemiological surveys, physical examinations, and laboratory tests. Three BP measurements were obtained from each study participant using a random zero sphygmomanometer. The average of these three measures is used in the current analysis.

LB: Living Biobank. Living Biobank is a collection of population-based Chinese, and Malay individuals from two multi-ethnic cohorts from Singapore Population Health Studies (SPHS): Multi Ethnic Cohort (MEC), and the Singapore Health 2012 (SH2012). MEC is a cohort study initiated in 2007 that aims to investigate the genetic and lifestyle factors that affect the risk of developing chronic diseases such as T2D, cardiovascular outcomes, and cancer in the Singapore citizens and permanent residents[17]. Participants completed an administered questionnaire that included food frequency and physical activity components, a health examination, and biochemistry panel. 6,147 participants were recruited over a three-year period, with 5,453 completing the health examination, and gave blood. SH2012 is a cross-sectional study conducted in 2012 that is modelled after 5-yearly National Health Survey. Similar questionnaire, health examination, and biochemistry were administered, with additional health care financing components. At the end of recruitment, 1,956 participants completed a questionnaire, and 1,920 gave blood. Living Biobank is a joint collaboration with pharmaceutical company Merck, aims to build a catalogue of low frequency, and rare coding variants in East Asians, with consent for genotype-phenotype follow-up physiological experiments that would inform and prioritise plausible drug targets. SBP and DBP were measured. Hypertension

cases and controls were defined as stated in analysis protocol. A total of 2,466 Living Biobank samples (1,266 Chinese and 1,200 Malay) were genotyped on Illumina Omni-express and imputed into 1000 Genome Phase 3.

NHAPC: The Nutrition and Health of Aging Population in China. The study population consisted of 2891 unrelated Chinese Hans aged 50–70 years from the Study on Nutrition and Health of Aging Population in China. These participants were recruited from Beijing and Shanghai. The study design and data collection of this cohort study have been described in detail previously[18]. Sitting BP was measured by Omron HEM-705CP Blood Pressure Monitor three times after seated for 5 min, and the average of the last two measurements was used for analyses. For individuals taking antihypertensive medication, 10 mm Hg and 5 mm Hg were added to the observed values of SBP and DBP respectively to account for the treatment effects[19]. Samples were genotyped using high-density single-nucleotide polymorphism (SNP) marker platforms, Illumina Human 660 W in NHPAC. Samples with call rate <97 % were excluded. The samples in the NHAPC study that passed all QC criteria were then used to imputation by IMPUTE software using 1000 Genomes Phase III data. We removed all SNPs with MAF < 1 %, HWE-P < 10^{-6}, or poor imputation quality, defined as the info measure ≤0.5.

SCES: Singapore Chinese Eye Study. SCES is a population-based cross-sectional study of eye diseases in Chinese adults aged 40+ years residing in the southwestern part of Singapore. The methodology of SCES study has been described in detail previously[20]. Between February 2009 and December 2012, 3,353 participants were recruited from a pool of 4,605 eligible individuals identified by age-stratified random sampling.

SCHS-CHDC: Singapore Chinese Health Study (CHD case-control subset). The SCHS is a cohort study of 63,257 Singaporean Chinese (Hokkien or Cantonese dialect group) aged 45-74 years, and residing in public housing estates[21]. Recruitment and assessment of baseline diet and other interviews took place in the participant’s home from 1993 to 1998. Blood was collected in 28,439 participants between 2000 and 2005. The cohort has been followed up for mortality and morbidity through regular record linkage with the Singapore Cancer Registry, the Hospital Discharge Summary Database and the Singapore Registry of Births and Deaths through collaboration with the Ministry of

Health. Myocardial cases had fatal coronary heart disease or non-fatal myocardial infarction (MI) identified through the Singapore Registry of Births and Deaths and the Hospital Discharge Database respectively. For all non-fatal cases, medical records were retrieved for review by a cardiologist, and confirmed to have MI using the criteria of the Multi-Ethnic Study of Atherosclerosis. Cases of fatal cardiovascular heart disease (CHD) were only included if there was prior evidence of CHD based on the questionnaire data or the Hospital Discharge Database. We selected cases and matched controls using the risk-set sampling strategy. Controls were participants who were alive and free of history of coronary heart disease at the time of the diagnosis or death of the index cases and matched (1 to 2) for sex, dialect group, year of birth, year of recruitment and date of blood collection. Non-fasting venous blood was drawn from these subjects and used in the determination of blood lipids.

SiMES: Singapore Malay Eye Study. SiMES is a population-based cross-sectional study of Malay adults aged 40 to 79 years living in Singapore that was conducted between August of 2004 and June of 2006[22]. Of 4,168 participants initially recruited, 3,280 underwent a detailed ocular examination.

SMHS: Shanghai Men's Health Study. The SMHS is an on-going population-based prospective cohort study conducted in urban Shanghai, China[23]. All male permanent residents from eight typical communities who were 40-74 years of age and had no prior history of cancer were eligible. Trained interviewers visited the homes of 83,031 eligible men identified through the Shanghai Resident Registry who lived in the study communities during the baseline study period and recruited 61,478 (response rate of 74%) men between April 2002 and June 2006. The baseline survey was completed by in-person interview using a structured questionnaire designed to collect information on demographic characteristics, lifestyle habits, including dietary intake, cigarette smoking and alcohol consumption, medical history, and use of medications, including antihypertensive drugs and hormones. The prevalence of hypertension was assessed by the question, 'Have you ever been diagnosed with hypertension by a physician?' Two blood pressure measurements were taken for 86.4% of participants (n=52,177) after the participants sat quietly for more than five minutes using an aneroid sphygmomanometer according to a standard protocol. An average of two blood

measurements was used for this analysis. A 10-ml blood sample was collected from consented participants and processed within 6 hours and stored at -75°C (75% response rate). The cohort has been followed for multiple disease outcomes, including cancer, diabetes, and cardiovascular diseases.

SP2: Singapore Prospective Study Program. Singapore Prospective Study Program (SP2) comprised 6,968 participants in total, of the ages of between 24 and 95 years from four previous cross-sectional studies: Thyroid and Heart Study 1982 – 1984[24], National Health Survey 1992[25], National University of Singapore Heart Study 1993 – 1995[26], and the National Health Survey 1998. Each of these studies sampled randomly from the Singapore population with a disproportionate sampling scheme to increase the sample sizes of the minority ethnic groups (Malays and Asian Indians). In addition, 10,747 more participants were invited to participate in a follow up study between 2003 – 2007, of which 5,157 people completed the questionnaire and clinical examination. A total of 2,434 SP2 Chinese samples were genotyped on three arrays: Illumina1Mduov3 (N=953), Illumina Human610-Quad Beadchips (N=1146), Illumina 550 duo (N=335), and imputed into 1000 Genome Phase 3. Systolic blood pressure (SBP) and diastolic blood pressure (DBP) were measured. Hypertension cases and controls were defined as stated in analysis protocol.

SWHS: Shanghai Women's Health Study. The SWHS is a population-based prospective cohort study of 74,940 women (response rate of 92.3%), aged 40-70 years at recruitment (March, 1997 - May, 2000)[27]. Eligible women living in seven urban districts of Shanghai were recruited through in-home visits by medical professionals. At baseline, information about demographics, reproductive history, medical history, dietary habits, weight history, physical activity, and occupational history was collected, and weight and height measurements were taken. Specific questions such as: 'Have you ever been diagnosed with HT by a doctor' and 'Have you ever taken antihypertensive medications' were asked at recruitment. During the first follow-up (2000-2002; response rate of 91.3%), two blood pressure measurements were taken for study participants after they sat quietly for more than five minutes using an aneroid sphygmomanometer according to a standard protocol An average of two BP measurements was used for this analysis.

TMM CommCohort Study: Tohoku Medical Megabank Project. The Tohoku Medical Megabank Project (TMM), which is being conducted by Tohoku University Tohoku Medical Megabank Organization (ToMMo) and Iwate Medical University Iwate Tohoku Medical Megabank Organization (IMM), has been launched to realize creative reconstruction and to solve medical problems in the aftermath of the disaster[28]. Participants of this study was comprised of 10,000 participants of TMM Community Cohort Study participated in Miyagi and Iwate Prefecture in 2013. Data used in this study was obtained from specific health check-up. DNA sample was obtained with written informed consent. Based on the guideline of the Ministry of Health, Labour, and Welfare in Japan, blood pressure (BP) was measured twice by trained staff members using automatic devices. In some cases, single measurement was allowed. For subjects with two BP measurements, the average value was used for genetic analyses.

TWSC: Taiwan Super Control Study. Control participants of the Taiwan Super Control Study who took part in the current analysis included 2,085 randomly selected individuals whose genetic data were extracted from the Han‐Chinese Cell and Genome Bank in Taiwan[29]. In brief, more than 3,300 healthy controls were recruited via a stratified, 3-staged probability clustering sampling scheme through the registry of all the 329 non-aboriginal townships or city districts in Taiwan and their genomic DNA was extracted from peripheral blood using the Puregene DNA isolation kit (Gentra Systems, Minneapolis, MN, USA). Standard protocols for BP measurements established by the Nutrition and Health Survey in Taiwan were followed. BP was measured three times with two consecutive pulse measurements in between using the Omega 1400 NBP (Invivo Research Laboratories Inc., Orlando, FL, USA).

3. Details of replication study cohort

CKB: China Kadoorie Biobank. The baseline survey of the CKB study was conducted during 2004-2008 in 10 geographically diverse regions (5 rural and 5 urban) in China[30]. Within each area, all permanent residents aged 35-74 years without major disability were identified from local residential records and invited to attend a survey clinic (of whom 30% responded). Overall, 512,891 men and women aged 30-79 years were enrolled and these participants had a mean age at baseline of 52 (SD 10) years, 59% were women and 44% were resident in urban regions.

At baseline, detailed data was collected using an interviewer-administered laptop questionnaire on demographic characteristics, socioeconomic status, medical history, and lifestyle (e.g. alcohol drinking, smoking, physical activity). Physical measurements included height, weight, waist and hip circumference, heart rate. Systolic (SBP) and diastolic (DBP) blood pressure were measured twice using a UA-779 digital sphygmomanometer (A&D Instruments; Abingdon, UK) after the participant was at rest for at least 5 minutes. If the difference between the two measurements was more than 10 mm Hg for SBP, a third measurement was taken. The mean of the last two readings was used in all analyses. A non-fasting blood sample was collected for immediate random plasma glucose measurement in an aliquot and long-term storage of the remainder. Re-surveys of ~5% of randomly-selected surviving participants were conducted in 2008 and 2013-14. Study procedures and staff training were standardised across regions. Local, national and international ethics approval was obtained and all participants provided written informed consent.

Genotyping was conducted in 94,201 individuals using a customised 804,342 variant Affymetrix Axiom® array, designed following a similar approach to that used for the UK Biobank array. Approximately 150,000 variants of interest were selected on the basis of, for instance, predicted functional effect (e.g. nonsense or missense coding variants) or associations from previous GWAS. The remaining 650,000 SNPs were selected from Affymetrix's panel of 11M validated SNPs to provide whole-genome coverage specifically for the Chinese population. Imputation into the reference dataset from 2,504 individuals of 1000 genomes Phase 3 was conducted using SHAPEIT3 and IMPUTE4 (IMPUTE2 for chrX). Variant genotypes were extracted from the imputed dataset as dosages of the effect allele.

Linear regression models were used to assess associations of SNPs with BP traits after adjusting for age, age-squared, sex, mean external daytime temperature for the month and site of recruitment (minimum 5°C), and four region-specific principal components. Analyses were performed separately in each region and the region-specific estimates were subsequently combined using inverse variance-weighted fixed-effect meta-analysis.

Logistic regression was used to assess associations of SNPs with hypertension after adjusting for sex, mean temperature and four region-specific principal components. All analyses were performed separately in each region and the region-specific estimates

were subsequently combined using inverse variance weighted fixed-effect meta-analysis.

4. SNP-based heritability analysis

Genotype-phenotype model

SNP-based heritability is estimated by the restricted maximum-likelihood methods (GCTA or LDAK models), AVENGEME method or LD-score regression method[31]. Initially, per-SNP heritability was assumed to be identically distributed for all SNPs, but recently it became apparent that per-SNP heritability depends on allele frequency[32] and LD-related[33] or other functional annotations[34]. Whereas most studies of SNP-based heritability are conducted for a population of one ancestry, Brown *et al.* extended the LD-score regression method to two populations of different ancestries, and quantified transethnic genetic correlation[35]. Here, we extend Brown's formulation by incorporating the dependence of per-SNP heritability on allele frequency and LD-related functional annotations.

We consider two GWAS performed in different populations (possibly of different ancestries). Assume that N_1 individuals are genotyped for M SNPs in study 1, and N_2 individuals are genotyped for the same SNPs in study 2. The genotype matrix X_1 of study 1 has $N_1 \times M$ dimensions, and the matrix X_2 of study 2 has $N_2 \times M$ dimensions. For each SNP in each study, the genotypes of individuals are coded by the standardized allele dose. For population 1 from which study 1 is derived, the LD matrix Σ_1 is a $M \times M$-dimensional matrix showing the correlation between SNPs; Σ_1 can be approximated by $(1/N_1)X_1{}'X_1$, where we represent the transpose of a matrix by an apostrophe. Similarly, for population 2, Σ_2 can be approximated by $(1/N_2)X_2{}'X_2$. Note that all diagonal elements of Σ_1 and Σ_2 equal one.

SNPs and non-genetic factors are modeled to contribute additively to a quantitative phenotype. The allele substitution effect of SNP *j* in populations 1 and 2 are denoted by $\beta_{1,j}$ and $\beta_{2,j}$ respectively; the effect is per the standardized allele dose. The allele substitution effect should not be confused with the effect-size observable in GWAS. The latter incorporates the allele substitution effects of the SNP itself and the neighboring variants in LD. The N_1- or N_2-dimensional phenotype vectors $\mathbf{Y}_1$, $\mathbf{Y}_2$ in studies 1 and 2 are modeled as

$$\mathbf{Y}_1 = X_1\boldsymbol{\beta}_{1,\cdot} + \boldsymbol{\varepsilon}_1,$$

$$\mathbf{Y}_2 = X_2 \boldsymbol{\beta}_{2,\cdot} + \boldsymbol{\varepsilon}_2,$$

where the phenotype variance not owing to the SNPs is modeled by random vectors taken from multivariate normal distributions,

$$\boldsymbol{\varepsilon}_1 \sim \mathcal{N}\left(\mathbf{0},\ (1 - h_1^2)\, I_{N_1}\right),$$

$$\boldsymbol{\varepsilon}_2 \sim \mathcal{N}\left(\mathbf{0},\ (1 - h_2^2)\, I_{N_2}\right). \quad (*1)$$

We denote the heritability attributable to the aforementioned M SNPs in populations 1 and 2 by parameters h_1^2 and h_2^2 respectively, which are bound by $0 \le h_1^2, h_2^2 \le 1$. Genetic covariance between the populations is denoted by the parameter h_{x}, which is bound by ${h_{\mathrm{x}}}^2 \le h_1^2 \cdot h_2^2$. Genetic correlation between the populations is defined as $\rho = h_{\mathrm{x}} / \sqrt{h_1^2 h_2^2}$.

The allele substitution effects of single SNPs depend on allele frequency[32] and LD-related functional annotations[33]. According to the infinitesimal model, we model the effects $\beta_{1,j}$ and $\beta_{2,j}$ of SNP *j* as random variables distributed under the multivariate normal distribution

$$\begin{pmatrix} \beta_{1,j}/w_{1,j} \\ \beta_{2,j}/w_{2,j} \end{pmatrix} \sim \mathcal{N}\left(\begin{pmatrix} 0 \\ 0 \end{pmatrix},\ \frac{1}{M} \begin{pmatrix} h_1^2 & h_{\mathrm{x}} \\ h_{\mathrm{x}} & h_2^2 \end{pmatrix} \right), \quad (*2)$$

where $w_{1,j}$ and $w_{2,j}$ are pre-defined positive weights. The variables for different SNPs are independently distributed. The weights are defined as

$$w_{1,j} \propto \left[\left\{ f_{1,j}\left(1 - f_{1,j}\right) \right\}^{1+\alpha} \exp\left(\sum_{c=1}^{6} \tau_c^* a_c^*(j) \right) \right]^{1/2}, \quad (*3)$$

$$w_{2,j} \propto \left[\left\{ f_{2,j}\left(1 - f_{2,j}\right) \right\}^{1+\alpha} \exp\left(\sum_{c=1}^{6} \tau_c^* a_c^*(j) \right) \right]^{1/2}, \quad (*4)$$

$$\overline{{w_{1,\cdot}}^2} = \overline{{w_{2,\cdot}}^2} = 1,$$

where the last equation indicates normalization over SNPs. We denote $M \times M$-dimensional diagonal matrices with $w_{1,j}$ or $w_{2,j}$ in the diagonal as W_1 or W_2 respectively. In (*3) and (*4), the left halves within the brackets represent the dependence of per-SNP heritability on allele frequency, where $f_{1,j}$, $f_{2,j}$ are allele frequencies of SNP *j* in populations 1 and 2 respectively, and we adopt $\alpha = -0.25$ recommended by Speed *et al.*[32]. The genetic-impact and genetic-effect models by Brown *et al.*[35] correspond to the cases $\alpha = -1, 0$ respectively, omitting the right halves in the brackets. The right halves within the brackets represent the effect of six LD-related annotations identified by Gazal *et al.*[33], which are predicted allele age, levels of LD, recombination rate, nucleotide diversity, background selection statistic and CpG-

content. The annotation c for SNP j is represented by $a_c^*(j)$ which is standardized over SNPs, and the overall effect-size of annotation c is τ_c^*. Note that the LD-related annotations are invariable between populations. Since the original formulation by Gazal *et al.* was shown in additive scale, we converted the annotations to the multiplicative scale by taking the exponential. The annotations were downloaded from
https://data.broadinstitute.org/alkesgroup/LDSCORE/1000G_Phase3_baselineLD_ldscores.tgz

Under the aforementioned conditions, the phenotype mean becomes zero and the phenotype variance becomes one in study 1:

$$
\begin{aligned}
&E_{\boldsymbol{\beta}_{1,\cdot},\boldsymbol{\varepsilon}_1}\left[\frac{1}{N_1}\mathbf{Y}_1{}'\mathbf{Y}_1\right] \\
&= E_{\boldsymbol{\beta}_{1,\cdot},\boldsymbol{\varepsilon}_1}\left[\frac{1}{N_1}\left(X_1\boldsymbol{\beta}_{1,\cdot}+\boldsymbol{\varepsilon}_1\right)'\left(X_1\boldsymbol{\beta}_{1,\cdot}+\boldsymbol{\varepsilon}_1\right)\right] \\
&= E_{\boldsymbol{\beta}_{1,\cdot}}\left[\frac{1}{N_1}\boldsymbol{\beta}_{1,\cdot}{}'X_1{}'X_1\boldsymbol{\beta}_{1,\cdot}\right] + E_{\boldsymbol{\varepsilon}_1}\left[\frac{1}{N_1}\boldsymbol{\varepsilon}_1{}'\boldsymbol{\varepsilon}_1\right] \\
&= E_{\boldsymbol{\beta}_{1,\cdot}}\left[\boldsymbol{\beta}_{1,\cdot}{}'\Sigma_1\boldsymbol{\beta}_{1,\cdot}\right] + (1-h_1^2) \\
&= E_{\boldsymbol{\beta}_{1,\cdot}}\left[\left(\boldsymbol{\beta}_{1,\cdot}{}'W_1{}^{-1}\right)(W_1\Sigma_1W_1)\left(W_1{}^{-1}\boldsymbol{\beta}_{1,\cdot}\right)\right] + (1-h_1^2) \\
&= \mathrm{tr}\left((W_1\Sigma_1W_1)\left(\frac{h_1^2}{M}I_M\right)\right) + (1-h_1^2) \\
&= h_1^2 + (1-h_1^2) \\
&= 1.
\end{aligned}
$$

The fifth equality uses (*2) and Appendix A1. The variance explained by the M SNPs is h_1^2, thus it is justified to call this parameter the heritability attributable to the SNPs. The derivation for study 2 is analogous.

Relation between association statistics, LD score and heritability parameters

The *Z*-statistics for genotype-phenotype association of SNP j in studies 1 and 2 are denoted by $Z_{1,j}$ and $Z_{2,j}$ respectively. We here derive mathematically the probability distribution of the *Z*-statistics, and find that the distribution is parametrized by LD score and heritability. The *Z*-statistics can be decomposed as,

$$\begin{pmatrix}\mathbf{Z}_{1,\cdot}\\ \mathbf{Z}_{2,\cdot}\end{pmatrix}=\begin{pmatrix}\frac{1}{\sqrt{N_1}}X_1{}'\mathbf{Y}_1\\ \frac{1}{\sqrt{N_2}}X_2{}'\mathbf{Y}_2\end{pmatrix}$$

$$=\begin{pmatrix}\frac{1}{\sqrt{N_1}}X_1{}'X_1\boldsymbol{\beta}_{1,\cdot}\\ \frac{1}{\sqrt{N_2}}X_2{}'X_2\boldsymbol{\beta}_{2,\cdot}\end{pmatrix}+\begin{pmatrix}\frac{1}{\sqrt{N_1}}X_1{}'\boldsymbol{\varepsilon}_1\\ \frac{1}{\sqrt{N_2}}X_2{}'\boldsymbol{\varepsilon}_2\end{pmatrix}$$

$$=\begin{pmatrix}\frac{1}{\sqrt{N_1}}X_1{}'X_1W_1 & O\\ O & \frac{1}{\sqrt{N_2}}X_2{}'X_2W_2\end{pmatrix}\begin{pmatrix}W_1{}^{-1}\boldsymbol{\beta}_{1,\cdot}\\ W_2{}^{-1}\boldsymbol{\beta}_{2,\cdot}\end{pmatrix}\qquad (*5)$$

$$+\begin{pmatrix}\frac{1}{\sqrt{N_1}}X_1{}' & O\\ O & \frac{1}{\sqrt{N_2}}X_2{}'\end{pmatrix}\begin{pmatrix}\boldsymbol{\varepsilon}_1\\ \boldsymbol{\varepsilon}_2\end{pmatrix}.\qquad (*6)$$

The right half of term (*5) is simply denoting (*2) in $2M$ dimensions, and follows the probability distribution

$$\begin{pmatrix}W_1{}^{-1}\boldsymbol{\beta}_{1,\cdot}\\ W_2{}^{-1}\boldsymbol{\beta}_{2,\cdot}\end{pmatrix}\sim\mathcal{N}\left(\mathbf{0},\ \frac{1}{M}\begin{pmatrix}h_1^2I_M & h_{\mathrm{x}}I_M\\ h_{\mathrm{x}}I_M & h_2^2I_M\end{pmatrix}\right).$$

Thus, the term (*5) also follows a normal distribution with zero mean (see Appendix A1). The variance becomes

$$\begin{pmatrix}\frac{1}{\sqrt{N_1}}X_1{}'X_1W_1 & O\\ O & \frac{1}{\sqrt{N_2}}X_2{}'X_2W_2\end{pmatrix}\frac{1}{M}\begin{pmatrix}h_1^2I_M & h_{\mathrm{x}}I_M\\ h_{\mathrm{x}}I_M & h_2^2I_M\end{pmatrix}\begin{pmatrix}\frac{1}{\sqrt{N_1}}W_1X_1{}'X_1 & O\\ O & \frac{1}{\sqrt{N_2}}W_2X_2{}'X_2\end{pmatrix}$$

$$=\frac{1}{M}\begin{pmatrix}\frac{h_1^2}{N_1}X_1{}'X_1W_1{}^2X_1{}'X_1 & \frac{h_{\mathrm{x}}}{\sqrt{N_1N_2}}X_1{}'X_1W_1W_2X_2{}'X_2\\ \frac{h_{\mathrm{x}}}{\sqrt{N_1N_2}}X_2{}'X_2W_2W_1X_1{}'X_1 & \frac{h_2^2}{N_2}X_2{}'X_2W_2{}^2X_2{}'X_2\end{pmatrix}$$

$$\approx\frac{1}{M}\begin{pmatrix}h_1^2N_1\left(\Sigma_1W_1{}^2\Sigma_1+\frac{M}{N_1}\Sigma_1\right) & h_{\mathrm{x}}\sqrt{N_1N_2}\Sigma_1W_1W_2\Sigma_2\\ h_{\mathrm{x}}\sqrt{N_1N_2}\Sigma_2W_2W_1\Sigma_1 & h_2^2N_2\left(\Sigma_2W_2{}^2\Sigma_2+\frac{M}{N_2}\Sigma_2\right)\end{pmatrix}.\qquad (*7)$$

In the last equality, we approximated $(1/N_1{}^2)X_1{}'X_1W_1{}^2X_1{}'X_1$ with $\Sigma_1W_1{}^2\Sigma_1+(M/N_1)\Sigma_1$ in the top left matrix block (see Appendix A2), and did analogously in the bottom right block.

Since the right half of term (*6) follows the distribution of (*1), the term (*6) also follows a normal distribution with zero mean. The variance becomes

$$\begin{pmatrix} \frac{1}{\sqrt{N_1}} X_1' & O \\ O & \frac{1}{\sqrt{N_2}} X_2' \end{pmatrix} \begin{pmatrix} (1-h_1^2)\, I_{N_1} & O \\ O & (1-h_2^2)\, I_{N_2} \end{pmatrix} \begin{pmatrix} \frac{1}{\sqrt{N_1}} X_1 & O \\ O & \frac{1}{\sqrt{N_2}} X_2 \end{pmatrix}$$

$$= \begin{pmatrix} (1-h_1^2) \frac{1}{N_1} X_1' X_1 & O \\ O & (1-h_2^2)\, \frac{1}{N_2} X_2' X_2 \end{pmatrix}$$

$$= \begin{pmatrix} (1-h_1^2)\Sigma_1 & O \\ O & (1-h_2^2)\, \Sigma_2 \end{pmatrix}. \quad (*8)$$

Consequently, the *Z*-statistics follow a normal distribution with zero mean and the variance by the sum of (*7) and (*8),

$$\begin{pmatrix} \mathbf{Z}_{1,\cdot} \\ \mathbf{Z}_{2,\cdot} \end{pmatrix} \sim \mathcal{N} \left(\mathbf{0},\ \begin{pmatrix} \frac{h_1^2}{M} N_1 \Sigma_1 {W_1}^2 \Sigma_1 + \Sigma_1 & \frac{h_\mathrm{x}}{M} \sqrt{N_1 N_2} \Sigma_1 W_1 W_2 \Sigma_2 \\ \frac{h_\mathrm{x}}{M} \sqrt{N_1 N_2} \Sigma_2 W_2 W_1 \Sigma_1 & \frac{h_2^2}{M} N_2 \Sigma_2 {W_2}^2 \Sigma_2 + \Sigma_2 \end{pmatrix} \right).$$

(*9)

We can see that the variance is parameterized by the heritability $h_1^2,\ h_2^2,\ h_\mathrm{x}$, by the LD matrices $\Sigma_1,\ \Sigma_2$, and by the products $\Sigma_1 {W_1}^2 \Sigma_1,\ \Sigma_2 {W_2}^2 \Sigma_2,\ \Sigma_1 W_1 W_2 \Sigma_2$, which we call the weighted LD score matrices. Indeed, the (*j*, *j*) diagonal elements are the weighted LD scores for SNP *j* in population 1, population 2 or trans-population, respectively:

$$\ell_{1,j} = \left(\Sigma_1 {W_1}^2 \Sigma_1\right)_{j,j},$$
$$\ell_{2,j} = \left(\Sigma_2 {W_2}^2 \Sigma_2\right)_{j,j},$$
$$\ell_{\mathrm{x},j} = (\Sigma_1 W_1 W_2 \Sigma_2)_{j,j} = (\Sigma_2 W_2 W_1 \Sigma_1)_{j,j}.$$

By setting W_1 and W_2 to identity matrices, we obtain the unweighted LD score, which were used in the original LD-score regression that assumed uniform per-SNP heritability[36].

Computation of LD score in reference populations

We computed the unweighted and weighted LD scores in reference populations using the 1000 Genomes Project, phase 3 data[37]. Genotype data for East Asian populations (EAS, *N* = 504) or European ancestry populations (EUR, *N* = 503) were extracted. In each dataset, we filtered SNPs by minor allele frequency ≥ 0.05 and Hardy-Weinberg equilibrium $P \geq 10^{-6}$. The number of retained SNPs (denoted by M above) were 5,148,923, 5,942,349, 4,155,885 for EAS, EUR, EAS vs EUR, respectively. The LD scores

were computed using the popcorn program[35]. All other computations were performed using the R software.

Estimation of heritability parameters

The heritability parameters h_1^2, h_2^2 and h_x can be fitted by performing maximum-likelihood estimation[5] of the distribution in (*9) or better with a generalized distribution described below. The genetic correlation is computed as ρ= $h_\mathrm{x}/\sqrt{h_1^2 h_2^2}$. As in (*9), the GWAS *Z*-statistics have been naively assumed to follow normal distribution in SNP-based heritability literature, although the main interest was on the variance and not on the shape of the distribution. Under the normality assumption, the squared *Z*-statistics should follow the chi-squared distribution. However, we found that the distribution of squared *Z*-statistics in actual GWAS has a tail heavier than the chi-squared distribution and follows power distribution, by analyzing the residual quantile distribution plot and the Hill estimator[38]. Thus, assuming normality for the distribution of *Z*-statistics is not appropriate. The *Z*-statistics are decomposed as a sum of one factor (*5) for variation in allele substitution effect of SNPs and another factor (*6) for variation not owing to SNPs. In genomic selection methods for breeding, the SNP effect distribution is modeled more realistically with *t*-distribution than with the normal distribution[39] (*t*-distribution has a tail heavier than the normal distribution). The variation not owing to SNPs is due to environmental difference between individuals (e.g., nutrition, exercise) or trait measurement error, and can be reasonably assumed to be normally distributed. To conform with actual GWAS data, we generalized the distribution of *Z*-statistics as a sum of a multivariate *t*-distribution with the variance in (*7) and a normal distribution with the variance in (*8):

$$\begin{pmatrix}\mathbf{Z}_{1,\cdot}\\ \mathbf{Z}_{2,\cdot}\end{pmatrix} \sim t\left(\mathbf{0},\ \frac{1}{M}\begin{pmatrix} h_1^2 N_1\left(\Sigma_1 {W_1}^2\Sigma_1 + \frac{M}{N_1}\Sigma_1\right) & h_\mathrm{x}\sqrt{N_1N_2}\Sigma_1 W_1 W_2 \Sigma_2 \\ h_\mathrm{x}\sqrt{N_1N_2}\Sigma_2 W_2 W_1 \Sigma_1 & h_2^2 N_2\left(\Sigma_2 {W_2}^2\Sigma_2 + \frac{M}{N_2}\Sigma_2\right)\end{pmatrix},\ \nu\right)$$

$$+\mathcal{N}\left(\mathbf{0},\ \begin{pmatrix}(1-h_1^2)\Sigma_1 & O\\ O & (1-h_2^2)\,\Sigma_2\end{pmatrix}\right). \qquad (*10)$$

The third parameter ν of *t*-distribution is the degrees of freedom. As a special case of *t*-distribution with $\nu = \infty$ is equivalent to the normal distribution, (*9) is a special case of (*10).

For efficient computation, we performed approximate maximum-likelihood estimation by setting to zero the off-diagonal elements of the variance matrices in (*10) and by fitting h_1^2 and h_2^2 separately before fitting h_x, as done by Brown *et al.*[35]. An additional parameter *c* was added to adjust for possible confounding such as population stratification[36]. Specifically, we fit h_1^2, ν_1 and c_1 by setting to zero the off-diagonal elements of the variance matrices in

$$\mathbf{Z}_{1,\cdot} \sim t\left(\mathbf{0},\quad h_1^2\left(\frac{N_1}{M}\Sigma_1 {W_1}^2\Sigma_1 + \Sigma_1\right),\quad \nu_1\right)$$
$$+\mathcal{N}(\mathbf{0},\ c_1(1-h_1^2)\Sigma_1),$$

and analogously fit h_2^2, ν_2 and c_2 for

$$\mathbf{Z}_{2,\cdot} \sim t\left(\mathbf{0},\quad h_2^2\left(\frac{N_2}{M}\Sigma_2 {W_2}^2\Sigma_2 + \Sigma_2\right),\quad \nu_2\right)$$
$$+\mathcal{N}(\mathbf{0},\ c_2(1-h_2^2)\Sigma_2).$$

Using the obtained estimates $\widehat{h_1^2}$, $\widehat{h_2^2}$, $\widehat{c_1}$ and $\widehat{c_2}$, we fit h_x and ν by setting to zero the off-diagonal elements of each block of the variance matrices in

$$\begin{pmatrix}\mathbf{Z}_{1,\cdot}\\ \mathbf{Z}_{2,\cdot}\end{pmatrix} \sim t\left(\mathbf{0},\quad \begin{pmatrix}\widehat{h_1^2}\left(\frac{N_1}{M}\Sigma_1 {W_1}^2\Sigma_1 + \Sigma_1\right) & h_\mathrm{x}\frac{\sqrt{N_1N_2}}{M}\Sigma_1 W_1 W_2 \Sigma_2\\ h_\mathrm{x}\frac{\sqrt{N_1N_2}}{M}\Sigma_2 W_2 W_1 \Sigma_1 & \widehat{h_2^2}\left(\frac{N_2}{M}\Sigma_2 {W_2}^2\Sigma_2 + \Sigma_2\right)\end{pmatrix},\quad \nu\right)$$
$$+\mathcal{N}\left(\mathbf{0},\ \begin{pmatrix}\widehat{c_1}(1-\widehat{h_1^2})\Sigma_1 & O\\ O & \widehat{c_2}\left(1-\widehat{h_2^2}\right)\Sigma_2\end{pmatrix}\right).$$

In practice, the aforementioned approximate maximum-likelihood estimation can be performed for a subset of GWAS SNPs and with weighting[35]. Since SNPs in strong LD yield similar and redundant association statistics, we weighted the log-likelihood for each SNP by the inverse of unweighted LD score. The SNPs for fitting should be limited to a subset of SNPs for which the input data is accurate. Among the SNPs in GWAS summary statistics, we limited SNPs to those with strand unambiguous alleles by excluding A/T SNPs or C/G SNPs. We also limited SNPs to those with weighted LD score in the range of 1–200; 82–91% of the SNPs were in this range.

The standard errors of the estimates for h_1^2, h_2^2, $\rho = h_\mathrm{x}/\sqrt{h_1^2 h_2^2}$ or ν were obtained by the block jackknife method, where the genome was split into 200 windows containing the same number of SNPs.

Correlation of Z-statistics over SNPs between two GWAS

The similarity between two actual GWAS can be measured by taking the correlation of *Z*-statistics over all SNPs. (This equals the correlation of standardized effect-sizes over all SNPs.) On the other hand, genetic correlation measures the similarity of genotype-phenotype model between two populations, and is defined as the correlation of allele substitution effects, after adjusting for weights (see (*2)). The former correlation is weaker than the latter correlation, and we show that the attenuation is due to LD difference and limited sample size. By *J*, we denote a square matrix whose elements are all one. The variance of *Z*-statistics over all SNPs in study 1 is

$$E_{\boldsymbol{\beta}_{1,\cdot},\boldsymbol{\varepsilon}_1}\left[\mathrm{Var}_j\left[Z_{1,j}\right]\right] = E_{\boldsymbol{\beta}_{1,\cdot},\boldsymbol{\varepsilon}_1}\left[\mathbf{Z}_{1,\cdot}{}'\left(\frac{I_M}{M}-\frac{J_M}{M^2}\right)\mathbf{Z}_{1,\cdot}\right]$$

$$= \mathrm{tr}\left(\left(\frac{I_M}{M}-\frac{J_M}{M^2}\right)\left(\frac{h_1^2}{M}N_1\Sigma_1 {W_1}^2\Sigma_1+\Sigma_1\right)\right)$$

$$= \frac{h_1^2}{M}N_1\left(\frac{\mathrm{tr}\left(\Sigma_1 {W_1}^2\Sigma_1\right)}{M}-\frac{\mathrm{tr}\left(J_M\Sigma_1 {W_1}^2\Sigma_1\right)}{M^2}\right)+\left(\frac{\mathrm{tr}(\Sigma_1)}{M}-\frac{\mathrm{tr}(J_M\Sigma_1)}{M^2}\right)$$

$$\approx \frac{h_1^2}{M}N_1\frac{\mathrm{tr}\left(\Sigma_1 {W_1}^2\Sigma_1\right)}{M}+\frac{\mathrm{tr}(\Sigma_1)}{M}$$

$$= \frac{h_1^2}{M}N_1\overline{\ell_{1,\cdot}}+1,$$

where the second equality uses (*10) and Appendix A1. Similarly, for study 2,

$$E_{\boldsymbol{\beta}_{2,\cdot},\boldsymbol{\varepsilon}_2}\left[\mathrm{Var}_j\left[Z_{2,j}\right]\right] \approx \frac{h_2^2}{M}N_2\overline{\ell_{2,\cdot}}+1.$$

The covariance of *Z*-statistics over all SNPs between studies 1 and 2 is

$$E_{\boldsymbol{\beta}_{1,\cdot},\boldsymbol{\beta}_{2,\cdot},\boldsymbol{\varepsilon}_1,\boldsymbol{\varepsilon}_2}\left[\mathrm{Cov}_j\left[Z_{1,j},Z_{2,j}\right]\right] = E_{\boldsymbol{\beta}_{1,\cdot},\boldsymbol{\beta}_{2,\cdot},\boldsymbol{\varepsilon}_1,\boldsymbol{\varepsilon}_2}\left[\mathbf{Z}_{1,\cdot}{}'\left(\frac{I_M}{M}-\frac{J_M}{M^2}\right)\mathbf{Z}_{2,\cdot}\right]$$

$$= E_{\boldsymbol{\beta}_{1,\cdot},\boldsymbol{\beta}_{2,\cdot},\boldsymbol{\varepsilon}_1,\boldsymbol{\varepsilon}_2}\left[\begin{pmatrix}\mathbf{Z}_{1,\cdot}\\ \mathbf{Z}_{2,\cdot}\end{pmatrix}'\begin{pmatrix}O & \left(\frac{I_M}{M}-\frac{J_M}{M^2}\right)\\ O & O\end{pmatrix}\begin{pmatrix}\mathbf{Z}_{1,\cdot}\\ \mathbf{Z}_{2,\cdot}\end{pmatrix}\right]$$

$$=$$

$$\mathrm{tr}\left(\begin{pmatrix}O & \left(\frac{I_M}{M}-\frac{J_M}{M^2}\right)\\ O & O\end{pmatrix}\begin{pmatrix}\frac{h_1^2}{M}N_1\Sigma_1{W_1}^2\Sigma_1+\Sigma_1 & \frac{h_{\mathrm{x}}}{M}\sqrt{N_1N_2}\Sigma_1W_1W_2\Sigma_2\\ \frac{h_{\mathrm{x}}}{M}\sqrt{N_1N_2}\Sigma_2W_2W_1\Sigma_1 & \frac{h_2^2}{M}N_2\Sigma_2{W_2}^2\Sigma_2+\Sigma_2\end{pmatrix}\right)$$

$$= \frac{h_{\mathrm{x}}}{M}\sqrt{N_1N_2}\left(\frac{\mathrm{tr}(\Sigma_2W_2W_1\Sigma_1)}{M}-\frac{\mathrm{tr}(J_M\Sigma_2W_2W_1\Sigma_1)}{M^2}\right)$$

$$\approx \frac{h_{\mathrm{x}}}{M}\sqrt{N_1 N_2}\frac{\mathrm{tr}(\Sigma_2 W_2 W_1 \Sigma_1)}{M}$$

$$= \frac{h_{\mathrm{x}}}{M}\sqrt{N_1 N_2}\,\overline{\ell_{\mathrm{x},\cdot}},$$

where the third equality uses (*10). Consequently, the correlation of Z-statistics over all SNPs between studies 1 and 2 becomes

$$E_{\boldsymbol{\beta}_{1,\cdot},\boldsymbol{\beta}_{2,\cdot},\boldsymbol{\varepsilon}_1,\boldsymbol{\varepsilon}_2}\left[\mathrm{Cor}_j[Z_{1,j}, Z_{2,j}]\right] = E_{\boldsymbol{\beta}_{1,\cdot},\boldsymbol{\beta}_{2,\cdot},\boldsymbol{\varepsilon}_1,\boldsymbol{\varepsilon}_2}\left[\frac{\mathrm{Cov}_j[Z_{1,j},Z_{2,j}]}{\sqrt{\mathrm{Var}_j[Z_{1,j}]\,\mathrm{Var}_j[Z_{2,j}]}}\right]$$

$$\approx \frac{E_{\boldsymbol{\beta}_{1,\cdot},\boldsymbol{\beta}_{2,\cdot},\boldsymbol{\varepsilon}_1,\boldsymbol{\varepsilon}_2}\left[\mathrm{Cov}_j[Z_{1,j},Z_{2,j}]\right]}{\sqrt{E_{\beta_{1,\cdot},\varepsilon_1}\left[\mathrm{Var}_j[Z_{1,j}]\right]E_{\beta_{2,\cdot},\varepsilon_2}\left[\mathrm{Var}_j[Z_{2,j}]\right]}}$$

$$\approx \frac{\frac{h_{\mathrm{x}}}{M}\sqrt{N_1 N_2}\,\overline{\ell_{\mathrm{x},\cdot}}}{\sqrt{\left(\frac{h_1^2}{M}N_1\overline{\ell_{1,\cdot}}+1\right)\left(\frac{h_2^2}{M}N_2\overline{\ell_{2,\cdot}}+1\right)}}$$

$$= \rho\frac{\overline{\ell_{\mathrm{x},\cdot}}}{\sqrt{\overline{\ell_{1,\cdot}}\;\overline{\ell_{2,\cdot}}}}\frac{1}{\sqrt{1+\frac{M}{h_1^2 N_1\overline{\ell_{1,\cdot}}}}}\frac{1}{\sqrt{1+\frac{M}{h_2^2 N_2\overline{\ell_{2,\cdot}}}}}.$$

The last line shows that the correlation is attenuated compared to the genetic correlation ρ by the LD difference between populations (second factor; the value is 0.71 for EAS vs EUR), limited sample size of study 1 (third factor) and the limited sample size of study 2 (fourth factor).

Power calculation of GWAS under given heritability parameters

Given heritability parameters h_1^2, h_2^2, h_{x} (or equivalently ρ) and ν, we can estimate the power of a GWAS of some sample size (i.e., we can predict how many SNPs/loci could attain genome-wide significance). For the given heritability parameters, we compute the distribution of the standardized effect-size of SNPs, from which we estimate the GWAS power. The standardized effect-size of a SNP is the correlation between the SNP genotype and the phenotype. It is observed in actual GWAS as the Z-statistics divided by the square root of sample size. The probability distribution is derived from (*10) as

$$\begin{pmatrix}\frac{1}{\sqrt{N_1}}\mathbf{Z}_{1,\cdot}\\ \frac{1}{\sqrt{N_2}}\mathbf{Z}_{2,\cdot}\end{pmatrix} \sim t\left(\mathbf{0},\ \frac{1}{M}\begin{pmatrix} h_1^2\left(\Sigma_1 {W_1}^2\Sigma_1 + \frac{M}{N_1}\Sigma_1\right) & h_{\mathrm{x}}\Sigma_1 W_1 W_2 \Sigma_2 \\ h_{\mathrm{x}}\Sigma_2 W_2 W_1 \Sigma_1 & h_2^2\left(\Sigma_2 {W_2}^2\Sigma_2 + \frac{M}{N_2}\Sigma_2\right)\end{pmatrix},\ \nu\right)$$

$$+\mathcal{N}\left(\mathbf{0},\ \begin{pmatrix}(1-h_1^2)\frac{1}{N_1}\Sigma_1 & O\\ O & (1-h_2^2)\frac{1}{N_2}\ \Sigma_2\end{pmatrix}\right)$$

$$\xrightarrow[N_1,N_2\to\infty]{} t\left(\mathbf{0},\ \frac{1}{M}\begin{pmatrix}h_1^2\Sigma_1 {W_1}^2\Sigma_1 & h_x\Sigma_1 W_1 W_2\Sigma_2\\ h_x\Sigma_2 W_2 W_1\Sigma_1 & h_2^2\Sigma_2 {W_2}^2\Sigma_2\end{pmatrix},\ \ \nu\right). \qquad (*11)$$

In the last line, by taking infinitely large GWAS sample size, we obtain the distribution of true effect-size in the populations.

Since we cannot obtain closed mathematical form for the probability distribution of SNP effect-size in (*11), we perform numerical sampling. We first randomly sample *M*-dimensional vectors $\mathbf{S}_{1,\cdot}^{(1)}$, $\mathbf{S}_{2,\cdot}^{(1)}$ for populations 1 and 2 respectively, from the standardized normal distribution. To obtain samples from the distribution of (*11), we apply a series of quantile normalizations. By computing

$$\begin{pmatrix}\mathbf{S}_{1,\cdot}^{(2)}\\ \mathbf{S}_{2,\cdot}^{(2)}\end{pmatrix}=\begin{pmatrix}\Sigma_1 W_1 & O\\ O & \Sigma_2 W_2\end{pmatrix}\begin{pmatrix}\mathbf{S}_{1,\cdot}^{(1)}\\ \mathbf{S}_{2,\cdot}^{(1)}\end{pmatrix},$$

we obtain a sample $\left(\mathbf{S}_{1,\cdot}^{(2)},\ \mathbf{S}_{2,\cdot}^{(2)}\right)$ following the normal distribution

$$\begin{pmatrix}\mathbf{S}_{1,\cdot}^{(2)}\\ \mathbf{S}_{2,\cdot}^{(2)}\end{pmatrix}\sim\mathcal{N}\left(\mathbf{0},\ \begin{pmatrix}\Sigma_1 {W_1}^2\Sigma_1 & O\\ O & \Sigma_2 {W_2}^2\Sigma_2\end{pmatrix}\right).$$

Here, the distribution for SNP *j* is an uncorrelated normal distribution

$$\begin{pmatrix}S_{1,j}^{(2)}\\ S_{2,j}^{(2)}\end{pmatrix}\sim\mathcal{N}\left(\begin{pmatrix}0\\0\end{pmatrix},\ \begin{pmatrix}\ell_{1,j} & 0\\ 0 & \ell_{2,j}\end{pmatrix}\right).$$

By applying the scaling

$$\begin{pmatrix}S_{1,j}^{(3)}\\ S_{2,j}^{(3)}\end{pmatrix}=\begin{pmatrix}1/\sqrt{\ell_{1,j}} & 0\\ 0 & 1/\sqrt{\ell_{2,j}}\end{pmatrix}\begin{pmatrix}S_{1,j}^{(2)}\\ S_{2,j}^{(2)}\end{pmatrix},$$

we obtain a sample $\left(S_{1,j}^{(3)},\ S_{2,j}^{(3)}\right)$ following the standard normal distribution

$$\begin{pmatrix}S_{1,j}^{(3)}\\ S_{2,j}^{(3)}\end{pmatrix}\sim\mathcal{N}\left(\begin{pmatrix}0\\0\end{pmatrix},\ \begin{pmatrix}1 & 0\\ 0 & 1\end{pmatrix}\right).$$

By applying the scaling

$$r = \sqrt{{S_{1,j}^{(3)}}^2 + {S_{2,j}^{(3)}}^2},$$

$$\begin{pmatrix} S_{1,j}^{(4)} \\ S_{2,j}^{(4)} \end{pmatrix} = \frac{\sqrt{(\nu-2)(\exp(r^2/\nu)-1)}}{r} \begin{pmatrix} S_{1,j}^{(3)} \\ S_{2,j}^{(3)} \end{pmatrix},$$

we obtain a sample $\left(S_{1,j}^{(4)}, S_{2,j}^{(4)}\right)$ following the multivariate *t*-distribution with identity variance matrix

$$\begin{pmatrix} S_{1,j}^{(4)} \\ S_{2,j}^{(4)} \end{pmatrix} \sim t\left(\begin{pmatrix} 0 \\ 0 \end{pmatrix}, \begin{pmatrix} 1 & 0 \\ 0 & 1 \end{pmatrix}, \nu \right).$$

By applying the rotation

$$\theta = \cos^{-1}\left[\rho\, \ell_{\mathrm{x},j} / \sqrt{\ell_{1,j}\ell_{2,j}}\right],$$

$$\begin{pmatrix} S_{1,j}^{(5)} \\ S_{2,j}^{(5)} \end{pmatrix} = \begin{pmatrix} \cos\left(\frac{\pi}{4} - \frac{\theta}{2}\right) & \sin\left(\frac{\pi}{4} - \frac{\theta}{2}\right) \\ \cos\left(\frac{\pi}{4} + \frac{\theta}{2}\right) & \sin\left(\frac{\pi}{4} + \frac{\theta}{2}\right) \end{pmatrix} \begin{pmatrix} S_{1,j}^{(4)} \\ S_{2,j}^{(4)} \end{pmatrix},$$

we obtain a sample $\left(S_{1,j}^{(5)}, S_{2,j}^{(5)}\right)$ following a correlated *t*-distribution

$$\begin{pmatrix} S_{1,j}^{(5)} \\ S_{2,j}^{(5)} \end{pmatrix} \sim t\left(\begin{pmatrix} 0 \\ 0 \end{pmatrix}, \begin{pmatrix} 1 & \frac{\rho\, \ell_{\mathrm{x},j}}{\sqrt{\ell_{1,j}\ell_{2,j}}} \\ \frac{\rho\, \ell_{\mathrm{x},j}}{\sqrt{\ell_{1,j}\ell_{2,j}}} & 1 \end{pmatrix}, \nu \right).$$

By applying the scaling

$$\begin{pmatrix} S_{1,j}^{(6)} \\ S_{2,j}^{(6)} \end{pmatrix} = \begin{pmatrix} \sqrt{\frac{h_1^2}{M}\ell_{1,j}} & 0 \\ 0 & \sqrt{\frac{h_2^2}{M}\ell_{2,j}} \end{pmatrix} \begin{pmatrix} S_{1,j}^{(5)} \\ S_{2,j}^{(5)} \end{pmatrix},$$

we obtain a sample $\left(S_{1,j}^{(6)}, S_{2,j}^{(6)}\right)$ following

$$\begin{pmatrix} S_{1,j}^{(6)} \\ S_{2,j}^{(6)} \end{pmatrix} \sim t\left(\begin{pmatrix} 0 \\ 0 \end{pmatrix}, \frac{1}{M}\begin{pmatrix} h_1^2\ell_{1,j} & h_{\mathrm{x}}\ell_{\mathrm{x},j} \\ h_{\mathrm{x}}\ell_{\mathrm{x},j} & h_2^2\ell_{2,j} \end{pmatrix}, \nu \right).$$

The sample $\left(\mathbf{S}_{1,\cdot}^{(6)}, \mathbf{S}_{2,\cdot}^{(6)}\right)$ combined for all SNPs approximately follows the distribution of (*11),

$$\begin{pmatrix}\mathbf{S}_{1,\cdot}^{(6)}\\ \mathbf{S}_{2,\cdot}^{(6)}\end{pmatrix} \sim t\left(\mathbf{0},\ \frac{1}{M}\begin{pmatrix} h_1^2\Sigma_1 {W_1}^2\Sigma_1 & h_x\Sigma_1 W_1 W_2\Sigma_2 \\ h_x\Sigma_2 W_2 W_1\Sigma_1 & h_2^2\Sigma_2 {W_2}^2\Sigma_2\end{pmatrix},\ \ \nu\right).$$

The above random sampling generates one possible instance of true effect-size for the SNPs, under the assumed heritability parameters. For each parameter configuration, we performed 100 trials of random sampling. For this instance of true effect-size, the expected number of SNPs/loci to attain genome-wide significance in a GWAS of some sample size can be estimated by power calculation.

When testing association in a GWAS of *N* samples under genome-wide significance level $\alpha = 5 \times 10^{-8}$, the power to detect a SNP with standardized effect-size *R* equals

$$\int_c^\infty F\left(1,\quad N-2,\quad \frac{NR^2}{1-R^2}\right) dx,$$

where F (1, N–2, θ^2) is the probability density function for a *F*-distribution of 1 and *N*–2 degrees of freedom and non-centrality parameter θ^2, and *c* satisfies

$$\alpha = \int_c^\infty F(1,\quad N-2,\quad 0)\, dx.$$

Next, we compute the expected number of genome-wide significant loci. We regard that two significantly associated SNPs within 500 kb belong to the same locus. A locus is defined as a chromosomal region, where a group of significant SNPs are localized ≤500 kb to the adjacent ones. For a GWAS of some sample size, we first extract SNPs that can be detected with >0.05 power, and then enumerate loci for the extracted SNPs. The power to detect a locus is defined as the maximum of the power for the SNPs annotated to that locus. The sum of the power for all loci becomes the expected number of loci to attain genome-wide significance. For a pair of GWASs, we compute the number of overlapping genome-wide significant loci by i) enumerating loci and assigning the power for each locus, ii) depicting the loci that overlap that overlap between the GWASs, iii) computing the product of the power in the GWASs as the power for overlapping discovery, and iv) taking the sum.

Appendix A1.

For deriving formulae of SNP-based heritability analyses, we used the following properties of multivariate probability distributions. Let **Y** be a random vector with zero mean and variance matrix *V*, and let *A* be a square matrix. Under any distribution $\mathbf{Y}\sim(\mathbf{0},\ V)$, the expectation of the quadratic form equals the matrix trace as $E[\mathbf{Y}'A\mathbf{Y}] = \mathrm{tr}(AV)$. Under normal distribution $\mathbf{Y}\sim\mathcal{N}(\mathbf{0},\ V)$, the linear transformation is also normally distributed as $A\mathbf{Y}\sim\mathcal{N}(\mathbf{0},\ AVA')$. Under *t*-distribution $\mathbf{Y}\sim t(\mathbf{0},\ V,\ \nu)$, the linear transformation also follows *t*-distribution as $A\mathbf{Y}\sim t(\mathbf{0},\ AVA',\ \nu)$.

Appendix A2.

For any index *j*, *k*,

$$\left((1/{N_1}^2){X_1}'X_1{W_1}^2{X_1}'X_1\right)_{j,k}$$

$$= \left(({X_1}'X_1/N_1){W_1}^2({X_1}'X_1/N_1)\right)_{j,k}$$

$$= \textstyle\sum_l ({X_1}'X_1/N_1)_{j,l}{w_{1,l}}^2({X_1}'X_1/N_1)_{l,k}$$

$$\approx \textstyle\sum_l {w_{1,l}}^2\left((\Sigma_1)_{j,l}(\Sigma_1)_{l,k} + (\Sigma_1)_{j,k}/N_1\right)$$

$$= \left\{\textstyle\sum_l (\Sigma_1)_{j,l}{w_{1,l}}^2(\Sigma_1)_{l,k}\right\} + \left((\Sigma_1)_{j,k}/N_1\right)\textstyle\sum_l {w_{1,l}}^2$$

$$=\left(\Sigma_1{W_1}^2\Sigma_1 + (M/N_1)\Sigma_1\right)_{j,k}.$$

For the approximation of the third equality, note that for random variables x, y, z having true correlation coefficients $r_{x,y}, r_{x,z}, r_{y,z}$, the correlations $s_{x,y}, s_{y,z}$ computed from *N* samples satisfy $s_{x,y}\ s_{y,z} \approx r_{x,y}\ r_{y,z}+r_{x,z}/N$.

SUPPLEMENTARY NOTE 1

Study-specific acknowledgments

AASC. This work was supported by Grants for Scientific Research (24390084, 21390099, 20390185) from the Ministry of Education, Culture, Sports, Science, and Technology, Japan; a Science and Technology Incubation Program in Advanced Regions, Japan Science and Technology Agency; the Japan Atherosclerosis Prevention Fund; the Takeda Medical Research Foundation; and National Cardiovascular Research Grants.

BBJ. We would like to acknowledge the staff of RIKEN Center for Integrative Medical Sciences for genotyping and data management. The Biobank Japan project was supported by the Ministry of Education, Culture, Sports, Sciences and Technology of Japanese government and the Japan Agency for Medical Research and Development (AMED).

CAGE_GWAS1. The CAGE Network studies were supported by grants for the Core Research for Evolutional Science and Technology (CREST) from the Japan Science Technology Agency; KAKENHI (Grant-in-Aid for Scientific Research) from the Ministry of Education, Culture, Sports, Science and Technology of Japan; and the Grant and research budget of National Center for Global Health and Medicine (NCGM).

CAGE-Amagasaki. We thank Drs. Toshio Ogihara, Yukio Yamori, Akihiro Fujioka, Chikanori Makibayashi, Sekiharu Katsuya, Ken Sugimoto, Kei Kamide, and Ryuichi Morishita and the many physicians of the participating hospitals and medical institutions in Amagasaki Medical Association for their assistance in collecting the DNA samples and accompanying clinical information.

CKB. The chief acknowledgment is to the participants, the project staff, and the China National Centre for Disease Control and Prevention (CDC) and its regional offices for access to death and disease registries. The Chinese National Health Insurance scheme provides electronic linkage to all hospital treatment. The CKB baseline survey and the first re-survey were supported by the Kadoorie Charitable Foundation in Hong Kong. The long-term follow-up has been supported by the UK Wellcome Trust (202922/Z/16/Z, 104085/Z/14/Z, 088158/Z/09/Z) and grants from the National Natural Science Foundation of China (81390540, 81390541, 81390544) and from the National Key Research and Development Program of China (2016YFC0900500, 2016YFC0900501,

2016YFC0900504, 2016YFC1303904). DNA extraction and genotyping were supported by grants from GlaxoSmithKline and the UK Medical Research Council (MC_PC_13049, MC-PC-14135). The British Heart Foundation, UK Medical Research Council and Cancer Research provide core funding to the Clinical Trial Service Unit and Epidemiological Studies Unit at Oxford University for the project.

CHNS. The authors thank the National Institute for Nutrition and Health, Chinese Center for Disease Control and Prevention, the Chinese National Human Genome Center at Shanghai, the Carolina Population Center, the University of North Carolina at Chapel Hill, and all of the participants and study investigators involved in the China Health and Nutrition Survey. Data collection and analysis was supported by the Carolina Population Center (P2C HD050924, T32 HD007168), the NIH (R01HD30880, R01DK104371, P30DK056350, R24HD050924, R01HD38700, R01DK072193 and U01DK105561), the NIH Fogarty International Center (D43 TW009077, D43 TW007709), the China Ministry of Health, the Chinese National Human Genome Center at Shanghai, the China-Japan Friendship Hospital, and the Beijing Municipal Center for Disease Prevention and Control.

CLHNS. The Cebu Longitudinal Health and Nutrition Survey (CLHNS) was supported by National Institutes of Health grants DK078150, TW005596, HL085144 and TW008288 and pilot funds from RR020649, ES010126, and DK056350. We thank the Office of Population Studies Foundation research and data collection teams and the study participants who generously provided their time for this study.

GenSalt. GenSalt is supported by research grants (U01HL072507, R01HL087263, and R01HL090682) from the NHLBI, National Institutes of Health, Bethesda, Maryland. Research reported in this publication was also supported by the National Institute of General Medical Sciences of the National Institutes of Health under Award Number P20GM109036.

HEXA/KARE. This work was supported by grants from Korea Centers for Disease Control and Prevention (4845-301, 4851-302, 4851-307) and an intramural grant from the Korea National Institute of Health (2016-NI73001-00), the Republic of Korea.

KING Study. This study was supported in part by Grants-in-Aid from the Ministry of Education, Culture, Sports, Science, and Technology of Japan, including those for Funding Program for Next Generation World-Leading Researchers (NEXT Program) to S.I. (LS056), for Scientific Research (B) to M.Y. (24390169 and 16H05250) and to T.M. (25293144), and JSPS KAKENHI Grant (16H06277) to M.N.

Living Biobank. The Living Biobank (sampled from MEC and SH2012) was funded through grants from the Biomedical Research Council of Singapore (BMRC) and the National Medical Research Council of Singapore (NMRC). Genotyping was supported by Merck. Genome Institute of Singapore provided services for genotyping.

NHAPC. The NHAPC study is supported by the Major Project of the Ministry of Science and Technology of China (2016YFC1304903, 2017YFC0909700), the National Natural Science Foundation of China (81471013, 30930081, 81170734, 81321062, 81700700), and the Chinese Academy of Sciences (ZDBS-SSW-DQC-02).

SCES. The Singapore Chinese Eye Study (SCES) was supported by the National Medical Research Council, Singapore (grants 0796/2003, 1176/2008, 1149/2008, STaR/0003/2008, 1249/2010, CG/SERI/2010, CIRG/1371/2013, and CIRG/1417/2015), and Biomedical Research Council, Singapore (08/1/35/19/550 and 09/1/35/19/616). C.Y.C is supported by an award from NMRC (CSA-SI/0012/2017). We thank Yuan Shi for her contribution to statistical analysis.

SCHS-CHDC. The funding for genotyping of this cohort was supported by the HUJ-CREATE Programme of the National Research Foundation, Singapore (Project Number 370062002). The Singapore Chinese Health Study was supported by the U.S. National Institutes of Health Grant Numbers, R01CA144034 and UM1 CA182876, and by the Singapore National Medical Research Council Grant Number 1270/2010. We thank Siew-Hong Low of the National University of Singapore for supervising the field work of the Singapore Chinese Health Study and the Ministry of Health in Singapore for assistance with the identification of AMI cases via database linkages. We also acknowledge the founding, longstanding principal investigator of the Singapore Chinese Health Study, Mimi C. Yu.

SiMES. The Singapore Chinese Eye Study (SCES) and Singapore Malay Eye Study (SiMES) were supported by the National Medical Research Council, Singapore (grants 0796/2003, 1176/2008, 1149/2008, STaR/0003/2008, 1249/2010, CG/SERI/2010, CIRG/1371/2013, and CIRG/1417/2015), and Biomedical Research Council, Singapore (08/1/35/19/550 and 09/1/35/19/616). C.Y.C is supported by an award from NMRC (CSA-SI/0012/2017).

SMHS. The study is supported by a grant (UM1CA173640) from the US National Institutes of Health. The authors thank the participants and staff members of the SMHS research team for their important contributions.

SMHS. The study is supported by a grant (UM1CA173640) from the US National Institutes of Health. The authors thank the participants and staff members of the SMHS research team for their important contributions.

SP2. The Singapore Prospective Study Program (SP2) was funded through grants from the Biomedical Research Council of Singapore (BMRC) and the National Medical Research Council of Singapore (NMRC). Genome Institute of Singapore provided services for genotyping.

SWHS. The SWHS is supported by the National Institutes of Health research grant UM1 CA182910. The authors thank the participants and staff members of the SWHS research for their important contributions.

TMM CommCohort Study. This work was supported (in part) by Tohoku Medical Megabank Project from MEXT, and Japan Agency for Medical Research and development, AMED (under Grant Number JP17km0105001).

TWSC. We acknowledge the members of the Translational Resource Center and the National Center for Genome Medicine at Academia Sinica for their support in subject recruitment, genotyping and statistical analysis. The TWSC study was supported by the Academia Sinica Genomic Medicine Multicenter Study, Taiwan.

REFERENCES

1. International Consortium for Blood Pressure Genome-Wide Association Studies *et al.* Genetic variants in novel pathways influence blood pressure and cardiovascular disease risk. *Nature* **478**, 103-9 (2011).
2. Ehret, G.B. *et al.* The genetics of blood pressure regulation and its target organs from association studies in 342,415 individuals. *Nat Genet* **48**, 1171-1184 (2016).
3. Surendran, P. *et al.* Trans-ancestry meta-analyses identify rare and common variants associated with blood pressure and hypertension. *Nat Genet* **48**, 1151-1161 (2016).
4. Liu, C. *et al.* Meta-analysis identifies common and rare variants influencing blood pressure and overlapping with metabolic trait loci. *Nat Genet* **48**, 1162-70 (2016).
5. Warren, H.R. *et al.* Genome-wide association analysis identifies novel blood pressure loci and offers biological insights into cardiovascular risk. *Nat Genet* **49**, 403-15 (2017).
6. Tabara, Y. *et al.* Association of Chr17q25 with cerebral white matter hyperintensities and cognitive impairment: the J-SHIPP study. *Eur J Neurol* **20**, 860-2 (2013).
7. Hirata, M. *et al.* Cross-sectional analysis of BioBank Japan clinical data: A large cohort of 200,000 patients with 47 common diseases. *J Epidemiol* **27**, S9-S21 (2017).
8. Takeuchi, F. *et al.* Blood pressure and hypertension are associated with seven loci showing male-specific interaction with age among the Japanese. *Circulation* **121**, 2302-2309 (2010).
9. Tsuchihashi-Makaya, M. *et al.* Gene-environmental interaction regarding alcohol-metabolizing enzymes in the Japanese general population. *Hypertens Res* **32**, 207–213 (2009).
10. Asano, H. *et al.* Plasma resistin concentration determined by common variants in the resistin gene and associated with metabolic traits in an aged Japanese population. *Diabetologia* **53**, 234-46 (2010).
11. Wang, Y.X. *et al.* Ocular diseases and 10-year mortality: the Beijing Eye Study 2001/2011. *Acta Ophthalmol* **92**, e424-8 (2014).

12. Popkin, B.M., Du, S., Zhai, F., Zhang, B. Cohort Profile: The China Health and Nutrition Survey--monitoring and understanding socio-economic and health change in China, 1989-2011. *Int J Epidemiol* **39**, 1435-40 (2010).
13. Adair, L.S. *et al.* Cohort profile: the Cebu longitudinal health and nutrition survey. *Int J Epidemiol* **40**, 619-25 (2011).
14. GenSalt Collaborative Research, G. GenSalt: rationale, design, methods and baseline characteristics of study participants. *J Hum Hypertens* **21**, 639-46 (2007).
15. Kim, Y.J. *et al.* Large-scale genome-wide association studies in East Asians identify new genetic loci influencing metabolic traits. *Nat Genet* **43**, 990-5 (2011).
16. Cho, Y.S. *et al.* A large-scale genome-wide association study of Asian populations uncovers genetic factors influencing eight quantitative traits. *Nat Genet* **41**, 527-34 (2009).
17. Win, A.M. *et al.* Patterns of physical activity and sedentary behavior in a representative sample of a multi-ethnic South-East Asian population: a cross-sectional study. *BMC Public Health* **15**, 318 (2015).
18. Ye, X. *et al.* Distributions of C-reactive protein and its association with metabolic syndrome in middle-aged and older Chinese people. *J Am Coll Cardiol* **49**, 1798-805 (2007).
19. Cui, J.S., Hopper JL, Harrap SB. Antihypertensive treatments obscure familial contributions to blood pressure variation. *Hypertension* **41**, 207-10 (2003).
20. Lavanya, R. *et al.* Methodology of the Singapore Indian Chinese Cohort (SICC) eye study: quantifying ethnic variations in the epidemiology of eye diseases in Asians. *Ophthalmic Epidemiol* **16**, 325-36 (2009).
21. Hankin, J.H. *et al.* Singapore Chinese Health Study: development, validation, and calibration of the quantitative food frequency questionnaire. *Nutr Cancer* **39**, 187-95 (2001).
22. Foong, A.W. *et al.* Rationale and methodology for a population-based study of eye diseases in Malay people: The Singapore Malay eye study (SiMES). *Ophthalmic Epidemiol* **14**, 25-35 (2007).
23. Shu, X.O. *et al.* Cohort Profile: The Shanghai Men's Health Study. *Int J Epidemiol* **44**, 810-8 (2015).

24. Hughes, K. *et al.* Cardiovascular diseases in Chinese, Malays, and Indians in Singapore. II. Differences in risk factor levels. *J Epidemiol Community Health* **44**, 29-35 (1990).
25. Cutter, J., Tan, B.Y. & Chew, S.K. Levels of cardiovascular disease risk factors in Singapore following a national intervention programme. *Bull World Health Organ* **79**, 908-15 (2001).
26. Hughes, K., Aw, T.C., Kuperan, P. & Choo, M. Central obesity, insulin resistance, syndrome X, lipoprotein(a), and cardiovascular risk in Indians, Malays, and Chinese in Singapore. *J Epidemiol Community Health* **51**, 394-9 (1997).
27. Zheng, W. *et al.* The Shanghai Women's Health Study: rationale, study design, and baseline characteristics. Am J Epidemiol. 2005 Dec 1;162(11):1123-31.
28. Kuriyama S *et al.* The Tohoku Medical Megabank Project: Design and Mission. *J Epidemiol* **26**, 493-511 (2016).
29. Pan, W.H. *et al.* Han Chinese cell and genome bank in Taiwan: purpose, design and ethical considerations. *Hum Hered* **61**, 27-30 (2006).
30. Chen, Z. *et al*; China Kadoorie Biobank (CKB) collaborative group. China Kadoorie Biobank of 0.5 million people: survey methods, baseline characteristics and long-term follow-up. *Int J Epidemiol* **40**, 1652–1656 (2011).
31. Yang, J., Zeng, J., Goddard, M. E., Wray, N. R. & Visscher, P. M. Concepts, estimation and interpretation of SNP-based heritability. *Nat Genet* **49,** 1304–1310 (2017).
32. Speed, D. *et al.* Reevaluation of SNP heritability in complex human traits. *Nat Genet* **49,** 986–992 (2017).
33. Gazal, S. *et al.* Linkage disequilibrium-dependent architecture of human complex traits shows action of negative selection. *Nat Genet* **49,** 1421–1427 (2017).
34. Finucane, H. K. *et al.* Partitioning heritability by functional annotation using genome-wide association summary statistics. *Nat Genet* **47,** 1228–1235 (2015).
35. Brown, B. C., Ye, C. J., Price, A. L. & Zaitlen, N. Transethnic Genetic-Correlation Estimates from Summary Statistics. *Am J Hum Genet* **99,** 76–88 (2016).
36. Bulik-Sullivan, B. K. *et al.* LD Score regression distinguishes confounding from polygenicity in genome-wide association studies. *Nat Genet* **47,** 291–295 (2015).
37. Chakravarti, A. *et al.* A global reference for human genetic variation. *Nature* **526,** 68–74 (2015).

38. Diaz, F. J. Identifying Tail Behavior by Means of Residual Quantile Functions. *J Comput Graph Stat* **8,** 493–509 (1999).

39. Meuwissen, T., Hayes, B. & Goddard, M. Accelerating Improvement of Livestock with Genomic Selection. *Annu Rev Anim Biosci* **1,** 221–237 (2013).
