# Supplementary material for: Interethnic analyses of blood pressure loci in populations of East Asian and European descent
Source: Nat Commun. 2018 Nov 28;9:5052. doi: 10.1038/s41467-018-07345-0 (PMC6261994; doi:10.1038/s41467-018-07345-0)
Supplement: Supplementary file 3 — Description of Additional Supplementary Files [file 41467_2018_7345_MOESM3_ESM.pdf]

Description of Additional Supplementary Files

File Name: Supplementary Data 1

Description: Trait-specific sentinel SNPs reaching $P < 5 \times 10^{-8}$ in East Asians after combined analysis. Results from the GWAS stage-1 are combined with those from the stage-2 meta-analyses. Effects are given as beta coefficients per allele copy from linear regression (mmHg, SBP, DBP, MAP, PP) or logistic regression (log-Odds, HT).

File Name: Supplementary Data 2

Description: Blood pressure association of 19 novel sentinel SNPs in East Asian and European populations for GWAS and replication study. Lookup results of the novel SNPs in East Asian and European populations are shown for replication study.

File Name: Supplementary Data 3

Description: Association of 19 novel sentinel SNPs with the five blood pressure phenotypes in East Asian combined analysis of GWAS stages 1+2.

File Name: Supplementary Data 4

Description: Results in the GWAS stage-1 for SNPs previously reported to be associated with blood pressure phenotypes in GWASs.

File Name: Supplementary Data 5

Description: Genetic associations previously reported for the traits other than blood pressure at the newly identified loci. SNPs in strong LD ($r^2 \geq 0.95$) with the sentinel SNP are shown in the table.

File Name: Supplementary Data 6

Description: Genetic loci showing significant heterogeneity of genetic impact on SBP between the two ethnic groups.

File Name: Supplementary Data 7

Description: Interethnic comparability of genetic impact on blood pressure at 48 loci containing ancestry-specific SNPs. Refer to the Method section about the exploration of transethnic SNPs forming a haplotype shared between ethnic groups (haplo-SNPs) and alternate ancestry-specific SNPs, and

Supplementary Figure 6b about the interpretation of interethnic comparability.

File Name: Supplementary Data 8

Description: Interethnic heterogeneity of genetic impact on blood pressure at non-rare blood pressure loci previously reported and newly identified. See Methods about the details. Significant ($P_{hetero} < 2.1 \times 10^{-4}$) SNPs are shown in bold letter. Genetic impacts for 242 SNPs in the table are also plotted in Supplementary Figure 10.

File Name: Supplementary Data 9

Description: Ancestry-specific loci for complex traits. The list of ancestry-specific loci is selected such that the sentinel SNPs at the corresponding loci reach genome-wide significance ($P < 5 \times 10^{-8}$) in one ethnic group but are non-polymorphic or rare (MAF < 0.05) in another ethnic group.
